# A systematic review of possible serious adverse health effects of nicotine replacement therapy

**DOI:** 10.1007/s00204-016-1856-y

**Published:** 2016-10-03

**Authors:** Peter N. Lee, Marc W. Fariss

**Affiliations:** 1 P N Lee Statistics and Computing Ltd, 17 Cedar Road, Sutton, Surrey SM2 5DA UK; 2 Altria Client Services LLC, Richmond, VA USA

**Keywords:** Nicotine replacement therapy, Nicotine, Epidemiology, Clinical trials, Systematic review, Serious adverse health effects

## Abstract

We conducted a systematic literature review to identify and critically evaluate studies of serious adverse health effects (SAHEs) in humans using nicotine replacement therapy (NRT) products. Serious adverse health effects refer to adverse events, leading to substantial disruption of the ability to conduct normal life functions. Strength of evidence evaluations and conclusions were also determined for the identified SAHEs. We evaluated 34 epidemiological studies and clinical trials, relating NRT use to cancer, reproduction/development, CVD, stroke and/or other SAHEs in patients, and four meta-analyses on effects in healthy populations. The overall evidence suffers from many limitations, the most significant being the short-term exposure (≤12 weeks) and follow-up to NRT product use in most of the studies, the common failure to account for changes in smoking behaviour following NRT use, and the sparse information on SAHEs by type of NRT product used. The only SAHE from NRT exposure we identified was an increase in respiratory congenital abnormalities reported in one study. Limited evidence indicated a *lack* of effect between NRT exposure and SAHEs for CVD and various reproduction/developmental endpoints. For cancer, stroke and other SAHEs, the evidence was inadequate to demonstrate any association with NRT use. Our conclusions agree with recent statements from authoritative bodies.

**Electronic supplementary material:**

The online version of this article (doi:10.1007/s00204-016-1856-y) contains supplementary material, which is available to authorized users.

## Introduction

The active ingredient in pharmaceutical nicotine replacement therapy (NRT) products is nicotine. Millions of people are exposed daily to nicotine by using these smoking cessation products (Royal College of Physicians 2016; Siahpush et al. 2015). In this review, pharmaceutical NRTs are products regulated as drugs or medicines to aid smoking cessation (Royal College of Physicians 2016; US Food and Drug Administration 2013). Pharmaceutical NRT products have been available, for over three decades, with current forms such as chewing gum, lozenge, oral tablet, skin patch, nasal spray and inhaler (Ferguson et al. 2011; Royal College of Physicians 2016). Because daily exposure to these products depends on individual preferences and needs, a broad range of maximum plasma nicotine levels from 6 to 43 ng/ml has been reported (Haussmann and Fariss 2016; Schneider et al. 2001; Shiffman et al. 2005). In the USA, the recommended duration of use for NRT products is 8–12 weeks depending on the product form (US Food and Drug Administration 2013). However, the US Food and Drug Administration (FDA) recently proposed the possibility of a 6-month extension of NRT use limits with healthcare provider consultation (Fucito et al. 2014; US Food and Drug Administration 2013). It seems that uncertainty regarding the potential adverse health effects of long-term use of NRT products (such as cancer) may be, in part, responsible for the proposed increase in duration of use (from 3 to 6 months) being relatively modest (Shields 2011; US Surgeon General 2014). In other countries, including the UK, the medicines regulatory authorities appear to have a more relaxed approach to the long-term use of pharmaceutical NRT products. For example, since 2010, UK smokers have been encouraged to continue NRT use if needed to maintain smoking cessation. In other words, the consumer should decide how long to continue using NRT products (Kostygina et al. 2016; McNeill et al. 2001; Royal College of Physicians 2016).

Nicotine *per se* is a unique active ingredient for a consumer product in that the majority of nicotine’s effects are mediated by binding and activating nicotinic acetylcholine (nACh) receptors in a wide variety of neuronal (central and peripheral nervous system) and non-neuronal tissue. Consequently, nicotine exposure affects numerous systems, including neurological, neuromuscular, cardiovascular, respiratory, immunological and gastrointestinal. The presence of different types of nACh receptors, receptor up-regulation and receptor desensitization influences these complex physiological effects (Lam et al. 2016; Marks et al. 1987; Renda and Nashmi 2014). Evidence from experimental animal models clearly demonstrate nicotine’s ability to enhance existing tissue injury and diseases such as cancer, cardiovascular disease, stroke, pancreatitis, peptic ulcer, renal injury and developmental (e.g. pulmonary, reproductive and central nervous system) abnormalities (Arany et al. 2011; Bruin et al. 2010; Chowdhury et al. 1995; Hall et al. 2016; Haussmann and Fariss 2016; Lau et al. 2006; Qiu et al. 1991; Rehan et al. 2012; Wang et al. 1997). These reported adverse health effects were observed following short-term exposure to nicotine per se (<12 weeks) and appear to be dependent on nicotine activation of nACh receptors in the affected tissue.

In regard to potential serious adverse health effects (SAHEs) associated with long-term use of pharmaceutical NRT products in humans, the United Kingdom’s National Institute for Health and Care Excellence (NICE) concluded that “evidence is available from studies with up to 5-year follow-up which suggests that ‘pure’ nicotine, in the form available in NRT products, does not pose a significant health risk” (National Institute for Health and Care Excellence 2013). In addition, a recent Surgeon General’s report concluded that inadequate evidence is available “to infer the presence or absence of a causal relationship between exposure to nicotine and risk of cancer” (US Surgeon General 2014). In contrast to these authoritative statements, FDA classifies NRT therapies as a Pregnancy Category “C” or “D” developmental hazard. Category D classification indicates “studies, adequate well-controlled or observational, in pregnant women have demonstrated a risk to the fetus” (Meadows 2001). Furthermore, potential SAHEs with pharmaceutical NRT product use are suggested based on government warning statements for such products. These include warnings against potential major adverse effects in the cardiovascular system (heart disease), gastrointestinal system (stomach ulcers) and diabetes with NRT product use (US Food and Drug Administration 2015a). Unfortunately, a systematic review of the scientific evidence related to potential SAHEs of pharmaceutical NRT products is not available.

The work described here has two main objectives: to identify and critically evaluate relevant studies pertaining to SAHEs in humans, if any, of pharmaceutical NRT and to provide strength of evidence evaluation and conclusions for these effects. For this review, we did not consider evidence relating SAHEs from exposure to snus (or any other smokeless tobacco product) or electronic nicotine delivery systems (ENDS). Currently, these commercial products are regulated as tobacco products and are not developed, approved and licensed as drugs or medicines to aid smoking cessation (Royal College of Physicians 2016; US Food and Drug Administration 2016). The potential adverse health effects of snus have been well studied (Lee 2011, 2013), and ENDS are relatively new products with tremendous diversity in product design and aerosol delivery. Finally, the words pharmaceutical NRT and NRT are used synonymously in this review.

## Methods

### Inclusion/exclusion criteria

Attention was restricted to epidemiological studies and clinical trials of NRT describing results relating to SAHEs. For this review, SAHEs are defined as adverse events leading to substantial disruption of the ability to conduct normal life functions, including those that lead to hospitalization, significant disability or birth defects, are life-threatening or result in death or require medical intervention to prevent one of the above outcomes (Little and Ebbert 2015; US Food and Drug Administration 2013). These do not include acute side effects such as nausea, vomiting and altered heart rate. Relevant reviews were also sought, partly to look for relevant publications, and partly for citation in this report. No attempt was made to identify individual clinical trials of smoking cessation in healthy individuals, since use of NRT is usually short term and serious adverse events are rare. Rather, we sought published meta-analyses of such trials, although also considering meta-analyses which included a small proportion of studies in diseased subjects.

Clinical and epidemiological studies in which pharmaceutical NRT products were used are included. In this review, pharmaceutical NRTs are products regulated as drugs or medicines to aid smoking cessation (Royal College of Physicians 2016; US Food and Drug Administration 2013). Studies that use any product that contains tobacco are excluded as they are regulated as tobacco products. Epidemiological studies on Swedish snus (a smokeless tobacco) are often used as an indirect measure of the potential adverse health effects of long-term nicotine use in humans (Benowitz 2011). However, for this review, exposure to NRT products and snus (or any other smokeless tobacco product) is *not* considered equivalent. NRT products are approved or licensed (regulated) as drugs or medicines to aid in smoking cessation, whereas snus and other smokeless tobacco products are regulated as tobacco products (Royal College of Physicians 2016; US Food and Drug Administration 2016). Likewise, studies that use ENDS products are excluded as they are currently undergoing regulatory consideration as tobacco products, not as drugs or medicines for aiding smoking cessation (Royal College of Physicians 2016; US Food and Drug Administration 2016).

Relevant publications were subdivided by type of study (epidemiological, clinical trials) and outcome [cancer, reproductive/developmental, cardiovascular disease (CVD), stroke, other SAHEs seen in patients, and other SAHEs seen in healthy populations].

### Literature searches

The first step was a PubMed search on 24 July 2015, using the terms:

(“NRT”[All Fields] OR “nicotine replacement therapy”[All Fields] OR “Nicotine chewing gum”[All Fields] OR “Nicotine patch”[All Fields] OR “Tobacco use cessation”[All Fields] OR “Tobacco use Cessation/methods”[All Fields]) AND (“Kidney disease”[All Fields] OR “Diabetes”[All Fields] OR “GI tract”[All Fields] OR “Stomach”[All fields] OR “Pancreas”[All Fields] OR “Pancreatic”[All Fields] OR “Reproductive”[All Fields] OR “Reproduction”[All Fields] OR “Pregnancy Complications”[Mesh terms] OR “Pregnancy Outcome”[Mesh terms] OR “Birth weight”[Mesh terms] OR “Infant, low birth weight”[Mesh terms] OR “Infant, newborn, diseases”[Mesh terms] OR “child development”[Mesh terms] OR “Cardiovascular disease”[All Fields] OR “Stroke”[All Fields] OR “Cancer”[All Fields]) AND ((“1960/01/01”[PDAT]: “3000/12/31”[PDAT]) AND “humans”[MeSH Terms]).

Abstracts of papers and reviews identified were inspected, with reasons for rejection noted for those clearly irrelevant, and publications of potential interest obtained. Papers read were either accepted or rejected with reasons noted (Table 1).

**Table 1:** Literature searches and reasons for rejection

Step	Result	*N*	Reasons for rejection^a^/papers completed
PubMed search on 24 July 2015	811 hits		
Abstracts examined	757 rejected	36	NRT not abbreviation for nicotine replacement therapy
		9	Study of e-cigarettes
		187	Not a study of nicotine replacement therapy
		10	*In vitro* or animal study
		185	Study of smoking cessation (or cutting down) with no relevant endpoints
		95	Endpoints not considered serious health effects
		190	Review or commentary not providing relevant data
		45	Other reasons (including case reports)
Papers examined	25 rejected^b^	8	No relevant endpoints
		13	Review with no new data or citations
		3	Case reports
		1	No appropriate comparison groups
Papers examined	29 accepted	23	Papers: Berlin et al. (2014), Carandang et al. (2011), Coleman et al. (2012b), Cooper et al. (2014a), Dhalwani et al. (2015), El-Mohandes et al. (2013), Elzi et al. (2006), Gaither et al. (2009), Greenland et al. (1998), Hubbard et al. (2005), Lassen et al. (2010), Lee et al. (2013), Murray et al. (2009), Oncken et al. (2008), Panos et al. (2010), Pollak et al. (2007), Schroeder et al. (2002), Strandberg-Larsen et al. (2008), Swamy et al. (2009), Tappin et al. (2015), Thomsen et al. (2010), Torp-Pedersen et al. (2010), Zhu et al. (2014)
		6	Reviews: Bruin et al. (2010), Coleman et al. (2011, 2012a), Forinash et al. (2010), Mills et al. (2014), Rore et al. (2008)
Cochrane reviews	1 accepted		Stead et al. (2012)
Secondary references	22 found		
Papers examined	7 rejected^c^	5	No relevant endpoints
		1	Review with no new data or citations
		1	Effects of smoking and nicotine replacement therapy indistinguishable
Papers examined	15 accepted		Cooper et al. (2014b), Joseph et al. (1996), Kapur et al. (2001), Kimmel et al. (2001), Meine et al. (2005), Milidou et al. (2012), Mills et al. (2010), Mohiuddin et al. (2007), Moore et al. (2009), Morales-Suarez-Varela et al. (2006), Paciullo et al. (2009), Rennard et al. (1994), Tzivoni et al. (1998), Wisborg et al. (2000) and Woolf et al. (2012)
PubMed search on 30 November 2015	46 hits		
Abstracts examined	44 rejected	13	Paper previously considered
		3	NRT not abbreviation for nicotine replacement therapy
		1	Study of e-cigarettes
		1	*In vitro* or animal study
		15	Study of smoking cessation (or cutting down) with no relevant endpoints
		4	Endpoints not considered serious health effects
		5	Review or commentary not providing relevant data
		2	Other reasons (including case reports)
Papers examined	2 rejected^d^	1	Commentary on paper we had with no new data
		1	Study of Medicaid not NRT

^a^Note that only one reason for rejection was applied to an abstract or paper, although, particularly for the abstracts, more than one reason may have applied

^b^The 25 papers rejected were Anthonisen et al. (2005), Benowitz and Gourlay (1997), Cordier (2014), Dempsey and Benowitz (2001), Ebbert et al. (2007), Ford and Zlabek (2005), Forest (2010), Harrell et al. (2014), Hughes (2000), Jacobson and Jaklitsch (2013), Joseph and Fu (2003), Kotz et al. (2015), Lancaster (2014), Lavelle et al. (2003), Marsh et al. (2005), Myung et al. (2007), Nicholson et al. (2010), Osadchy et al. (2009), Pauly and Slotkin (2008), Reuther and Brennan (2014), Shahab and Goniewicz (2014), Smith et al. (2002), Steinberg et al. (2009), Weinberger et al. (2014), Zatoński and Zatoński (2015)

^c^The 7 papers rejected were Aubin et al. (2008), Eliasson et al. (1996), Haustein et al. (2002), Oncken et al. (1996, 1997), Shiffman et al. (2002), Tappin et al. (2015)

^d^The 2 papers rejected were Black et al. (2014) and Adams et al. (2013)

Relevant clinical trial reviews in the Cochrane library were also obtained. Reference lists of accepted papers and reviews were then examined and further papers obtained and read, with additional papers accepted and reasons for rejection noted for others (Table 1).

Near finalization of the paper an additional PubMed search was conducted on 30 November 2015 using the same search terms described above.

### Assessment of studies

For each topic, a critical assessment form (CAF) was completed for each publication. Notes for completing CAFs are given in Supplementary File 1, together with the completed CAFs. Each form provides a detailed summary of study design, findings and strengths and weaknesses.

There are two types of CAF. The first, for publications describing results of individual studies, is divided into 18 sections: form no.; topic; author(s); title; source; study type; study location; population studied and inclusion criteria; nicotine exposures; treatment groups and sizes; relevant endpoints; confounding variables; other relevant study details; relevant findings; authors’ main relevant conclusions; strengths and weaknesses mentioned; study quality score (for epidemiology studies) or risk of bias (for clinical trials); and comments.

The second, for publications describing results of meta-analyses of SAHEs in clinical trials of healthy people, is divided into 17 sections: form no.; topic; author(s); title; source; meta-analysis type; location of studies; populations studied and inclusion criteria; searches; nicotine exposures; numbers of subjects considered; relevant endpoints; relevant findings; authors’ main relevant conclusions; study quality assessment; strengths and weaknesses mentioned; and comments.

The CAFs are presented in Supplementary File 1 separately by topic (e.g. cancer and reproduction/development) and are numbered consecutively. Exceptionally, where one publication provides results for multiple topics, the CAFs use versions labelled, e.g. 28A, 28B and 28C.

Study quality was assessed as good, fair or poor based on the NIH published quality assessment tools for observational cohort and cross-sectional studies (National Heart Lung and Blood Institute 2014b) and case–control studies (National Heart Lung and Blood Institute 2014a). For clinical trials, risk of bias was assessed using the Cochrane Collaboration’s tool (Higgins et al. 2011). Supplementary File 2 summarizes methods used for both assessment types and gives detailed results for each study. Supplementary Files 3, 4 and 5 give the results from the assessments for, respectively, reproduction/development, CVD and other SAHEs in patients. Supplementary File 6 gives assessments for the meta-analyses of other SAHEs.

### Summarizing results for an endpoint

The method varies with the extent of available data and includes a simple textual description, a table of results and/or a meta-analysis. Conducting a meta-analysis requires at least three studies reporting relevant results for the endpoint, but also on the results being reported similarly enough to allow combination over studies.

Results presented are always given with the NRT exposed group as the test group and the non-exposed as the comparison group. Thus, estimates of the RR, odds ratio (OR) or hazard ratio (HR) always relate to the NRT/non-NRT ratio, and mean differences are always NRT minus non-NRT. Generally, the analysis is restricted to those who have smoked, though exceptions where the source paper does not allow this are indicated as appropriate. Often, the NRT versus non-NRT comparison statistic has to be estimated from the source publication using standard methods. Examples where this is necessary include estimating the unadjusted RR and its 95 % confidence interval (CI) in a clinical trial where numbers affected and at risk are available for the NRT and placebo groups; estimating unadjusted mean differences and 95 % CIs from group-specific means and standard deviations; estimating CIs from means and *p* values; and estimating RRs, ORs or HRs and their 95 % CIs for a more relevant comparison than that reported (e.g. relative to never smokers).

### Strength of evidence assessment

This was carried out for each endpoint, and overall for the topic. The criteria for evaluating specific SAHEs were adapted and modified from those outlined by the International Agency for Research on Cancer (IARC) (International Agency for Research on Cancer 2007). A major modification to the IARC strength of evidence assessment is the separation of their classification “evidence suggesting lack of carcinogenicity” into “limited evidence suggesting a lack of effect” and “sufficient evidence of a lack of effect”. With this revised classification in place, the strength of evidence conclusions can be neutral (inadequate evidence) or can be deemed limited or sufficient in both directions, for an adverse effect or a lack of an adverse effect (Haussmann and Fariss 2016). The criteria used were as follows.

*Sufficient evidence*: A causal relationship has been established in humans between exposure to NRT and this SAHE in humans. A positive association was observed in which chance, bias and confounding factors could be ruled out with reasonable confidence. Conclusive studies have been conducted.

*Limited evidence*: A positive association was observed between exposure to NRT and this SAHE, but chance, bias and confounding factors could not all be ruled out with reasonable confidence. Conclusive studies are lacking.

*Inadequate evidence*: The available studies are of insufficient quality, consistency or statistical power to permit a conclusion regarding a positive association between exposure to NRT and this SAHE. This category includes no data or conflicting evidence in multiple studies.

*Limited evidence suggesting a lack of effect*: A statistically significant association was not observed between exposure to NRT and this SAHE, but a true relationship could not be ruled out with reasonable confidence because of a lack of statistical power, bias and/or confounding.

*Sufficient evidence of a lack of effect*: A lack of association has been observed between exposure to NRT and this SAHE, based on studies of adequate quality, consistency and statistical power. The possibility of a very small risk at relevant exposure levels can never be excluded.

## Results

### Literature searches

Table 1 summarizes results of the original searches. Forty-four relevant publications were found, six being reviews used only for searching for secondary references. Of the rest, one related to cancer, 19 to reproduction/development, 10 to CVD, three to stroke, five to other SAHEs on patients, and four were meta-analyses providing results for other SAHEs seen in healthy populations, some publications providing results for more than one endpoint. The additional PubMed search cited 33 more references, but none relevant to this review.

### Assessment of studies

Supplementary File 1 gives the completed CAFs, and Supplementary File 2 study-specific information on study quality and risk of bias. Of the 12 epidemiological studies described, two were classified as of “good” quality, eight as “fair” and two as “poor”. Of the 14 clinical trials, eight were considered to have a “low” risk of bias, five a “high” risk, with one classified as “unclear”.

While details of the studies are described by topic below, it should be noted that the follow-up period was generally very short. The longest was the 7½-year follow-up for cancer of participants in the Lung Health Study (Murray et al. 2009) and the follow-up until 2011 for investigating attention-deficit/hyperactivity disorder (ADHD) of children born between 1996 and 2003 in the Danish National Birth Cohort study (Zhu et al. 2014). A follow-up until 2006 for investigating strabismus in the same study (Torp-Pedersen et al. 2010) and two-year follow-ups in two clinical trials (Cooper et al. 2014b; Mohiuddin et al. 2007) were the only other studies appearing to involve more than one year of follow-up.

### Type of NRT

Of the 12 epidemiological studies, five (Carandang et al. 2011; Kimmel et al. 2001; Meine et al. 2005; Paciullo et al. 2009; Panos et al. 2010) only considered nicotine patches, while one (Murray et al. 2009) only considered nicotine gum. The remainder considered multiple, or any type of NRT, though results by type of NRT (gum, patch, inhaler) were only reported in two publications from the Danish Birth Cohort (birthweight—Lassen et al. (2010) and stillbirth rate in Strandberg-Larsen et al. (2008)) and in the UK case series analysis (acute myocardial infarction [AMI], stroke and death—(Hubbard et al. 2005)).

Of the 14 clinical trials, three trials (Pollak et al. 2007; Swamy et al. 2009; Thomsen et al. 2010) allowed the subjects allocated NRT to choose the type they preferred, one (Mohiuddin et al. 2007) did not define the type and one (Oncken et al. 2008) allocated subjects to gum. The remainder allocated subjects to nicotine patches. Results by type of NRT product were never reported in any clinical trial.

Of the four meta-analyses considered, one (Greenland et al. 1998) only concerned patches, the others considering results from multiple studies, each involving allocation to one or more NRT types. The limited evidence on risk of SAHEs by NRT type reported in these meta-analyses and in the epidemiological studies is considered in the following sections.

### Cancer

Based on findings from animal and mechanistic studies, a case for biological plausibility has been proposed for a potential role of nicotine in carcinogenesis (Cardinale et al. 2012; Grando 2014; Haussmann and Fariss 2016; Improgo et al. 2011). Carcinogenesis is a multistage process that involves three stages: initiation, promotion and progression (Klaunig 2013). For initiation, a substance must exhibit genotoxic properties which have been reported for nicotine at concentrations relevant to NRT use (Haussmann and Fariss 2016). Similarly, nicotine has been reported to stimulate cell proliferation, inhibit apoptosis, induce angiogenesis and inhibit immune function (Cardinale et al. 2012; Grando 2014). Thus, there is considerable evidence that nicotine exposure (at levels relevant to NRT users) can affect many of the cellular processes that are considered important for the initiation, promotion or progression of the carcinogenic process. In fact, a recent comprehensive review concludes that a majority of studies provides sufficient evidence for an association between short-term nicotine exposure and enhanced carcinogenesis of cancer cells inoculated in mice (Haussmann and Fariss 2016). The results from these non-clinical studies clearly support investigating similar nicotine effects with NRT use, especially in smokers or former smokers where initiated cancer cells may be present.

For NRT users, only one useful publication was found (Murray et al. 2009)—see CAF 1. This described follow-up of participants in the Lung Health Study, an RCT of middle-aged volunteers with asymptomatic airways obstruction randomized to a smoking intervention involving encouragement to use nicotine gum. The 7½-year follow-up started after the 5-year intervention period and compared cancer risk in 3315 participants alive and cancer-free at start of follow-up by NRT use in the preceding 5 years. The study quality was rated “good”.

After adjustment for baseline age, sex, cigarettes per day and lifetime pack-years smoking, NRT use was unrelated to lung cancer, gastrointestinal (GI) cancer or all cancer (see Table 2), regardless of adjustment for pack-years cigarette use in the 5 years following randomization. Nor were relationships seen with NRT use when mean daily use was replaced by any NRT use (results not shown). In contrast, pack-years cigarette use was significantly related to lung cancer risk, whether or not adjusted for daily NRT use. The authors noted that the results add “credence to our conclusion that nicotine replacement therapy does not cause cancer”.

**Table 2:** Results summary for NRT and cancer

CAF no.^a^	References	Exposure	Endpoint	Cases^b^	Hazard ratio^c^ (95 % CI)	Adjustments
1	Murray et al. (2009)	Mean daily NRT use	Lung cancer	75	1.02 (0.95–1.09)	^d^
					1.04 (0.97–1.12)	^e^
			GI cancer	33	0.97 (0.86–1.10)	^d^
					0.97 (0.82–1.14)	^e^
			All cancer	203	1.00 (0.96–1.05)	^d^
					1.01 (0.97–1.06)	^e^

^a^ *CAF* critical assessment form. CAF 1 is a prospective study

^b^During the 7.5-year follow-up period following the 5-year period of the Lung Health Study

^c^Per piece of gum used per day on average during the Lung Health Study

^d^Baseline age, sex, cigarettes per day and lifetime pack-years of smoking

^e^Also adjusted for pack-years cigarette use over the 5-year period of the Lung Health Study

Much more limited evidence comes from a 12-month follow-up study of HIV-infected individuals (Elzi et al. (2006), CAF 24). This study reported one lung cancer death among 34 smokers participating in a smoking cessation program where NRT was made available, and one among 383 non-participating smokers, a comparison which is not statistically significant at *p* < 0.05, or adjusted for the participants having higher baseline cigarette consumption and disease rates than non-participants.

Given the few publications, the limited follow-up for a chronic disease like cancer, and the difficulty of reliably disentangling effects of NRT and smoking, we consider there is inadequate evidence to permit a conclusion regarding an association between exposure to NRT and cancer.

### Reproduction/development

Based on findings from published animal studies, sufficient evidence supports an association between short-term nicotine exposure and adverse reproductive and developmental effects (Bruin et al. 2010; Hall et al. 2016; Rehan et al. 2012; Slotkin 2008; Spindel and McEvoy 2016; Wong et al. 2015). This is not surprising since nicotine binds and activates nAChRs, mimicking the effects of the endogenous ligand for this receptor, acetylcholine. It is well known that during the development of the central nervous system, neurotransmitters such as acetylcholine play a critical role in brain assembly from early embryonic stages to early adulthood (Slotkin 2008). These nicotinic acetylcholine receptors also play an important role in coordinating the development of other organ systems including reproductive organs and the lung (Bruin et al. 2010; Rehan et al. 2012; Spindel and McEvoy 2016). As a result, animal studies have consistently reported that prenatal exposure to nicotine can result in deficits in reproductive function (Bruin et al. 2010; Wong et al. 2015), behavioural and cognitive dysfunction such as hyperactivity, cognitive impairment, increased anxiety (Hall et al. 2016; Pauly and Slotkin 2008) as well as pulmonary effects such as impaired lung development and function (Rehan et al. 2012; Spindel and McEvoy 2016). For humans, there are conflicting views about the safe use of pharmaceutical NRT in pregnant women. Warnings from the US Food and Drug Administration place NRT therapies in Pregnancy Categories “C” or “D” as developmental hazards (Slotkin 2008). In contrast, guidance from the UK Committee on Safety of Medicines encourages the use of pharmaceutical NRT products for smoking cessation during pregnancy (Pauly and Slotkin 2008). Thus, the apparent uncertainty in humans and the adverse effects clearly observed in animal studies supports the need for a comprehensive review of studies investigating reproductive/developmental effects in offspring of NRT users.

As summarized in Supplementary File 3, eight publications based on epidemiological studies provided information on NRT and reproduction/development. Fuller study details are presented in CAFs 2 to 9. A cross-sectional study of women interviewed post-natally (Gaither et al. (2009), CAF 8, study quality “fair”) related NRT use in pregnancy to low birthweight and preterm birth of their offspring. Another study (Dhalwani et al. (2015), CAF 9, “fair”) used mother–child primary care records of children born in the UK to relate NRT prescription to incidence of major congenital abnormalities. The remainder (Lassen et al. (2010); Milidou et al. (2012); Morales-Suarez-Varela et al. (2006); Strandberg-Larsen et al. (2008); Torp-Pedersen et al. (2010); Zhu et al. (2014), CAFs 2 to 7, all “good”) derived from the Danish National Birth Cohort, each concerning different endpoints. The analyses all involve births in 1996 to 2003, though the publications vary in inclusion/exclusion criteria, and the comparison groups used for assessing NRT effects, some only reporting risks for women who used NRT and did not smoke. Common weaknesses of some of the analyses include the few relevant events in NRT users and the failure to control for changes in smoking behaviour after starting NRT.

Supplementary File 3 also summarizes 11 publications relating to eight clinical trials of smoking cessation (CAFs 10 to 20). Two (CAFs 13, 15) concern a multicentre trial in North Carolina (risk of bias “unclear”), one (Pollak et al. 2007) reporting results for various endpoints, the other (Swamy et al. 2009) presenting a detailed analysis of adverse events following medical record review. Three (CAFs 16, 19 and 20) concern a multicentre trial in England (risk of bias “low”), Coleman et al. (2012b) giving results for various endpoints recorded at or before birth, Cooper et al. (2014b) reporting on infant and maternal outcomes at 2 years, and Cooper et al. (2014a) presenting an extremely detailed report, but providing no additional relevant results. This is by far the largest study. Of the remaining six studies, two provide little useful information, one (Kapur et al. (2001), CAF 11, risk of bias “low”) terminated prematurely, the other (Schroeder et al. (2002), CAF 12, risk of bias “high”) being small, with no controls. Of the other four studies, one (Oncken et al. (2008), CAF 14, risk of bias “low”) compared nicotine and placebo gum, two (Berlin et al. (2014) and Wisborg et al. (2000), CAFs 10 and 18, both risk of bias “low”) compared nicotine and placebo patches, and one (El-Mohandes et al. (2013), CAF 17, risk of bias “low”) compared cognitive behavioural therapy alone and in conjunction with nicotine patches. A similar comparison was made in the North Carolina study (Pollak et al. 2007; Swamy et al. 2009). The studies provide results for a various endpoints, considered in turn below.

*Fetal loss and spontaneous abortion* (Table 3): Each effect estimate is based on few exposed cases, none being significant (at *p* < 0.05). For stillbirth/fetal loss, the meta-analysis estimate is 0.78 (95 % CI 0.45–1.33), with no heterogeneity. Stillbirth risk was also noted to be unaffected by type of NRT in one study (Strandberg-Larsen et al. 2008).

**Table 3:** Results summary for NRT and reproduction/development—fetal loss and spontaneous abortion

CAF no.^a^	References	Comparison^b^	Endpoint	Cases^c^	HR/RR/OR (95 % CI)^d^
3	Strandberg-Larsen et al. (2008)	Any NRT versus no NRT	Stillbirth	8/487	0.57 (0.28–1.16)^e^
13	Pollak et al. (2007)	Any NRT + CBT versus CBT only	Fetal loss	3/2	0.77 (0.13–4.48)^f^
14	Oncken et al. (2008)	Nicotine versus placebo gum	Fetal loss	2/1	1.79 (0.17–19.44)^f^
			Spontaneous abortion	2/0	4.49 (0.22–92.19)^f^
16	Coleman et al. (2012b)	Nicotine versus placebo patch	Miscarriage	3/2	1.52 (0.25–9.13)^g^
			Stillbirth	5/2	2.59 (0.50–13.40)^g^
18	Berlin et al. (2014)	Nicotine versus placebo patch	Stillbirth	4/5	0.80 (0.22–2.94)^f^
			Total fetal death	8/7	1.14 (0.42–3.09)^f^
Meta-analysis of stillbirth/fetal loss			Fixed effect		0.78 (0.45–1.33)
			Random effects		0.78 (0.45–1.33)
			Heterogeneity *p*		0.51

^a^ *CAF* critical assessment form. CAF 3 is a prospective study, the others are clinical trials

^b^ *CBT* cognitive behavioural therapy

^c^Exposed/unexposed cases

^d^ *HR* hazard ratio, *OR* odds ratio, *RR* relative risk

^e^HR adjusted for maternal age, socio-occupational status and smoking. The HR was noted to be unaffected by type of NRT used

^f^Unadjusted RR estimated from data provided

^g^OR adjusted for recruitment centre

*Birthweight* (Table 4): Eight studies provide relevant data, one clinical trial giving results in two publications (Pollak et al. 2007; Swamy et al. 2009). Seven studies provided heterogeneous (*p* = 0.01) estimates for risk of low birthweight, Oncken et al. (2008) reporting a highly significant (*p* < 0.01) decreased risk of nicotine gum and the others no significant differences. A random-effect meta-analysis showed no overall effect, RR 0.81 (95 % CI 0.48–1.39). Five trials compared mean birthweight in NRT and placebo groups. Substantially higher birthweights associated with NRT exposure, by 200 g or more, were seen in three studies, significantly so (at *p* < 0.05) in two (Oncken et al. 2008; Wisborg et al. 2000), but not in the other (El-Mohandes et al. 2013). Smaller, non-significant, differences were seen in the other trials (Berlin et al. 2014; Pollak et al. 2007) and also in an epidemiological study (Lassen et al. 2010), which expressed the birthweight change per week of NRT use, and also found no significant variation by type of NRT. The inverse-variance weighted mean difference associated with NRT use was estimated as 142 (95 % CI −53 to 336) grams.

**Table 4:** Results summary for NRT and reproduction/development—birthweight or low birthweight

CAF no.^a^	References	Comparison^b^	Endpoint	Cases^c^	Statistic (95 % CI)^d^
4	Lassen et al. (2010)	–	Birthweight change (g) per week NRT use	–	0.25 (−2.31 to 2.81)^e^
8	Gaither et al. (2009)	Prescribed/recommended NRT versus not	Low birthweight	NA	1.49 (0.79 to 2.82)^f^
10	Wisborg et al. (2000)	Nicotine versus placebo patch	Birthweight difference (g)	–	186 (35 to 336)^g^
			Low birthweight	4/11	0.4 (0.1–1.1)^i^
13	Pollak et al. (2007)	Any NRT + CBT versus CBT only	Birthweight difference (g)	–	−71 (−283 to 141)^h^
14	Oncken et al. (2008)	Nicotine versus placebo gum	Birthweight difference (g)	–	337 (155 to 519)^h^
			Low birthweight	2/16	0.11 (0.03–0.47)^i^
15	Swamy et al. (2009)	Any NRT + CBT versus CBT only	Low birthweight	4/0	4.48 (0.25–81.63)^i^
16	Coleman et al. (2012b)	Nicotine versus placebo patch	Low birthweight	56/43	1.38 (0.90–2.09)^j^
17	El-Mohandes et al. (2013)	Nicotine patch + CBT versus CBT only	Birthweight difference (g)	–	206 (−98 to 510)^h^
			Low birthweight	3/4	0.75 (0.19 to 3.03)^i^
18	Berlin et al. (2014)	Nicotine versus placebo patch	Birthweight difference (g)	–	50 (−71 to 172)^g^
			Low birthweight	27/33	0.82 (0.51–1.31)^i^
Meta-analysis of low birthweight (*n* = 7)			Fixed effect		1.01 (0.78–1.31)
			Random effects		0.81 (0.48–1.39)
			Heterogeneity *p*		*p* = 0.01
Weighted analysis of birthweight difference (*n* = 5)			Mean		142 (−53 to 336)
			Heterogeneity *p*		*p* = 0.11

^a^ *CAF* critical assessment form. CAF 4 is a prospective study, CAF 8 is a cross-sectional study, and the rest are clinical trials

^b^ *CBT* cognitive behavioural therapy

^c^Exposed/unexposed cases. *NA* data not available. ‘–’ not applicable

^d^For differences in birthweight the mean is shown (exceptionally in one study exposure per week of NRT use), and for low birthweight the odds ratio (OR) or relative risk (RR) as indicated

^e^Birthweight was noted not to vary significantly by type of NRT used. Also using the Danish National Birth Cohort, Zhu et al. (2014) (CAF 7) reported a clear reduction in birthweight associated with maternal smoking, but no significant reduction associated with NRT use, regardless of whether the father smoked

^f^Adjusted OR in smokers estimated from data provided

^g^Unadjusted mean difference

^h^Unadjusted mean difference estimated from data provided

^i^Unadjusted RR estimated from data provided

^j^OR adjusted for recruitment centre

*Gestational age* (Table 5): Of five clinical trials reporting results for gestational age difference, two (El-Mohandes et al. 2013; Oncken et al. 2008) reported a significant increase associated with NRT allocation, the others no effect. The combined weighted estimate was 0.1 (95 % CI −0.5 to 0.6) weeks. Pollak et al. (2007) also reported results for small for gestational age, but based on very few cases.

**Table 5:** Results summary for NRT and reproduction/development—gestational age/small for gestational age

CAF no.^a^	References	Comparison^b^	Endpoint	Statistic (95 % CI)^c^
13	Pollak et al. (2007)	Any NRT + CBT versus CBT only	Diff. in gestational age (wks)	−0.7 (–1.6 to 0.2)^d^
			Small for gestational age	4.64 (0.25–84.7)^e^
14	Oncken et al. (2008)	Nicotine versus placebo gum	Diff. in gestational age (wks)	0.9 (0.1–1.7)^d^
16	Coleman et al. (2012b)	Nicotine versus placebo patch	Diff. in gestational age (wks)	0.0 (−0.2 to 0.3)^f^
17	El-Mohandes et al. (2013)	Nicotine patch + CBT versus CBT only	Diff. in gestational age (wks)	1.0 (0.16 to 1.84)^d^
18	Berlin et al. (2014)	Nicotine versus placebo patch	Diff. in gestational age (wks)	−0.22 (−0.82 to 0.38)^f^
Weighted analysis of difference in gestational age (wks)			Mean	0.1 (−0.5 to 0.6)
			Heterogeneity *p*	*p* = 0.34

^a^ *CAF* critical assessment form. All studies are clinical trials

^b^ *CBT* cognitive behavioural therapy

^c^For difference in gestational age the mean difference is shown, and for small for gestational age the relative risk (RR)

^d^Unadjusted mean difference estimated from data provided

^e^Unadjusted RR estimated from data provided; based on 4 exposed and 0 unexposed cases

^f^Unadjusted mean difference

*Head circumference and infant length at birth* (Table 6): No significant differences were seen in the two trials reporting results.

**Table 6:** Results summary for NRT and reproduction/development—head circumference/length of infant

CAF no.^a^	References	Comparison	Endpoint (cm)	Mean difference (95 % CI)
14	Oncken et al. (2008)	Nicotine versus placebo gum	Infant length	1.0 (−0.1 to 2.1)^b^
			Head circumference	0.5 (−0.1 to 1.1)^b^
18	Berlin et al. (2014)	Nicotine versus placebo patch	Infant length	0.34 (−0.31 to 0.98)^c^
			Head circumference	−0.20 (−0.63 to 0.24)^c^

^a^ *CAF* critical assessment form. Both studies are clinical trials

^b^Unadjusted mean difference estimated from data provided

^c^Unadjusted mean difference

*Preterm birth* (Table 7): In one epidemiological study (Gaither et al. 2009) results reported relative to non-smokers, for smokers who were or were not prescribed NRT in pregnancy have been converted to an OR within smokers. For consistency with other studies, the OR has been recalculated to compare effects of NRT within smokers. While this OR, 1.88 (0.97–3.56) is close to showing a significant increase in risk, no other result from the six clinical trials does so, one (Oncken et al. 2008) giving a significantly reduced OR (0.39, 0.17–0.91). A meta-analysis of the seven estimates showed no association, the random-effect estimate being 0.98 (0.70–1.37).

**Table 7:** Results summary for NRT and reproduction/development—preterm birth

CAF no.^a^	References	Comparison^b^	Cases^c^	OR/RR (95 % CI)^d^
8	Gaither et al. (2009)	Prescribed/recommended NRT versus not	66/156	1.88 (0.97–3.56)^e^
10	Wisborg et al. (2000)	Nicotine versus placebo patch	10/13	0.8 (0.4–1.7)^f^
13	Pollak et al. (2007)	Any NRT + CBT versus CBT only	24/9	1.37 (0.68–2.75)^g^
14	Oncken et al. (2008)	Nicotine versus placebo gum	7/16	0.39 (0.17–0.91)^g^
16	Coleman et al. (2012b)	Nicotine versus placebo patch	40/45	0.90 (0.58–1.41)^h^
17	El-Mohandes et al. (2013)	Nicotine patch + CBT versus CBT only	1/2	0.50 (0.05–5.18)^g^
18	Berlin et al. (2014)	Nicotine versus placebo patch	27/26	1.03 (0.63–1.71)^i^
Meta-analysis (*n* = 7)		Fixed effect		0.99 (0.78–1.26)
		Random effect		0.98 (0.70–1.37)
		Heterogeneity *p*		0.12

^a^ *CAF* critical assessment form. CAF 8 is a cross-sectional study and the rest are clinical trials

^b^ *CBT* cognitive behavioural therapy

^c^Exposed/unexposed cases

^d^ *OR* odds ratio, *RR* relative risk

^e^RR adjusted for age, marital status, education and race/ethnicity; estimated from data provided

^f^Unadjusted RR

^g^Unadjusted RR estimated from data provided

^h^OR adjusted for recruitment centre

^i^Unadjusted OR

*Neonatal interim care admissions* (Table 8): In the four clinical trials reporting findings no significant differences were seen, the overall random-effect estimate being 0.95 (95 % CI 0.61–1.47),

**Table 8:** Results summary for NRT and reproduction/development—neonatal intensive care admissions

CAF no.^a^	References	Comparison^b^	Cases^c^	OR/RR (95 % CI)^d^
13	Pollak et al. (2007)	Any NRT + CBT versus CBT only	15/3	2.57 (0.77–8.51)^e^
14	Oncken et al. (2008)	Nicotine versus placebo gum	7/11	0.57 (0.23–1.41)^e^
16	Coleman et al. (2012b)	Nicotine versus placebo patch	33/35	0.96 (0.58–1.57)^f^
18	Berlin et al. (2014)	Nicotine versus placebo patch	12/14	0.85 (0.40–1.80)^g^
Meta-analysis (*n* = 4)		Fixed effect		0.94 (0.66–1.35)
		Random effects		0.95 (0.61–1.47)
		Heterogeneity *p*		0.27

^a^ *CAF* critical assessment form. All studies are clinical trials

^b^ *CBT* cognitive behavioural therapy

^c^Exposed/unexposed cases

^d^ *OR* odds ratio, *RR* relative risk

^e^Unadjusted RR estimated from data provided

^f^OR adjusted for recruitment centre

^g^Unadjusted OR

*Neonatal death* (Table 9): The extremely limited data from two trials provides no indication of any major effect of NRT.

**Table 9:** Results summary for NRT and reproduction/development—neonatal death

CAF no.^a^	References	Comparison^b^	Cases^c^	OR/RR (95 % CI)^c^
14	Oncken et al. (2008)	Nicotine versus placebo gum	1/2	0.45 (0.04–4.86)^d^
16	Coleman et al. (2012b)	Nicotine versus placebo patch	0/2	0.20 (0.01–4.24)^e^

^a^ *CAF* critical assessment form. Both studies are clinical trials

^b^Exposed/unexposed cases

^c^ *OR* odds ratio, *RR* relative risk

^d^Unadjusted RR estimated from data provided

^e^Unadjusted OR estimated from data provided. This study also reported results for post-natal deaths, where based on one case in the NRT group, and one in the placebo, an unadjusted RR of 3.07 (0.12–75.4) can be estimated

*Congenital abnormalities* (Table 10): The two clinical trials (Berlin et al. 2014; Coleman et al. 2012b) reported results only for any congenital abnormality, each finding a non-significantly reduced risk based on quite few cases. An analysis based on the Danish National Birth Cohort (Morales-Suarez-Varela et al. 2006) was unusual in only considering NRT users who did not smoke, not mentioning those who smoked and used NRT in pregnancy. Compared with those who neither smoked nor used NRT, the authors reported a marginally significant increased overall risk, with the adjusted relative rate given as 1.61 (95 % CI 1.01–2.58), which they suggested “needs to be replicated”. The RRs listed in Table 10 are based on comparison with smokers using NRT, this being non-significant 1.50 (0.94–2.41). A much larger UK study (Dhalwani et al. 2015) only reported results for major congenital abnormalities, but found no association with overall risk. Combining the estimate for this study with those for any congenital abnormality in the other studies gave a random-effect estimate of 1.10 (0.86–1.41), which reduced to 1.02 (0.81–1.28) if the result for non-smokers from the Danish cohort was excluded. Results by type of abnormality were only available from the two epidemiological studies. No significant increase was seen for major musculoskeletal abnormalities in the Danish cohort, but the UK study reported a significant increase in respiratory system abnormalities (HR 3.49, 95 % CI 1.40–8.71) though no significant effect of NRT for 10 other groupings. Commenting on the respiratory system finding, the authors noted “the statistical power was limited” and “higher morbidities in those women prescribed NRT may also be an explanatory factor”.

**Table 10:** Results summary for NRT and reproduction/development—congenital abnormalities

CAF no.^a^	References	Comparison	Endpoint^b^	Cases^c^	RR/OR (95 % CI)^d^
2	Morales-Suarez-Varela et al. (2006)	Any NRT, no smoking versus smoking no NRT	Any	19/3590	1.50 (0.94–2.41)^e^
			Major	11/2890	1.02 (0.55–1.86)^e^
			MMS	6/884	1.96 (0.86–4.45)^e^
9	Dhalwani et al. (2015)	Prescribed versus not prescribed NRT	Major	90/314	1.07 (0.84–1.36)^f^
			Respiratory system	10/10	3.49 (1.40–8.71)^f^
16	Coleman et al. (2012b)	Nicotine versus placebo patch	Any	9/13	0.70 (0.30–1.66)^g^
18	Berlin et al. (2014)	Nicotine versus placebo patch	Any	4/6	0.67 (0.19–2.33)^h^
Meta-analyses for congenital abnormalities^i^ (*n* = 4)		Fixed effect			1.10 (0.90–1.35)
		Random effects			1.10 (0.86–1.41)
		Heterogeneity *p*			0.34
Excluding CAF 2^j^ (*n* = 3)		Fixed effect			1.02 (0.81–1.28)
		Random effects			1.02 (0.81–1.28)
		Heterogeneity *p*			0.52

^a^ *CAF* critical assessment form. CAF 2 and CAF 9 are prospective studies and the rest are clinical trials

^b^ *MMS* major musculoskeletal

^c^Exposed/unexposed cases

^d^ *OR* odds ratio, *RR* relative risk

^e^Unadjusted relative prevalence rate estimated from data provided

^f^ Hazard ratios were presented adjusted for maternal age at conception, Townsend deprivation index score, and maternal diabetes, asthma, mental illness and multiple births. Adjusted results were also presented for 11 other types of major congenital abnormality, but no significant differences were seen between groups

^g^OR adjusted for recruitment centre

^h^Unadjusted RR estimated from data provided

^i^Preferring any to major for CAF 2

^j^As relates to NRT use by non-smokers

*Apgar score* (Table 11): Three clinical trials presented results for this measure of the health of a newborn child. Different metrics were used, but no significant NRT effect was seen.

**Table 11:** Results summary for NRT and reproduction/development—Apgar score

CAF no.^a^	References	Comparison	Measure	Test of difference^b^
14	Oncken et al. (2008)	Nicotine versus placebo gum	1-min median	*p* = 0.62^c^
			5-min median	*p* = 0.061^c^
16	Coleman et al. (2012b)	Nicotine versus placebo patch	At 5 min <7	0.91 (0.45–1.80)^d^
18	Berlin et al. (2014)	Nicotine versus placebo patch	At 5 min <10	1.18 (0.54–2.57)^e^

^a^ *CAF* critical assessment form. All studies are clinical trials

^b^ *OR* odds ratio, *RR* relative risk

^c^The medians were identical in both the nicotine gum and placebo groups, for both the 1-min score (8) and the 5-min score (9). The *p* value derives from a Mann–Whitney U test

^d^OR adjusted for recruitment centre

^e^Unadjusted RR estimated from data provided

*Other endpoints for NRT and reproduction/development* (Table 12): No significant relationships or consistent patterns with NRT use were evident from the 10 publications providing results.

**Table 12:** Results summary for NRT and reproduction/development—other endpoints

CAF no.^a^	References	Comparison^b^	Endpoint^c^	Cases^d^	OR/RR (95 %CI)^e^
5	Torp-Pedersen et al. (2010)	Any NRT versus no NRT	Strabismus	61/1239	1.22 (0.92–1.61)^f^
6	Milidou et al. (2012)	Any NRT versus no NRT	Infantile colic	137/1417	1.15 (0.97–1.37)^g^
7	Zhu et al. (2014)	Any NRT versus no NRT	ADHD	29/532	1.11 (0.75–1.63)^h^
			HIS	NA	−0.08 (−0.28 to 0.12)^i^
11	Kapur et al. (2001)	Nicotine versus placebo patch	Rapid fetal movements	0/1	0.24 (0.01–5.38)^j^
12	Schroeder et al. (2002)	Nicotine patch	Severe infant morbidity	3/−	No comparison group
13	Pollak et al. (2007)	Any NRT + CBT versus CBT only	Any serious AE	34/10	1.75 (0.93–3.28)^j^
			Placental abnormality	4/0	4.64 (0.25–84.7)^j^
14	Oncken et al. (2008)	Nicotine versus placebo gum	Maternal hospitalization	9/8	1.01 (0.41–2.50)^j^
			Spontaneous abortion	2/0	4.49 (0.22–92.19)^j^
			Any serious AE	24/33	0.65 (0.42–1.01)^j^
16	Coleman et al. (2012b)	Nicotine versus placebo patch	Admission for pregnancy complication	44/41	1.10 (0.70–1.71)^j^
			Any serious AE	9/6	1.53 (0.54–4.34)^j^
18	Berlin et al. (2014)	Nicotine versus placebo patch	Serious AE**—**mother	24/16	1.47 (0.81–2.68)^j^
			Serious AE**—**fetus/newborn	13/14	0.93 (0.45–1.92)^j^
20	Cooper et al. (2014b)	Nicotine versus placebo patch	Infant death	2/2	1.00 (0.14–7.10)^k^
			Definite development impairment	48/64	0.71 (0.48–1.06)^l^
			Survival with no impairment	323/290	1.41 (1.05–1.87)^l^
			Respiratory problems	132/111	1.28 (0.95–1.73)^l^

^a^ *CAF* critical assessment form. CAFs 5 to 7 are prospective studies, and the rest are clinical trials, all controlled except for CAF 12

^b^ *CBT* cognitive behavioural therapy

^c^ *AE* adverse event, *ADHD* attention-deficit/hyperactivity disorder, *HIS* hypersensitivity inattention score. For most studies, the endpoint was recorded during or shortly after pregnancy. Exceptionally, CAF 5 collected data up to March 2006 and CAF 7 data up to August 2011 on births from 1996 to 2003, while CAF 20 followed births for 2 years

^d^Exposed/unexposed cases

^e^ *OR* odds ratio, *RR* relative risk

^f^RR adjusted for year of birth, social class, maternal age at birth, maternal smoking dose, and maternal coffee and tea consumption. No significant relationships were seen by type of strabismus

^g^OR adjusted for maternal age, parity, coffee consumption, alcohol consumption, binge drinking, and education/occupational status of couple. Estimated from data provided, restricted to smokers

^h^HR adjusted for maternal age, parity, alcohol intake during pregnancy, parental socio-economic status, parental psychopathology, and sex of child. Estimated from data provided, restricted to smokers

^i^Difference in mean HIS score for NRT use in smokers estimated from data provided. Adjusted for same variables as for ADHD (see footnote^h^)

^j^Unadjusted RR estimated from data provided

^k^Unadjusted OR estimated from data provided

^l^OR adjusted for recruitment centre

Authors’ conclusions varied as to whether NRT was harmful, beneficial or had no effect on reproduction and development, though views expressed were often far from confident. Possible beneficial effects were concluded for three trials (El-Mohandes et al. 2013; Oncken et al. 2008; Wisborg et al. 2000) based on increases in birthweight and/or gestational age in the NRT group. The large SNAP trial (Cooper et al. 2014b) also noted a beneficial effect on development at 2 years. Possible adverse effects were also noted, with the endpoints varying: overall congenital abnormalities (Morales-Suarez-Varela et al. 2006); infantile colic (Milidou et al. 2012); attention-deficit/hyperactivity disorder (Zhu et al. 2014); low birthweight and preterm birth (Gaither et al. 2009); respiratory system abnormalities (Dhalwani et al. 2015); rapid fetal movements (Kapur et al. 2001); and negative birth outcomes (Pollak et al. 2007). However, four of these conclusions (Gaither et al. 2009; Milidou et al. 2012; Morales-Suarez-Varela et al. 2006; Zhu et al. 2014) were based on significant differences from non-smokers, no longer significant when comparisons were made within smokers. Furthermore, one conclusion (Kapur et al. 2001) was based on a single case of rapid fetal movements in a subject given a placebo patch, while differences noted in the final study (Pollak et al. 2007) were not significant. The only study reporting significantly worse outcomes in smokers using (or allocated to) NRT than in other smokers was the increased incidence of major respiratory system congenital abnormalities (Dhalwani et al. 2015) and even here the authors noted “women prescribed NRT were considerably more likely to have diagnosed morbidities, particularly asthma and mental illnesses”. The remaining studies reported finding no effect, some for specific effects and some for a range of effects.

Except possibly for congenital abnormalities of the respiratory system (Dhalwani et al. 2015), the results in Tables 3, 4, 5, 6, 7, 8, 9, 10, 11 and 12 show no evidence of a significantly poorer outcome in NRT users, whether in individual studies or in meta-analyses. Indeed, there is some evidence that allocation to NRT is associated with increased birthweight and gestational age at birth, and better development of their offspring at 2 years. However, despite the quite large number of studies, the evidence has various limitations, including the few NRT exposed cases for some endpoints, the lack of dose–response data on dose or duration of use, the minimal data by type of NRT used, and the failure to adjust for extent and duration of cigarette smoking before NRT prescription, or extent of change in smoking following it.

For some more commonly studied conditions (fetal loss/spontaneous abortion, birthweight/low birthweight, gestational age/small for gestational age, preterm birth, neonatal intensive care admissions and overall incidence of congenital abnormalities), where a lack of significant association was seen in meta-analyses, the data provide limited evidence suggesting a lack of effect of NRT. Limited evidence of an effect is more appropriate for congenital abnormalities of the respiratory system, where the observed positive association may result from confounding by increased morbidity in pregnant smokers allocated to NRT. For other endpoints considered (head circumference/length of infant, neonatal death and Apgar score), an assessment of inadequate evidence seems more appropriate for NRT.

### Cardiovascular disease

The cardiovascular effects commonly observed with acute NRT use are an increase in heart rate, blood pressure and cardiac output (Benowitz and Gourlay 1997). These effects are physiological responses to nicotine’s activation of nAChRs, resulting in part from sympathetic neural stimulation and catecholamine release from the adrenal glands (Benowitz and Gourlay 1997; Klevans and Gebber 1970). Because of the rapid development of tolerance to these physiological alterations, it seems unlikely that these acute effects play a significant role in cardiovascular disease (SAHE). In support of this statement, long-term (18–24 months) nicotine exposure (at levels observed in NRT users) does not appear to cause cardiovascular disease in experimental animals (Theophilus et al. 2012; Waldum et al. 1996; Wilson et al. 1938). In contrast, the short-term (≤12 weeks) administration of nicotine to animals has been shown to enhance or aggravate existing cardiovascular disease (i.e. chronic hypertension, atherosclerosis, myocardial ischaemia/reperfusion injury) in experimental models (Bui et al. 1995; Lau et al. 2006; Sridharan et al. 1985; Zhou et al. 2013). The results of these non-clinical studies suggest the potential for serious cardiovascular adverse effects with nicotine exposure and support the need for a comprehensive review of studies investigating the presence of such SAHE in NRT users.

As summarized in Supplementary File 4, six epidemiological studies (CAFs 21 to 26) provided relevant information. One was a case–control study of MI (Kimmel et al. 2001), one a case series analysis (Hubbard et al. 2005), one a prospective study of HIV-infected individuals (Elzi et al. 2006) and three prospective studies of patients undergoing cardiac procedures (Meine et al. 2005; Paciullo et al. 2009; Woolf et al. 2012). Two studies (CAFs 24 and 25) were considered of “poor” study quality, the other four “fair”. Common weaknesses of these studies include few relevant events, short follow-up period and lack of control for confounders, including changes in smoking habits after starting NRT. Four clinical trials of patients with cardiac disease also provided relevant data (CAFs 27, 28A, 29 and 30A). Three trials (Joseph et al. 1996; Rennard et al. 1994; Tzivoni et al. 1998) were double-blind RCTs of nicotine patches, ascribed a “low” risk of bias. The other (Mohiuddin et al. 2007), ascribed a “high” risk of bias, was less informative, only providing results relevant to the effects of an intensive intervention with individualized pharmacotherapy, which only included NRT for some patients, results not being separately presented for those prescribed NRT. Note that one further clinical trial (Thomsen et al. 2010) reported two cases of cardiovascular complications in those allocated to an intervention group including free supply of NRT and one in the control group, as part of a range of complications considered. Including those results, considered in the section on “Stroke”, would not have affected the conclusions for CVD. The studies provide results for various endpoints, as considered below.

*AMI* (Table 13): Four studies (Joseph et al. 1996; Kimmel et al. 2001; Mohiuddin et al. 2007; Woolf et al. 2012), each based on less than 10 exposed cases, gave RRs (or ORs) less than 1.00, but not significantly so. Another study (Elzi et al. 2006), also on few cases, gave a significantly increased RR of 5.63 (95 % CI 1.07–29.64). This was based on an uncontrolled comparison of HIV patients participating (and not participating) in a smoking cessation program involving supply of NRT, data being presented showing a substantial increase in history of CHD at baseline in the NRT exposed group. An analysis of a UK national database (Hubbard et al. 2005) based on 33,247 individuals prescribed NRT compared AMI incidence in the 56 days pre- and post-prescription with that in the period more than 56 days from the prescription. The high RR of 5.55 (95 % CI 4.42–6.98) in the 56 days before NRT prescription suggests reverse causation, with NRT “being prescribed shortly after myocardial infarctions and strokes”, and is irrelevant to whether NRT might cause AMI. The lower RR of 1.27 (0.82–1.97) for the 56 days after prescription (which did not vary significantly by type of NRT) is more relevant. Excluding the high RR, the remaining estimates give a combined random-effect meta-analysis estimate of 0.97 (0.55–1.71). Also omitting two studies not of NRT *per se* (Elzi et al. 2006; Mohiuddin et al. 2007), the estimate rises to 1.08 (0.75–1.56), but remains non-significant.

**Table 13:** Results summary for NRT and CVD–AMI

CAF no.^a^	References	Comparison	Endpoint	Cases^b^	RR/OR (95 % CI)
21	Kimmel et al. (2001)	Used nicotine patch in week before MI versus did not use	MI	3/650	0.46 (0.09–1.47)^c,d^
22A	Hubbard et al. (2005)	56 days before versus >56 days outside NRT prescription	MI	88/741	5.55 (4.42–6.98)^d,e^
		56 days after versus >56 days outside NRT prescription		21/741	1.27 (0.82–1.97)^d,f^
24	Elzi et al. (2006)	Provided NRT versus not	MI within 1 year	2/4	5.63 (1.07–29.64)^g^
26	Woolf et al. (2012)	Prescribed NRT versus not	MI within 1 year	8/23	0.90 (0.40–2.06)^d^
28A	Joseph et al. (1996)	Nicotine versus placebo patch	MI or cardiac arrest within 14 weeks	1/2	0.49 (0.04–5.41)^g,h^
30A	Mohiuddin et al. (2007)	Intervention^i^ versus usual care	MI admission within 2 years	9/17	0.49 (0.23–1.04)^g,h^
Meta-analysis^i^ (*n* = 6)		Fixed effects			0.99 (0.72–1.38)
		Random effects			0.97 (0.55–1.71)
		Heterogeneity *p*			0.07
Meta-analysis^j^ omitting Elzi et al. (2006) and Mohiuddin et al. (2007) (*n* = 4)		Fixed effects			1.08 (0.75–1.56)
		Random effects			1.08 (0.75–1.56)
		Heterogeneity *p*			0.47

Note: no cases of AMI were reported in the clinical trials summarized in CAFs 27 and 29

^a^ *CAF* clinical assessment form. CAF 21 is a case–control study, CAF 22A is a case series analysis, CAF 24 is a prospective study of individuals with HIV, CAF 26 is a prospective study of patients undergoing procedures for CVD, and CAFs 28A and 30A are clinical trials in patients with CVD

^b^Exposed/unexposed cases

^c^See CAF 21 for further ORs (all <1) according to smoking at time of patch use, confirmed (rather than any) AMI, timing of patch use and NRT use (rather than patch use)

^d^Adjusted estimate**—**see CAF for details of adjustment variables

^e^Relative incidence in the 56 days before the NRT prescription

^f^Relative incidence in the 56 days after the NRT prescription. There was no evidence of an increase in incidence for any formulation of NRT

^g^Unadjusted estimate

^h^Estimated from data provided

^i^Includes NRT prescription for some subjects

^j^Omitting first estimate from Hubbard et al. (2005)

*Mortality* (Table 14): For the studies providing relevant results, the period following exposure varied, where stated, from 56 days to 2 years. Since the deaths were shortly after NRT exposure, and in all studies except one (Hubbard et al. 2005) in patients with cardiac disease, most deaths were probably from CVD, though only Mohiuddin et al. (2007) give mortality results specifically for CVD. Although deaths in exposed cases were generally few, only exceeding 10 in the case series analysis (Hubbard et al. 2005), the RRs were, with one exception, below 1.00, though only the RR of 0.23 (95 % CI 0.07–0.73) for the study of intensive intervention versus usual care (Mohiuddin et al. 2007) was significant at *p* < 0.05. The exception was the study of patients undergoing coronary artery bypass graft surgery (Paciullo et al. 2009), where the adjusted RR, estimated as 6.06 (1.65–22.21), was based on only three exposed cases (of 40 at risk) and one unexposed case (of 489). Note that the unadjusted RR of 2.47 (0.74–8.32) was not significant, the reliability of adjusted analyses based on so few deaths being open to question. Random-effect meta-analysis showed no significant overall effect, the RR being 0.81 (95 % CI 0.41–1.60) based on all the studies and 1.01 (0.51–1.98) omitting the trial of intensive intervention (Mohiuddin et al. 2007). Both analyses showed significant (*p* < 0.05) heterogeneity due to the single high estimate.

**Table 14:** Results summary for NRT and CVD—Mortality

CAF no.^a^	References	Comparison	Endpoint	Cases^b^	RR (95 % CI)
22A	Hubbard et al. (2005)	56 days before versus >56 days outside NRT prescription	Mortality	33/−	0.86 (0.60–1.23)^c^
		56 days after versus >56 days outside NRT prescription			
23	Meine et al. (2005)	Prescribed nicotine patch versus not	1-year mortality^d^	9/10	0.90 (0.37–2.16)^e,f^
24	Elzi et al. (2006)	Provided NRT versus not	1-year CVD mortality^g^	0/1	Not estimated^h^
25	Paciullo et al. (2009)	Used nicotine patch versus not	Mortality^i^	3/1	6.06 (1.65–22.21)^c,j^
26	Woolf et al. (2012)	Prescribed NRT versus not	1-year mortality	7/24	0.80 (0.33–1.91)^c^
28A	Joseph et al. (1996)	Nicotine versus placebo patch	14-week mortality	1/6	0.16 (0.02–1.36)^e,f^
30A	Mohiuddin et al. (2007)	Intervention^k^ versus usual care	2-year mortality		
			All causes	3/12	0.23 (0.07–0.73)^c^
			CVD	3/9	0.31 (0.09–1.10)^e,f^
Meta-analysis^l^		Fixed effect			0.84 (0.63–1.13)
		Random effects			0.81 (0.41–1.60)
		Heterogeneity *p*			0.007
Meta-analysis omitting Mohiuddin et al. (2007)		Fixed effect			0.92 (0.68–1.24)
		Random effects			1.01 (0.51–1.98)
		Heterogeneity *p*			0.027

Note: no deaths were reported in the clinical trials summarized in CAFs 27 and 29

^a^ *CAF* clinical assessment form. CAF 22A is a case series analysis, CAFs 23, 25 and 26 are prospective studies of patients undergoing procedures for CVD, CAF 24 is a prospective study of individuals with HIV and CAFs 28A and 30A are clinical trials in patients with CVD

^b^Exposed versus unexposed cases

^c^Adjusted estimate**—**see CAF for details of adjustment variables

^d^Mortality rates were also non-significantly lower for patch for 7-day and 30-day mortality

^e^Estimated from data provided

^f^Unadjusted estimate

^g^Deaths due to lung cancer or HIV-related not considered

^h^No reliable OR estimate could be made, the comparison being of 0/34 with 1/383

^i^Follow-up period not stated

^j^Compared to smokers not prescribed NRT; the RR versus non-smokers being 6.17 (1.62–23.69)

^k^Includes NRT prescription for some subjects

^l^Using all cause estimate from Mohiuddin et al. (2007)

*Admissions/readmissions* (Table 15): Six studies presented results for admissions/readmissions overall or for various heart-related reasons. Numbers of exposed cases tend to be greater than in Tables 13 and 14. Of 15 estimates, five are above 1.00 and 10 below. The only two significant at *p* < 0.05 were decreases seen in the trial of intensive intervention (Mohiuddin et al. 2007). Due to the variety of endpoints, and the few estimates for any, meta-analysis has not been conducted.

**Table 15:** Results summary for NRT and CVD—Admissions/readmissions

CAF no.^a^	References	Comparison	Endpoint^b^	Cases^c^	RR (95 % CI)
23	Meine et al. (2005)	Prescribed nicotine patch	Coronary bypass	37/26	1.42 (0.90–2.25)^d^
			Coronary angioplasty	94/79	1.19 (0.95–1.48)^d^
26	Woolf et al. (2012)	Prescribed NRT versus not	Repeat revascularization	18/58	0.77 (0.44–1.36)^e^
			Hospitalization^f^	41/104	1.01 (0.66–1.53)^e^
27	Rennard et al. (1994)	Prescribed nicotine patch versus not	Hospitalization	0/2^f^	0.21 (0.01–4.20)^d,h^
28A	Joseph et al. (1996)	Nicotine versus placebo patch	Increased severity of angina	7/10	0.69 (0.27–1.79)^d,h^
			Arrhythmia	5/3	1.64 (0.40–6.82)^d,h^
			Congestive heart failure	2/2	0.99 (0.14–6.96)^d,h^
			Outpatient visit for increased severity of atherosclerotic CVD	12/7	1.69 (0.68–4.23)^d,h^
29	Tzivoni et al. (1998)	Nicotine versus placebo patch	Bypass surgery	0/1	0.35 (0.01–8.31)^d,h^
30A	Mohiuddin et al. (2007)	Intervention^h^ versus usual care	Admission for:		
			Any cause^i^	25/41	0.56 (0.37–0.84)^d^
			CVD	20/37	0.50 (0.31–0.79)^d,h^
			Unstable angina	8/14	0.52 (0.23–1.20)^d,h^
			Cardiac arrhythmia	1/2	0.46 (0.04–4.98)^d,h^
			Decompensated heart failure	2/4	0.46 (0.09–2.45)^d,h^

^a^ *CAF* clinical assessment form. CAFs 23, 25 and 26 are prospective studies of patients undergoing procedures for CVD, CAF 24 is a prospective study of individuals with HIV, and the rest clinical trials in patients with CVD

^b^Follow-up periods were CAFs 23 and 26 1 year, CAF 27 5 weeks, CAF 28A 14 weeks, CAF 29 2 weeks and CAF 30A 2 years

^c^Exposed/unexposed cases

^d^Estimated from data provided

^e^Adjusted estimate**—**see CAF for details of adjustment variables

^f^For angina, congestive heart failure, or arrhythmia

^g^One for chest pain resulting in bypass surgery, one for non-CVD reasons attributed to nicotine withdrawal

^h^Unadjusted

^i^Includes NRT prescription for some subjects

^j^Including AMI

*Other endpoints* (Table 16): Two clinical trials presented relevant results, though numbers of exposed cases are often quite low. Of the six effect estimates, one is above and five below 1.00, with none close to statistically significant (at *p* < 0.05). In contrast, the study of HIV-infected individuals (Elzi et al. 2006) gave an RR of 7.51 (95 % CI 1.30–43.41) for undergoing coronary angioplasty. However, as for AMI, the analysis took no account of the much higher baseline history of CVD in the group given NRT.

**Table 16:** Results summary for NRT and other CVD endpoints

CAF no.^a^	References	Comparison	Endpoint^b^	Cases^c^	RR/OR (95 % CI)^d^
24	Elzi et al. (2006)	Provided NRT versus not	Underwent percutaneous transluminal coronary angioplasty	2/3	7.51 (1.30–43.41)
27	Rennard et al. (1994)	Provided nicotine patch versus not	Premature termination due to adverse events^e^	3/8	0.38 (0.11–1.40)
			Angina at week 1	8/13	0.63 (0.28–1.44)
			Angina at week 5^f^	5/7	0.73 (0.24–2.21)
			Clinically important ECG changes^g^	22/26	0.87 (0.54–1.39)
29	Tzivoni et al. (1998)	Nicotine versus placebo patch	Serious adverse experiences^h^	2/1	2.08 (0.19–22.22)
			Ischaemic episodes^i^	17/25	0.71 (0.44–1.15)

^a^ *CAF* clinical assessment form. CAF 24 is a prospective study of individuals with HIV and the other two clinical trials in patients with CVD

^b^Follow-up periods were CAF 24 1 year, CAF 27 5 weeks and CAF 29 2 weeks

^c^Exposed/unexposed cases

^d^All estimates were unadjusted and estimated from data provided

^e^Of various types, mainly age related, none significantly increased in the exposed group

^f^Data at weeks 2, 3 and 4 also showed no significant difference

^g^Of various types, none significantly increased in the exposed group

^h^All relating to angina

^i^No significant treatment differences were seen in relation to timing or total number of episodes

Conclusions reached by the authors on NRT and CVD were generally not specific to an endpoint. Two (El-Mohandes et al. 2013; Kimmel et al. 2001) were quite positive that NRT (or intensive intervention) had a beneficial effect, while most authors simply argued no effect was demonstrated. The exception was Paciullo et al. (2009), who noted the significant increase in mortality, but commented that “additional evaluation in large patient cohorts with prospective controls is warranted”.

Except possibly for findings from two “poor” quality studies (Elzi et al. 2006; Paciullo et al. 2009), based on few exposed cases, the overall evidence suggests NRT is not associated with any increased CVD risk. However, it cannot be regarded as conclusive of a lack of effect. Limitations of the evidence include small numbers of exposed cases in most studies, some analyses not relating to actual use of NRT, the short follow-up period, the absence of dose–response data, and the failure to adjust for smoking either before or after NRT prescription. We conclude there is limited evidence suggesting a lack of effect concerning the relationship of NRT use to the risk of CVD.

### Stroke

NRT product warning label statements indicate a potential health risk to NRT users with high blood pressure (US Food and Drug Administration 2015a). Such warnings suggest a potential risk of stroke with NRT or nicotine exposure. In fact, several animal studies support a potential role for nicotine in increasing cerebral microvessel thrombosis (Fahim et al. 2014) and inhibiting restoration of brain microvascular flow (Wang et al. 1997) in experimental models of thromboembolic injury and repair (as compared to vehicle controls without nicotine). Three published studies from separate laboratories (Bradford et al. 2011; Paulson et al. 2010; Wang et al. 1997) also investigated the impact of nicotine exposure on cerebral ischaemia/reperfusion injury in experimental animal models. Similar to the findings observed for myocardial ischaemia/reperfusion injury, short-term nicotine exposure increased cerebral infarct size. Based on findings from non-clinical studies described above, we evaluated epidemiological studies and clinical trials for an association between stroke and NRT use.

Only three studies (Hubbard et al. 2005; Joseph et al. 1996; Panos et al. 2010) provide information on NRT and stroke, fuller details being presented in CAFs 22B, 28B and 31. The first (Hubbard et al. (2005), study quality “fair”) was the case series analysis which also reported results on CVD and suffers from weaknesses noted above. The second (Joseph et al. (1996), risk of bias “low”) was the clinical study of patients with cardiac disease also reporting results for CVD and other SAHEs. The third (Panos et al. (2010), study quality “fair”) was a prospective study of patients admitted to an intensive care unit with a neurological insult. Note that one study (Carandang et al. 2011) which reported on the relationship between patch use and various adverse health effects in subarachnoid haemorrhage patients is considered in the section below on “Other SAHEs in patients”.

Table 17 summarizes the main results. The high RR (compared to baseline levels) of 3.59 (95 % CI 2.56–5.03) in the 56 days before NRT prescription for the case series analysis (Hubbard et al. 2005) suggests reverse causation and is irrelevant to whether NRT can cause strokes. The lower RR of 1.30 (0.77–2.19) for the 56 days after prescription is more relevant. There was no evidence of an effect of NRT in the 56 days post-prescription in subgroups by sex, age, previous history of angina or hypertension, or by type of NRT used, nor when analyses were repeated using second stroke as the outcome. An RR of 2.47 (1.16–5.24) in days 43–56 after prescription was regarded by the authors as an “isolated increase”, no evidence of an increased risk being seen for days 1–14, 15–28 or 29–42. No evidence of an effect of NRT was seen in the small clinical trial (Joseph et al. 1996) or in the study of neurological patients (Panos et al. 2010). None of the authors of the three publications claimed any adverse effect on stroke had been established.

**Table 17:** Results summary for NRT and stroke

CAF no.^a^	References	Comparison	Endpoint	Cases^b^	RR/OR (95 % CI)
22B	Hubbard et al. (2005)	56 days before versus >56 days outside NRT prescription	Stroke	39/444	3.59 (2.56–5.03)^c,d^
		56 days after versus >56 days outside NRT prescription		15/444	1.30 (0.77–2.19)^c,e^
31	Panos et al. (2010)	Given NRT versus not given	Ischaemic stroke^f^	3/6	0.50 (0.13–1.93)^g^
			Death^f,h^	5/6	0.83 (0.26–2.63)^g^
			Vasospasm^f^	23/12	1.90 (0.99–3.63)^g^
			Unfavourable discharge^f^	48/37	1.29 (0.91–1.87)^g^
28B	Joseph et al. (1996)	Nicotine versus placebo patch	Admission within 14 weeks for cerebrovascular disease	4/3	1.32 (0.30–5.82)^g,i^

^a^ *CAF* clinical assessment form. CAF 22B is a case series analysis, CAF 31 is a prospective study of patients undergoing neurological procedures and CAF 28B is a clinical trial in patients with CVD

^b^Exposed/unexposed cases

^c^Adjusted estimate**—**see CAF for details of adjustment variables

^d^Relative incidence in the 56 days before the NRT prescription

^e^Relative incidence in the 56 days after the NRT prescription. There was no evidence of an increase in incidence for any formulation of NRT

^f^Follow-up period unclear

^g^Unadjusted

^h^As the study followed up patients admitted to an intensive care unit for a neurological insult, many of these are likely to be stroke-related

^i^The authors noted that the association was considerably weakened after adjustment for subarachnoid haemorrhage at baseline

While the results provide no clear evidence of any increased risk of stroke, one cannot regard the findings as conclusive of a lack of effect. This is because of the few publications and cases of stroke post-NRT and various study weaknesses. We consider there is inadequate evidence concerning the relationship of NRT to stroke.

### Other SAHEs in patients

NRT product warning label statements (health-related) indicate potential risk to users with gastrointestinal (GI) conditions (such as stomach ulcers) and diabetes. For diabetes, several human studies demonstrate nicotine exposure results in enhanced insulin resistance in type 2 diabetics but not in healthy individuals (Axelsson et al. 2001; Epifano et al. 1992). For example, a study from Axelsson and co-workers investigated the effects of acute intravenous nicotine infusion on insulin sensitivity in non-smoker type 2 diabetics versus healthy non-smoker controls. While subjects with type 2 diabetes were more insulin resistant than healthy controls at baseline, nicotine infusion significantly decreased insulin sensitivity in type 2 diabetics but did not modify the insulin response in controls (Axelsson et al. 2001). In addition to diabetes, studies using well-known experimental models for peptic ulcer formation, pancreatic injury and renal injury have also demonstrated that short-term nicotine exposure has the potential to aggravate all of these pathological conditions (Arany et al. 2011; Chowdhury et al. 1995; Qiu et al. 1991; Wong et al. 2002). Therefore, based on findings from clinical and non-clinical studies described above, we evaluated epidemiological studies and clinical trials for an association between NRT use and other SAHEs such as diabetes, peptic ulcer, pancreatitis and renal effects.

As summarized in Supplementary File 5, one epidemiological study (Carandang et al. 2011) and four clinical trials (Joseph et al. 1996; Lee et al. 2013; Mohiuddin et al. 2007; Thomsen et al. 2010) of patients provide information relating NRT and other SAHEs not generally related to cancer or CVD. Further details are presented in CAFs 28C, 30B, 32, 33 and 34. The epidemiological study (Carandang et al. 2011), in patients with subarachnoid haemorrhage, was ascribed study quality “fair”. Three clinical trials (Lee et al. 2013; Mohiuddin et al. 2007; Thomsen et al. 2010) were ascribed a “high” risk of bias for only providing results for an intervention which included use of NRT (not necessarily in all patients), the results possibly reflecting possible effects of intervention rather than of NRT. The other trial (Joseph et al. 1996), which did allocate patients to NRT, was ascribed risk of bias “low”. Common weaknesses include the small number of relevant events, the relatively short follow-up period and the failure to control for changes in smoking habits following starting NRT. A variety of endpoints were considered, including complications following surgery and readmissions to hospital.

The main results are summarized in Table 18, some further results being given in the CAFs. The only significant (*p* < 0.05) differences noted in relation to NRT use (or intervention) were for clinical vasospasm and poor outcome in the study of subarachnoid haemorrhage patients (Carandang et al. 2011), which was less frequent (OR 0.46, 95 % CI 0.25–0.84) in patients using patches. Total length of stay in hospital was also significantly shorter in patch users in this study (see CAF 32). There was no significant adverse effect of NRT use (or intervention) for any other endpoint. The authors generally agreed in regarding the results as not demonstrating an adverse effect of NRT for intervention.

**Table 18:** Results summary for studies in patients on NRT and other serious health effects

CAF no.^a^	References	Comparison	Endpoint^b^	Cases^c^	RR/OR (95 % CI)
32	Carandang et al. (2011)	Prescribed nicotine patch versus not	Mortality	2/12	0.36 (0.07–1.43)^d,e^
			Angiographic vasospasm	39/90	0.85 (0.65–1.12)^d,e^
			Clinical vasospasm	17/56	0.45 (0.23–0.88)^f^
			Poor outcome	16/64	0.46 (0.25–0.84)^f^
28C	Joseph et al. (1996)	Nicotine versus placebo patch	Admission for peripheral vascular disease	3/5	0.59 (0.14–2.45) ^d,e^
			Admission for other reasons	16/13	1.21 (0.59–2.48)^d,e^
30B	Mohiuddin et al. (2007)	Intervention^f^ versus usual care	Non-CVD mortality	0/3	Not significant^d^
			Non-cardiac hospitalizations	5/4	1.15 (0.32–4.15)^d,e^
33	Lee et al. (2013)	Intervention^f^ versus usual care	Complications		
			Interoperative	5/6	0.83 (0.27–2.6)^d^
			Post-operative (immediate)	2/5	0.39 (0.08–2.0)^d^
			Any time^h^	11/14	0.79 (0.38–1.63)^d^
			In 30-day follow-up	12/8	1.40 (0.61–3.2)^d^
			Unanticipated hospital admission	2/2	0.98 (0.14–6.8)^d^
			Unscheduled visit during post-operative period	16/9	1.66 (0.78–3.6)^d^
34	Thomsen et al. (2010)	Intervention^f^ versus not	Any wound complication	25/28	0.97 (0.65–1.45)^d^
			Any complication^i^	38/35	1.00 (0.73–1.33)^d^
			Secondary surgery	1/0	Not significant^e^

^a^ *CAF* clinical assessment form. CAF 32 is a prospective study of patients with a subarachnoid haemorrhage, CAFs 28C and 30B are clinical trials in patients with CVD, while CAFs 33 and 34 are clinical trials of surgical patients

^b^Follow-up periods were 14 weeks for CAF 28C and 2 years for CAF 30. For CAFs 32, 33 and 34, the periods were not clearly defined but results mainly relate to the period before, or shortly after, hospital discharge

^c^Exposed/unexposed cases

^d^Unadjusted

^e^Estimated from data provided

^f^Adjusted estimate**—**see CAF for details of adjustment variables

^g^Includes supply of NRT

^h^It was not made clear what period this related to

^i^Also includes respiratory and cardiovascular complications, urinary tract infection, nausea and vomiting and other complications

The available evidence does not indicate any effect of NRT. However, this cannot be regarded as conclusive due to the weaknesses noted above. We conclude there is inadequate evidence concerning the relationship of NRT to the risk of the SAHEs considered here.

### Other SAHEs, mainly in healthy populations

As summarized in Supplementary File 6, four meta-analyses (Greenland et al. 1998; Mills et al. 2010; Moore et al. 2009; Stead et al. 2012) provided relevant results, more details being given in CAFs 35–38. Most studies considered in the meta-analyses were of trials conducted in healthy populations, but some were of patients with specified conditions. Three meta-analyses (Greenland et al. 1998; Moore et al. 2009; Stead et al. 2012) limited attention to RCTs, the other (Mills et al. 2010) also including results of observational studies. One meta-analysis (Moore et al. 2009) was limited to RCTs of smokers declaring no intention to quit, concerned smoking reduction and involved only seven RCTs. The other meta-analyses concerned smoking cessation studies and involved far more studies, the more recent meta-analyses (Mills et al. 2010; Stead et al. 2012) combining data from over 100 studies. For two meta-analyses (Greenland et al. 1998; Mills et al. 2010), adverse events were clearly the central interest. However, one (Moore et al. 2009) was mainly concerned with efficacy and safety, and another (Stead et al. 2012) with smoking cessation, both giving very limited results for adverse events.

The main findings of the four meta-analyses are summarized in Table 19. Where prevalences were only given for the NRT groups, these are noted in CAF 37, but not included in Table 19, nor considered further here. As listed in Table 19, no significant relationships between NRT and any health effect were reported in the earliest meta-analysis (Greenland et al. 1998), based on quite low total incidences, or in the meta-analysis of smoking reduction studies (Moore et al. 2009). However, the recent meta-analyses (Mills et al. 2010; Stead et al. 2012), no doubt based on sets of studies which overlap considerably, both reported a significant (*p* < 0.01) approximate doubling of risk of heart palpitations and chest pains in subjects allocated to NRT. Mills et al. (2010) noted that the increased risk was evident both for patches and orally administered NRT and refer to high nicotine concentrations in the serum of NRT users who continue to smoke, and pre-existing CVD as possible explanations for the association.

**Table 19:** Results of meta-analyses of NRT and other serious adverse health effects

CAF no.^a^	References	Endpoint	Exposed % (*n*)	Unexposed % (*n*)	RR (95 % CI)
35	Greenland et al. (1998)	AMI	0.8 (3)	0.8 (3)	1.00 (0.17–5.83)
		Stroke	0.3 (1)	0.6 (2)	0.54 (0.02–6.73)
		Tachycardia	0.8 (2)	0.0 (0)	Not significant
		Palpitations	0.4 (2)	1.8 (8)	0.26 (0.04–1.10)
		Angina	0.4 (1)	0.4 (1)	1.00 (0.03–39.0)
		Arrhythmia	2.7 (11)	2.2 (9)	1.26 (0.56–2.87)^b^
		Hypertension	2.3 (8)	1.4 (5)	1.60 (0.52–5.48)^c^
		Asthma	0.0 (0)	1.7 (2)	Not significant
		Bronchitis	7.8 (9)	4.2 (5)	1.91 (0.63–6.54)^d^
		Urogenital symptoms	0.0 (0)	0.8 (1)	Not significant
		Neurological symptoms	3.5 (4)	0.6 (1)	3.80 (0.51–10.6)
36	Moore et al. (2009)	Death	0.3 (4)	0.3 (4)	1.00 (0.25–4.02)^e^
		Serious adverse event^f^	7.7 (86)	7.1 (79)	1.09 (0.79–1.50)^e^
		Discontinued because of adverse event	1.7 (19)	1.3 (15)	1.27 (0.64–2.51)^e^
37	Mills et al. (2010)	Death	0.8 (11)	1.2 (16)	0.74 (0.33–1.67)
		Heart palpitations and chest pains	3.0 (189)	1.6 (64)	2.06 (1.51–2.82)^g^
		Serious adverse events	“25 RCTs reported serious adverse events occurring but none were statistically significant (data not shown)”		
38	Stead et al. (2012)	Heart palpitations and chest pains^h^	2.5 (167)	1.4 (62)	1.88 (1.37–2.57)

^a^ *CAF* clinical assessment form

^b^The RR (95 % CI) per 21 mg nicotine was given as 1.43 (0.48–4.24)

^c^The RR (95 % CI) per 21 mg nicotine was given as 1.79 (0.50–6.45)

^d^The RR (95 % CI) per 21 mg nicotine was given as 2.12 (0.62–7.27)

^e^No significant differences in RR were noted by type of NRT used

^f^None judged likely to be due to treatment

^g^Both patches and oral NRT were associated with an increased risk

^h^This was an attempt to replicate the findings of Mills et al. (2010). Otherwise the authors made no systematic attempt to synthesize quantitatively the incidence of the various side effects reported with the different NRT preparations. This was because of the extensive variation in reporting the nature, timing and duration of symptoms

The first two reviews saw no evidence of harm from NRT. However, the third review (Mills et al. 2010) noted that “The use of NRT is associated with a variety of side effects”. However, the side effects referred to, for example, nausea and insomnia are generally not considered serious. Even heart palpitations and chest pains are not generally considered SAHEs (Little and Ebbert 2015; Stead et al. 2012; US Food and Drug Administration 2015a). In agreement, the final review (Stead et al. 2012) noted that “there is no evidence that NRT increases the risk of heart attacks”. Commenting on the results for heart palpitations and chest pains, they stated “this is potentially the only clinically significant serious adverse event to emerge from the trials and constitutes an extremely rare event, occurring at a rate of 2.5 % in the NRT groups compared with 1.4 % in the control groups in the 15 trials in which it was reported at all”.

The third review (Mills et al. 2010) points out various relevant limitations, which apply generally: “These include limitations of the primary studies themselves as well as those associated with combining results across potentially heterogeneous studies or populations. The main limitation of the primary studies is the mechanism by which adverse events are recorded. In the majority of instances this would be through passive reporting and therefore be susceptible to the underreporting associated with such techniques”. Nevertheless, the magnitude and significance of the association of NRT use in RCTs with the reported incidence of heart palpitations and chest pains strongly suggest a causal relationship, though whether this increased risk is limited to patients with pre-existing CVD is unclear from the available evidence. There appears to be sufficient evidence of a relationship between NRT and the incidence of heart palpitations and chest pains, but these outcomes are not considered SAHEs. For SAHEs, however, the data can generally be regarded as limited evidence suggesting a lack of effect of NRT, though for the more rarely seen conditions inadequate evidence seems a more appropriate assessment.

## Discussion

For many endpoints considered, the evidence presented is clearly inadequate to reach a reliable conclusion on whether NRT has an effect. This includes cancer, stroke, the serious health effects in patients considered in Table 18 and some of the less commonly considered reproduction/development endpoints. For some more commonly studied reproduction/development endpoints (including fetal loss, spontaneous abortion, birthweight, prematurity, neonatal intensive care admissions, overall incidence of congenital abnormalities and ADHD) and also for CVD, the evidence available suggests a lack of effect of NRT. Only for two endpoints is there any apparent evidence of harm. One, only providing limited evidence, given the association may result from confounding by higher morbidities in women prescribed NRT, is congenital abnormalities of the respiratory system where an increased incidence associated with NRT was seen in one study (Dhalwani et al. 2015). The other is heart palpitations and chest pains where a significant doubling of risk was reported in two recent meta-analyses of evidence from randomized controlled studies (Mills et al. 2010; Stead et al. 2012), which we regard as sufficient evidence of an effect, though it is not a SAHE.

### Limitations

Numerous limitations affect the available evidence on possible adverse health risks from NRT use. First consider the evidence from the clinical trials. These typically compare a defined group of smokers allocated to receive NRT or a placebo and are designed to investigate effects of allocation on cessation rates. For any given outcome, there are three possible results, a significant increase in risk of a SAHE, a significant decrease in risk, or no significant change.

Interpreting a significant increase in risk seems relatively straightforward. Unless due to chance, to confounding (unlikely to be relevant in an RCT) or to an effect of smoking withdrawal in smokers who quit as a result of using NRT, it provides direct evidence of an effect of NRT.

The finding of no significant difference in risk can have various interpretations. One obvious possibility is the study was too small, particularly likely for relatively rare outcomes, since clinical studies are generally powered to detect effects on cessation, not on health outcomes. A second possibility is that actual usage of NRT was only for a very limited period. A third is that the follow-up period may have been too short. Especially for chronic diseases, risks may, for some time, depend predominantly on past exposure, before allocation to NRT.

The finding of a significant decrease in risk of a SAHE in the NRT group can also have various interpretations and is hindered by the lack of corresponding results for never smokers. Thus, the endpoint may be related to components of smoke other than nicotine, with the observed reduction due to greater quitting in the NRT group. Alternatively, the endpoint may be increased by nicotine exposure, with the reduced risk due to the reduced dose of nicotine, either as a result of increased quitting in the NRT group, or to the NRT delivering less nicotine than cigarettes.

There are also other issues which limit interpretation. For instance, some studies are of a general cessation intervention including NRT, and not just of NRT, so risk differences may result from other aspects of the intervention. Also, some studies involve a group prescribed or recommended NRT, with many subjects possibly ignoring the advice and never using NRT. Another issue is that the analyses typically compare the groups initially allocated (as “intention-to-treat” analyses), not distinguishing risks in those who quit, cut down, or continue to smoke as before. Nor do they distinguish events in periods where the subjects were still using NRT and in those where they had quit. Also, there is very little information on risk by type of NRT used, or on dose–response, with results not related to the prescribed NRT dose. Finally, there is inconsistent reporting of adverse events, with differing classifications in different studies.

Turning now to epidemiological studies, a major issue is confounding by other variables, which affect risk of an SAHE and differ between the NRT and non-NRT groups. However, epidemiological studies can provide results for never smokers, or for those who both smoke and use NRT, and a few studies do adjust for changes in smoking post-NRT. If NRT users have no significant excess risk compared to non-smokers, then this suggests no effect of NRT, though issues of power and confounding remain. If, alternatively, similar increases are seen in NRT users and smokers, then this may mean risks are nicotine-related, with the dose of nicotine being unchanged. However, it may also mean the study was too small or too short term to detect differences in risk of an outcome resulting from chronic exposure. An increase in risk in the NRT group, but less than in smokers, could be because quitting reduces the dose of nicotine, or of other tobacco components. Clearly, the overall evidence is limited, with some findings difficult or impossible to interpret reliably in terms of an effect (or lack of effect) of NRT.

### Comparison with Reproductive/Developmental Reviews

It is interesting to compare our findings with those of some of the reviews we identified. The first, published in 2008, was mainly concerned with effects of smoking (Rore et al. 2008) and included little on possible health effects of NRT, though it mentioned one study (Wisborg et al. 2000) as providing evidence “that nicotine as present in NRT products does not have an adverse effect on infant weight”, and another (Kapur et al. 2001) as suggesting that rapid fetal movements seen in a single pregnant woman may have been due to nicotine withdrawal. Another review, concerning the role of pharmacotherapy for smoking cessation, was published in 2009 (Oncken and Kranzler 2009). Again, no clear conclusions were drawn, most attention being given to NRT use possibly leading to an increase in birthweight. They considered the higher congenital malformation rates compared to non-smokers in the Danish Birth Cohort Study (Morales-Suarez-Varela et al. 2006) “should be interpreted with caution” and also noted the lack of relationship with stillbirth (Strandberg-Larsen et al. 2008).

In 2010, two reviews were published. One, which concerned long-term consequences of fetal and neonatal nicotine exposure (Bruin et al. 2010), evaluated evidence from six studies we considered (see CAFs 2, 3, 8, 10, 13 and 14), but reached no overall conclusions, except that “the safety of NRT use during pregnancy has been evaluated in a limited number of short-term human trials, but there is currently no information on the long-term effects of developmental nicotine exposure in humans”. The other, “nicotine replacement therapy effect on pregnancy outcomes” (Forinash et al. 2010), considered five of the studies (CAFs 2, 10, 12, 13, 14), the authors concluding that “if behaviour modification therapy is attempted without success, NRT should be offered because of decreased risk of low birthweight and preterm delivery compared to continued smoking. Additionally, NRT does not appear to increase the risk of malformations”.

Two further reviews, in 2011 and 2012, were both by the same group. The first (Coleman et al. 2011) concerned efficacy and safety of NRT for smoking cessation during pregnancy, while the second (Coleman et al. 2012a) was a Cochrane review of pharmacological interventions for promoting smoking cessation during pregnancy. The first review (Coleman et al. 2011) considered five RCTs, one (Hotham et al. 2006) giving no relevant safety results, the other four considered in CAFs 10, 11, 13 and 14. The authors concluded “there is currently insufficient evidence to determine whether or not nicotine replacement therapy is effective or safe when used in pregnancy for smoking cessation; further research and, in particular, placebo-randomized controlled trials are required”. The Cochrane review was also restricted to RCTs, considering one additional trial (CAF 16). The conclusion was essentially the same.

### Comparison with Cardiovascular Disease Reviews

The only relevant review for CVD (Mills et al. 2014) concerned cardiovascular events associated with smoking cessation pharmacotherapies and was based on RCTs of NRT, bupropion and varenicline. The authors concluded “there was an elevated risk associated with nicotine replacement therapy that was driven predominantly by less serious events (RR 2.29; 95 % CI 1.39–3.82). When we examined major adverse cardiovascular events, we found … no clear evidence of harm with … nicotine replacement therapy (RR 1.95; 95 % CI 0.26–4.30)” (though the relevant table in the review gives CI of 0.92–4.30. These estimates derived from a “network meta-analysis”, incorporating also results from studies comparing results from other comparisons, such as bupropion (or varenicline) versus placebo, or versus NRT. A more conventional meta-analysis based simply on the 21 RCTs of NRT versus placebo gives a lower estimate, 1.38 (0.58–3.26), with a similar value, 1.48 (0.42–5.19), based on the three RCTs in high-risk patients.

### Relevance to nicotine-based tobacco products

We suggest that the conclusions from this systematic review on the potential SAHEs of pharmaceutical NRT use may help to predict health effects of nicotine exposure from nicotine-based tobacco products. Like NRT products, nicotine-based tobacco products contain nicotine as an active ingredient, do not contain tobacco and deliver nicotine via several different routes (oral and inhalation at present). Unlike NRT products, nicotine-based tobacco products are regulated as tobacco products and not licensed as drugs or medicines. Examples include products such as ENDS (e-cigarettes). Though ENDS products are relatively new, about 4 % of adults in the USA (US) and in the UK currently use e-cigarettes every day or some days (Pearson et al. 2012; Royal College of Physicians 2016; Schoenborn and Gindi 2015). Unfortunately, we know little about the potential SAHEs with use of these new products.

Even though these nicotine-based products are reportedly used for different purposes (smoking cessation or recreation), there appear to be many similarities in the nicotine delivery between NRT products and nicotine-based tobacco products. First, the source of added nicotine in all of these products is the same, pharmaceutical grade, derived from tobacco (Flora et al. 2015). Secondly, the route of exposure and the average maximum plasma nicotine concentration (C_max_) in the blood of NRT users (4 mg nicotine gum or inhaler) and ENDS users are similar, typically ranging from 5 to 40 ng nicotine/ml plasma (D’Ruiz et al. 2015; Haussmann and Fariss 2016; Schneider et al. 2001; St. Helen et al. 2015; Vansickel and Eissenberg 2013). Thus, as potential serious adverse health effects of nicotine per se are presumably dependent on the inherent toxicity of the active ingredient, nicotine per se and the level of exposure, these two parameters appear to be very similar in both NRT and nicotine-based products. Unfortunately for commercially available ENDS products, the enormous diversity in product design and aerosol delivery makes generalization about potential adverse health effects of these products challenging. Recent regulatory guidance in the US, UK and Europe, however, should standardize this category, resulting in well-characterized commercial ENDS products in the near future (Royal College of Physicians 2016; US Food and Drug Administration 2016).

Our review does not consider evidence on the potential adverse health effects related to long-term use of smokeless tobacco (SLT) including snus as a surrogate for NRT use. Firstly, these products differ in that a SLT user is not only exposed to nicotine extracted from this tobacco product, but also exposed to extracted compounds that may stimulate or mask a potential toxic effect of nicotine (Gong et al. 2014; Hecht et al. 1986; Hoffman et al. 1987; Prokopczyk et al. 1987). Secondly, extensive evidence on the potential adverse health effects of snus use has been thoroughly reviewed in various publications. For example, epidemiological evidence (Lee 2011, 2013) clearly demonstrates that snus use is not associated with an increased risk of cancer, heart disease or stroke, and provides scant support for any major adverse health effect. These findings are consistent with nicotine having very little effect on risk of SAHEs.

### Comparison with authoritative body conclusions

#### Overall conclusions

The only SAHE from NRT exposure we identified is an increased incidence of respiratory congenital abnormalities reported in one study. For many of the SAHEs considered, inadequate scientific evidence was available to determine reliably whether NRT has an effect. Thus, except for the observed developmental effect, we consider the scientific evidence, to date, does not support an association between NRT exposure and SAHEs (Table 20).

**Table 20:** Strength of evidence for serious^a^ adverse health effects of NRT

Potential health effects	Summary of evidence	Assessment^b^
Cancer	One epidemiology study found no association of NRT with lung, GI or overall cancer	Inadequate evidence
Respiratory congenital abnormalities	One epidemiology study found an increased incidence with NRT exposure	Limited evidence suggesting an effect
Fetal loss, spontaneous abortion, birthweight, gestational age, preterm birth, neonatal intensive care admissions and overall birth defects	Evidence from multiple epidemiology and clinical studies is consistent with a lack of effect of NRT	Limited evidence suggesting a lack of effect
Other reproductive/developmental: head circumference/length of infant, neonatal death and Apgar score	Evidence from a small number of studies is consistent with a lack of effect of NRT	Inadequate evidence
Circulatory disease: AMI incidence, CVD mortality, CVD admissions/re-admissions	Evidence from multiple epidemiology and clinical studies is consistent with a lack of effect of NRT	Limited evidence suggesting a lack of effect
Stroke	Evidence from a small number of studies is consistent with a lack of effect of NRT	Inadequate evidence
Other serious adverse health effects	Evidence from a small number of studies is consistent with a lack of effect of NRT	Inadequate evidence

^a^ Serious adverse health effects: adverse events that lead to hospitalization, significant disability or birth defects are life-threatening or result in death or require medical intervention to prevent one of the above outcomes (Little and Ebbert 2015; US Food and Drug Administration 2015b)

^b^Limited evidence suggesting an effect: A positive association was observed between exposure to NRT and this SAHE, but chance, bias and confounding factors could not all be ruled out with reasonable confidence. Conclusive studies are lacking. Inadequate evidence: The available studies are of insufficient quality, consistency or statistical power to permit a conclusion regarding a positive association between exposure to NRT and this SAHE. This category includes no data or conflicting evidence in multiple studies. Limited evidence suggesting a lack of effect: A statistically significant association was not observed between exposure to NRT and this SAHE, but a true relationship could not be ruled out with reasonable confidence because of a lack of statistical power, bias and/or confounding

The conclusions of our review agree with statements published by several authoritative bodies including Royal College of Physicians, NICE, FDA, and the US Surgeon General (National Institute for Health and Care Excellence 2013; Royal College of Physicians 2016; US Food and Drug Administration 2013; US Surgeon General 2014). For example, FDA (US Food and Drug Administration 2013) stated that they “examined the use of NRT products over periods larger than 12 weeks” and “and have not identified any safety risks associated with such use”. In addition, a NICE report (Jones et al. 2012) concluded that “no authors have attributed serious adverse events to NRT when used as part of smoking harm reduction”.

#### Cancer

We consider the strength of scientific evidence inadequate to associate NRT exposure with cancer (Table 20). Only one well-conducted study was identified, which found no evidence for an effect of NRT exposure on lung cancer, GI cancer or overall cancer (Murray et al. 2009). While a study clearly demonstrating a lack of adverse effect (cancer) associated with NRT exposure would normally lead to a conclusion of “limited evidence for a lack of serious adverse effect”, our assessment of inadequate evidence took into account the study’s limited follow-up time and the difficulty of reliably separating effects of NRT from those associated with a reduction in smoking.

Similar conclusions were reached from recent reports from the US Surgeon General (2014) which stated that “there are insufficient data to conclude that nicotine causes or contributes to cancer in humans” and from the National Institute for Health and Care Excellence (2013) which stated that “the results of this multicentre randomized controlled trial (Murray et al. 2009) suggest that long-term use of NRT is not associated with an increased incidence of harm, including cardiovascular events or cancer, with the latest analysis of outcome at 12.5 years from study outset”.

#### Reproductive/developmental effects

Based on one epidemiology study that demonstrated an increased incidence in respiratory congenital abnormalities, we consider there is limited evidence of a serious reproductive/developmental effect (lung development) with NRT exposure (Table 20). It is interesting to note that numerous animal studies have demonstrated an association between prenatal nicotine exposure and lung development abnormalities and offspring respiratory disease (Rehan et al. 2012; Spindel and McEvoy 2016).

In addition to congenital abnormalities (birth defects), nine additional endpoints were examined in numerous epidemiology and clinical studies. These showed no evidence of a significantly poorer outcome in NRT users. For some commonly studied conditions (fetal loss and spontaneous abortion, birthweight, gestational age, preterm birth, neonatal intensive care admissions and overall incidence of birth defects), the strength of evidence indicates limited evidence of a lack of effect from NRT exposure (Table 20). In fact, some evidence suggests infants born to pregnant smokers allocated to NRT use have increased birthweight and gestational age at birth, and better development of their offspring at 2 years. For the other endpoints considered (head circumference/length of infant, neonatal death and Apgar score), an assessment of inadequate evidence was reached.

FDA classifies NRT therapies as Category “C” or “D” developmental hazards depending on the type of NRT product. For example, nicotine gum is given a Pregnancy Category “C” classification, indicating that animal studies demonstrating an adverse effect may or may not have been conducted as well as the absence of adequate and well-controlled studies in pregnant women. In contrast, nicotine patches and inhalers are classified as a Pregnancy Category “D”, indicating that there are adequate well-controlled studies in pregnant women demonstrating a risk to the fetus (Dempsey and Benowitz 2001; Meadows 2001). These FDA warnings suggest a potential difference in risk between different types of NRT products. Our critical examination of the literature does not support such a difference. In fact, most published studies do not report the type of NRT product used. In the few studies clearly distinguishing types of NRT product, no difference was seen for reproductive/developmental end points such as birthweight and stillbirths, according to whether nicotine gum, patch or inhaler was used (Lassen et al. 2010; Strandberg-Larsen et al. 2008).

A recent report (US Surgeon General 2014) states “the evidence is sufficient to infer that nicotine adversely affects maternal and fetal health during pregnancy, contributing to multiple adverse outcomes such as preterm delivery and still birth”. However, this conclusion was inferred from studies of smokeless tobacco users, not from use of NRT products or nicotine per se delivery systems. Unlike NRT, the use of smokeless tobacco products results in exposure to tobacco extracts that contain, in addition to nicotine, numerous potential cytotoxic and cytoprotective compounds (Hoffman et al. 1987). Such a complex mixture complicates the interpretation of the study results with regard to the role nicotine per se in the observed adverse effects.

#### Cardiovascular disease

We consider there is limited evidence for a lack of serious cardiovascular effects associated with NRT use (Table 20). The cardiovascular effects examined in numerous epidemiological and clinical studies include the effect of NRT exposure on AMI, deaths caused by CVD and hospital admissions or readmissions for various cardiac-related issues. In general, no significant overall effect was observed, though many studies had limitations such as few relevant events, short follow-up and failure to control for changes in smoking behaviour subsequent to NRT use. The type of NRT product used did not appear to have a significant influence on serious adverse cardiovascular effects observed (Hubbard et al. 2005).

As evidence for a lack of increased incidence of or effect on cardiovascular disease, NICE (National Institute for Health and Care Excellence 2013) cite six studies evaluating the safety of NRT products in patients with cardiac disease and five randomized trials evaluating biomarkers of potential cardiac disease in the plasma of patients treated with NRT for smoking cessation.

#### Other serious adverse health effects

NRT product warning label statements (health-related) provide authoritative bodies the opportunity to communicate their concern about the potential harmful effects of these products. For NRT products, the labels warn about potential risks to the child (reproductive/developmental), the heart (cardiac disease), the vascular system (high blood pressure), the digestive system (stomach ulcers) and diabetes.

Based on these warnings and the findings from non-clinical studies, we expanded our literature search to include terms associated not only with CVD, reproductive and developmental effects and cancer but also with diabetes, stroke, GI tract, stomach, pancreas, pancreatic and kidney disease. These other SAHEs following NRT exposure were investigated in patients and healthy population studies. Our strength of evidence assessment concluded there was inadequate evidence to associate NRT exposure with stroke or these other SAHEs (Table 20).

## Conclusions

We critically evaluated 34 epidemiological studies and clinical trials relating NRT exposure to cancer, reproductive/developmental effects, CVD, stroke and/or other SAHEs in patients and in healthy populations. The overall evidence suffers from many limitations, the most significant being short-term exposure (≤12 weeks) and follow-up to NRT product use in most of the studies and the common failure to account for changes in smoking behaviour following NRT use. The only SAHE from NRT exposure we identified was an increased incidence of respiratory congenital abnormalities reported in one study. Other findings include limited evidence for a *lack* of any SAHE of NRT use on CVD and a range of reproduction/developmental endpoints. For cancer, stroke and other SAHEs, the evidence was judged inadequate. Though data are limited, there is no evidence that observed associations (or lack of associations) vary by type of NRT product (gum, patch, inhaler) used. Our overall conclusions from this systematic review agree with recent statements from authoritative bodies including FDA, the US Surgeon General, Royal College of Physicians and NICE.

## Electronic supplementary material

Below is the link to the electronic supplementary material.

Supplementary material 1 (DOCX 276 kb)

## Abbreviations

ADHD: Attention-deficit/hyperactivity disorder
AMI: Acute myocardial infarction
CAF: Critical assessment form
CI: Confidence interval
CVD: Cardiovascular disease
ENDS: Electronic nicotine delivery systems
FDA: Food and Drug Administration
GI: Gastrointestinal
HR: Hazard ratio
NICE: National Institute for Health and Care Excellence
NRT: Nicotine replacement therapy
OR: Odds ratio
RCT: Randomized clinical trial
RR: Relative risk
SAHE: Serious adverse health effect
