# Supplementary material for: A systematic review of possible serious adverse health effects of nicotine replacement therapy
Source: Arch Toxicol. 2016 Oct 3;91(4):1565–94. doi: 10.1007/s00204-016-1856-y (PMC5364244; doi:10.1007/s00204-016-1856-y)
Supplement: Supplementary file 1 — Supplementary material 1 (DOCX 276 kb) [file 204_2016_1856_MOESM1_ESM.docx]

**A systematic review of possible serious adverse health effects of nicotine replacement therapy**

Peter N Lee^1^ and Marc W Fariss^2^

^1^ P N Lee Statistics and Computing Ltd, Sutton, Surrey, United Kingdom

^2^ Altria Client Services LLC, Richmond, Virginia, United States of America

##### SUPPLEMENTARY FILES

| File | Content | Page |
| --- | --- | --- |
|  |  |  |
| 1 | Critical assessment forms (CAFs) | 2 |
|  |  |  |
| 2 | Study quality and risk of bias | 85 |
|  |  |  |
| 3 | Studies on NRT and reproduction/development | 93 |
|  |  |  |
| 4 | Studies on NRT and CVD | 95 |
|  |  |  |
| 5 | Studies in patients of NRT and other serious adverse health effects | 97 |
|  |  |  |
| 6 | Meta-analyses of NRT and other serious adverse health effects | 98 |
|  |  |  |
| References | | 99 |

##### SUPPLEMENTARY FILE 1

##### Critical assessment forms (CAFs)

**Notes for completing the CAF for individual epidemiological studies and clinical trials**

| Form No. | The number of the CAF. If one study is used for multiple topics keep the same number, but use multiple versions labelled e.g. 26A, 26B, 26C. |
| --- | --- |
| Topic | Give topic (e.g. CVD, followed by “Epidemiology” or “Clinical Trials” as appropriate. |
| Author(s) | The author(s) of the paper being assessed, followed by the Reference. |
| Title | The title of the paper. |
| Source | The journal name, year, volume and page numbers. |
| Study type | Epidemiological prospective cohort, case-control, case-series or cross-sectional study; randomized clinical trial (RCT) or non-randomized clinical trial. |
| Study location | City, (State), Country. |
| Populations studied and inclusion criteria | Description of those included in and excluded from the study. |
| Nicotine exposures | e.g. Nicotine gum, nicotine patch, NRT not further defined, including dose and dosing regimen if available. |
| Treatment groups and sizes | Describe the groups compared and the numbers in the groups. |
| Relevant endpoints | Restrict attention mainly to the serious adverse health effects to be considered in the report. |
| Confounding variables | Which variables were controlled for in the design (matching factors, randomization) or analysis. |
| Other relevant study details | Aspects of the study not described above, e.g. power of study, statistical methods. |
| Relevant findings | Summarize results for adverse health effects, if necessary estimating appropriate statistics (e.g. relative risk (RR), mean difference) from data provided in the source. |
| Authors’ main relevant conclusions | Mention conclusions relating to adverse health effects of interest. Give relevant quotes, as appropriate. |
| Strengths and weaknesses mentioned | Only refer to those mentioned to by the author. |
| Study quality score/Risk of bias | For epidemiology studies, assess study quality as good, fair or poor based on the NIH published quality assessment tools for observational cohort and cross-sectional studies (National Heart Lung and Blood Institute 2014b) and for case-control studies (National Heart Lung and Blood Institute 2014a). For clinical trials, assess risk of bias using the Cochrane Collaboration’s tool, (Higgins et al. 2011). Each assessment involves scoring on various criteria, the results of which are given in Supplementary File 2. |
| Comments | Emphasise major strengths and weaknesses, and the factors that have contributed particularly to the study quality score. Refer to difficulties in the interpretation of the results specific to the study. |

**Notes for completing the CAF for published meta-analyses**

| Form No. | The number of the CAF. |
| --- | --- |
| Topic | Will always be “Other serious adverse health effects – meta-analysis of smoking cessation trials in healthy individuals.” |
| Author(s) | The author(s) of the paper being assessed, followed by the Reference. |
| Title | The title of the paper. |
| Source | The journal name, year, volume and pages. |
| Meta-analysis type | State whether it is just a meta-analysis or a systematic review and meta-analysis, and give the number of studies combined. |
| Location of studies | State whether the meta-analysis was restricted on location, and summarize details given of the distribution of studies over country. |
| Populations studied and inclusion criteria | Description of studies to be included and excluded. |
| Searches | Give sources used and search forms if available. |
| Nicotine exposures | Summarize information on types of NRT included. |
| Numbers of subjects considered | Summarize overall numbers of subjects considered. |
| Relevant endpoints | Restrict attention mainly to the serious adverse health effects to be considered in the report. |
| Relevant findings | Summarize results for serious adverse health effects. |
| Authors’ main relevant conclusions | Mention results relating to adverse health effects of interest. Give relevant quotes, as appropriate. |
| Study quality assessment | Describe the procedure used by the authors to assess study quality and risk of bias. |
| Strengths and weaknesses mentioned | Only refer to those mentioned by the author. |
| Comments | Emphasise major strengths and weaknesses. |

**Index by endpoint and study type**

| Endpoint | Epidemiology | Clinical trials |
| --- | --- | --- |
|  |  |  |
| Cancer | 1 | - |
| Reproduction/development | 2-9 | 10-20 |
| CVD | 21, 22A, 23-26 | 27, 28A, 29, 30A |
| Stroke | 22B, 31 | 28B |
| Other serious health effects in patients | 32 | 28C, 30B, 33, 34 |
| Other serious health effects in healthy individuals | - | 35-38 |

Note that the forms are presented by endpoint, then by study type and then in chronological order of publication. There are 38 studies, with 22, 28 and 30 providing results for more than one endpoint.

**Completed critical assessment forms**

| **Form No.** | **1** |
| --- | --- |
| Topic | Cancer – Epidemiology |
| Author(s) | R P Murray et al. (2009) |
| Title | “Does nicotine replacement therapy cause cancer? Evidence from the Lung Health Study” |
| Source | Nicotine & Tobacco Research, 2009, 11, 1076-1082 |
| Study type | Prospective study |
| Study location | 10 clinical centres in the United States and Canada |
| Population studied and inclusion criteria | Participants in the Lung Health Study, an RCT of smoking cessation in middle-aged volunteers of both sexes with asymptomatic airways obstruction who were randomized to a smoking intervention where they were encouraged to use NRT (Nicorette 2mg gum) liberally for 6 months and then to stop NRT use after 2½ years. All participants in the analysis were alive and cancer-free at the end of the 5-year period of the study, the point from which cancer follow-up started. |
| Nicotine exposures | Mean NRT use in pieces per day over the 5 years of the Lung Health Study. |
| Treatment groups and sizes | 3,315 subjects, of which 1,986 had used NRT. |
| Relevant endpoints | Fatal and non-fatal cases of lung cancer (75), gastrointestinal cancer (33) and all cancer (203) occurring during the 7.5 year follow-up period ending in December 2001. |
| Confounding variables | Baseline age, sex, cigarettes per day, and lifetime pack-years of smoking. |
| Other relevant study details | Analyses use Cox proportional hazards methods to model time-to-event data. The analyses related each endpoint to NRT use (pieces of gum per day) and pack-years cigarette use over the 5-year period of the Lung Health Study, individually and in combination, with adjustment for the confounding variables. |
| Relevant findings | HRs (95% CIs) for mean daily NRT use were as follows:  Adjustment for pack-years  cigarette use over 5 years  No Yes  Lung cancer 1.02 (0.95-1.09) 1.04 (0.97-1.12)  GI cancer 0.97 (0.86-1.10) 0.97 (0.82-1.14)  All cancer 1.00 (0.96-1.05) 1.01 (0.97-1.06)  There was also no relationship of any type of cancer to NRT use, when average NRT use was replaced by any NRT use in any analysis.  There was a significant relationship of pack-years cigarette use over 5 years to lung cancer (1.08, 1.01-1.16 unadjusted, and 1.10, 1.02-1.19 adjusted for daily NRT use). |
| Authors’ main relevant conclusions | “The absence in general of a relation between nicotine replacement therapy and cancer across the models adds credence to our conclusion that nicotine replacement therapy does not cause cancer.” |
| Strengths and weaknesses mentioned | “The sample size, close monitoring of the use of nicotine replacement therapy and cigarettes, and the well-documented outcomes of this study will be difficult for future studies to match.”  “There is evident confounding between historical smoking and current smoking and between current smoking and current nicotine replacement therapy use.”  “Whatever the exposure time necessary for smoking to result in cancer, it is usually not assumed to be as brief as 5 years.”  “Nicotine gum provides low mean doses of nicotine relative to smoking.” |
| Study quality score | GOOD |
| Comments | This is a well conducted and well analysed study. It is probably the best that can currently be done for an endpoint with a long latent period such as cancer, though residual confounding by smoking remains possible. |

| **Form No.** | **2** |
| --- | --- |
| Topic | Reproduction/Development - Epidemiology |
| Author(s) | M M Morales-Suarez-Varela et al. (2006) |
| Title | “Smoking habits, nicotine use, and congenital malformations” |
| Source | Obstetrics & Gynecology, 2006, 107, 51-57 |
| Study type | Prospective study |
| Study location | Denmark (National Birth Cohort) |
| Population studied and inclusion criteria | Women who gave birth in 1997-2003 who answered questions, during weeks 11-25 of gestation, on smoking habits and NRT use in pregnancy. Where a woman had multiple pregnancies in the period, only the first is considered. Pregnancies in women with ovarian or cervical cancer or who gave birth to twins or triplets were also excluded. |
| Nicotine exposures | Used NRT (patch, gum, inhaler) in pregnancy. |
| Treatment groups and sizes | 76,768 pregnant women of which 12,812 smoked and did not use NRT and 250 used NRT and did not smoke. |
| Relevant endpoints | Congenital malformations; all, major, and major musculoskeletal as classified by EUROCAT criteria. |
| Confounding variables | Maternal age, parity, pre-pregnancy BMI, alcohol intake and education were all considered as potential confounders for analyses of smoking and congenital malformations, but only age and alcohol intake were adjusted for. No adjustment factors were noted for analyses of NRT and congenital malformations. |
| Other relevant study details | It is clear from other publications in this cohort (e.g. see CAFs 3 and 5), that only about 30% of all Danish pregnant women participated. |
| Relevant findings   \|  \|  \| Major congenital malformations \| \| \|  \| Relative prevalence rate (95% CI) \| \| \| \| --- \| --- \| --- \| --- \| --- \| --- \| --- \| --- \| --- \| \| Habits wk 12 of pregnancy \| N \| All \| Major \| MMS^1^ \|  \| All \| Major \| MMS^a^ \| \| No smoking or NRT \| 55,915 \| 2,719 \| 2,168 \| 673 \|  \| 1.00 \| 1.00 \| 1.00 \| \| Smoked ≤10/day (no NRT) \| 12,365 \| 651 \| 535 \| 157 \|  \| 1.09(1.00-1.19) \| 1.12(1.02-1.23) \| 1.06(0.89-1.26) \| \| Smoked >10/day (no NRT) \| 4,447 \| 220 \| 187 \| 54 \|  \| 1.02(0.89-1.17) \| 1.09(0.93-1.27) \| 1.01(0.76-1.33) \| \| NRT (no smoking) \| 250 \| 19 \| 11 \| 6 \|  \| 1.61(1.01-2.58) \| 1.13(0.62-2.07) \| 2.05(0.91-4.63) \| \| NRT (no smoking) v Smoked (no NRT)^b^ \|  \|  \|  \|  \|  \| 1.50(0.94-2.41) \| 1.02(0.55-1.86) \| 1.96(0.86-4.45) \|   ^a^ MMS = major musculoskeletal abnormalities ^b^ Estimated | |
| Authors’ main relevant conclusions | “We identified an increase of malformations risk in nonsmokers using nicotine substitutes. This finding needs to be replicated in other data sources.” |
| Strengths and weaknesses mentioned | Associations “may be caused by uncontrolled confounding”. |
| Study quality score | GOOD |
| Comments | No mention of women who smoked and used NRT. Results “similar” if analyses restricted to first-born children. Small number of cases in congenital malformations in NRT users, even more so for specific types. |
| **Form No.** | **3** |
| Topic | Reproduction/Development - Epidemiology |
| Author(s) | K Strandberg-Larsen et al. (2008) |
| Title | “Use of nicotine replacement therapy during pregnancy and stillbirth: a cohort study” |
| Source | BJOG, 2008, 115, 1405-1410 |
| Study type | Prospective study |
| Study location | Denmark (National Birth Cohort) |
| Population studied and inclusion criteria | Women who gave birth in 1996-2002 who answered questions, during weeks 12-16 of gestation, on smoking habits and NRT use in pregnancy. Exclusions included women giving birth to twins or triplets, ectopic pregnancies, pregnancies ending in hydatidiform mole, and women not providing information relevant to the analysis. |
| Nicotine exposures | Used NRT (patches, gum, inhalers). |
| Treatment groups and sizes | 87,032 pregnant women of which 13,266 smoked only. 1,927 used NRT only and 1,091 used both. |
| Relevant endpoints | Stillbirth, defined as any fetus that did not breathe or show any other sign of life at birth after a minimum of 20 weeks of gestation. |
| Confounding variables | Analyses adjusted for maternal age and socio-occupational status; NRT/no NRT analyses also adjusted for smoking. Other variables were considered (parity, pre-pregnancy BMI, alcohol, coffee, planning of pregnancy) but found to have little material effect, as did type of NRT. |
| Other relevant study details | It is estimated that approximately half of all GPs in Denmark took part in this recruitment, and around 60% of the invited women chose to participate. |
| Relevant findings | \|  \| Stillbirths \| Adjusted HR (95% CI) \| \| --- \| --- \| --- \| \| No NRT \| 487 \| 1.00 \| \| NRT \| 8 \| 0.57 (0.28-1.16) \| \| No NRT or smoking \| 380 \| 1.00 \| \| Smoking only \| 107 \| 1.46 (1.17-1.82) \| \| NRT only \| 3 \| 0.67 (0.21-2.08) \| \| Both \| 5 \| 0.83 (0.34-2.00) \| \|  \|  \|  \| |
| Authors’ main relevant conclusions | “Our study suggests that NRT use during pregnancy has no serious impact on the risk of stillbirth.” |
| Strengths and weaknesses mentioned | Strengths : prospective data, large cohort, almost complete follow-up, comprehensive confounder adjustment, few missing data  Limitations : few NRT users, limited number of expected stillbirths, data on NRT not detailed, is self-reported, and only collected in early pregnancy, 60% participation rate in study. |
| Study quality score | GOOD |
| Comments | Excluding subsequent pregnancies for the same woman, and changing cut-off for stillbirth definition from 20 to 22 weeks did not affect the results. |

| **Form No.** | **4** |
| --- | --- |
| Topic | Reproduction/Development - Epidemiology |
| Author(s) | T H Lassen et al. (2010) |
| Title | “Maternal use of nicotine replacement therapy during pregnancy and offspring birthweight: a study within the Danish National Birth Cohort” |
| Source | Paediatric and Perinatal Epidemiology, 2010, 24, 272-281 |
| Study type | Prospective study |
| Study location | Denmark (National Birth Cohort) |
| Population studied and inclusion criteria | Women who gave birth in 1996-2002, and provided information on NRT and potential confounders in the second and third trimesters of pregnancy. Inclusion criteria included: singleton livebirths, born after 27th week of pregnancy; completed second interview after the 27th week of pregnancy; certain determination of gestational age; ≤2 weeks discrepancy between expected day of delivery stated in the first and second interview. |
| Nicotine exposures | Used NRT in the first 27 weeks of gestation; any, patch only, gum only, inhaler only or more than one. Also used NRT in the first 35 weeks, in a subcohort. |
| Treatment groups and sizes | Pregnant women of which 1828 used NRT in the first 27 weeks of gestation, 56.3% gum, 30.4% patch, 27.3% inhaler and 10.0% more than one. After excluding those with missing data, 68,156 women were included in the 27 week analysis and 15,238 in the 36 week analysis. |
| Relevant endpoints | Adjusted mean change in mean birthweight (g) per one week use of NRT in first 27 (35) weeks of gestation. |
| Confounding variables | Gestation age, smoking up to 27 (or 35) weeks of gestation, smoking status of partner, parity, pre-pregnancy body mass index, maternal height, alcohol consumption, coffee intake, physical exercise, infant sex, socio-economic status, weight loss, eating disorder, fertility problems, vaginal bleeding, nausea, hypertension. Analysis of NRT use in the third trimester also adjusted for use in the first two trimesters. |
| Other relevant study details | The analyses also took into account the fact that 4112 women had more than one pregnancy in the study period. It is clear from other publications in this cohort (e.g. see CAFs 2 and 5), that only about 30% of all Danish pregnant women participated. |
| Relevant findings | \|  \|  \| Changes in mean birthweight (g) \| \| \| --- \| --- \| --- \| --- \| \| NRT use \| Period of gestation \| b \| 95% CI \| \| Total \| First 27 weeks \| 0.25 \| (-2.31, 2.81) \| \| Patch only \| First 27 weeks \| -4.37 \| (-13.34, 4.60) \| \| Gum only \| First 27 weeks \| 0.48 \| (-2.51, 3.48) \| \| Inhaler only \| First 27 weeks \| 6.19 \| (-0.40, 12,79) \| \| More than one \| First 27 weeks \| -10.73 \| (-26.51, 5.05) \| \| Total \| First 35 weeks \| 0.87 \| (-3.41, 5.15) \| \| Total \| In third trimester \| 0.30 \| (-24.97, 25.56) \| \|  \|  \|  \|  \| |
| Authors’ main relevant conclusions | “The results of this study suggest that maternal use of NRT in pregnancy does not seriously affect birthweight, but there could be a negative effect on birthweight associated with simultaneous use of more than one type of NRT product.” |
| Strengths and weaknesses mentioned | “Although the number of NRT users in this study is not large, it is the largest cohort study that has investigated the association between NRT and birthweight. Strengths of this study are the prospectively collected information on the use of NRT and the detailed control for confounders based on information on a great number of other relevant exposures during pregnancy.” Limitations referred to include the smaller sample size for NRT use in trimester 3, and reliance on self-report of NRT use.” |
| Study quality score | GOOD |
| Comments | A clear effect of smoking on birthweight was seen, with a highly significant (p < 0.001), more than 200 g reduction in the heavier smokers. The association with use of multiple NRT products was not significant. The results for NRT use did not vary by the timing of the birth. |

| **Form No.** | **5** |
| --- | --- |
| Topic | Reproduction/Development - Epidemiology |
| Author(s) | T Torp-Pedersen et al. (2010) |
| Title | “In-utero exposure to smoking, alcohol, coffee, and tea and risk of strabismus” |
| Source | American Journal of Epidemiology, 2010, 171, 868-875 |
| Study type | Prospective study |
| Study location | Denmark (National Birth Cohort) |
| Population studied and inclusion criteria | Children born alive in 1996-2003 whose mothers were interviewed at gestational weeks 12-14 and 30-32, and 6 months post-delivery. No mention of any other inclusion or exclusion criteria. |
| Nicotine exposures | Use of NRT during pregnancy |
| Treatment groups and sizes | 96,842 children. Number whose mothers used NRT not stated but estimated to be about 2800. |
| Relevant endpoints | Strabismus (a common eye disorder) overall, and by types: congenital esotropia (CES), partially and fully accommodative esotropia (PFES), all exotropia (AEX), and other. Strabismus was identified from medical records. |
| Confounding variables | Year of birth, social class, maternal age at birth, maternal smoking dose, and maternal coffee and tea consumption |
| Other relevant study details | Of the women who were invited to participate in the Danish National Birth Cohort, only 60% accepted. (Also note that only about 50% of GPs participated – see CAF 3.) |
| Relevant findings | \| Strabismus type \| Exposed cases \| Adjusted RR (95% CI) \| \| --- \| --- \| --- \| \| Any \| 61 \| 1.22 (0.92-1.61) \| \| CES \| 13 \| 1.53 (0.81-2.91) \| \| PFES \| 18 \| 1.07 (0.64-1.80) \| \| AEX \| 11 \| 1.58 (0.80-3.10) \| \| Other \| 19 \| 1.09 (0.68-1.76) \| \|  \|  \|  \| |
| Authors’ main relevant conclusions | “Nicotine replacement therapy was not associated with strabismus risk (RR = 1.22, 95% CI: 0.92, 1.61).” |
| Strengths and weaknesses mentioned | Strengths : Large, population-based cohort; case-finding maximized; accurate typing of strabismus  Weaknesses : Incomplete participation; some missing medical records; self-reported exposure. |
| Study quality score | GOOD |
| Comments | Results not affected when analyses restricted to eldest child, where women had more than 1 child in the cohort. The adjusted RR for maternal smoking for any strabismus was similar, 1.26, but significant (95% CI 1.11-1.43), so failure to see a significant effect for NRT may have been due to lack of power. |

| **Form No.** | **6** |
| --- | --- |
| Topic | Reproduction/Development - Epidemiology |
| Author(s) | I Milidou et al. (2012) |
| Title | “Nicotine replacement therapy during pregnancy and infantile colic in the offspring” |
| Source | Pediatrics, 2012, 129, e652-e658 |
| Study type | Prospective |
| Study location | Denmark (National Birth Cohort) |
| Population studied and inclusion criteria | Live-born singletons in 1996-2002 with complete information on nicotine exposure during pregnancy and infantile colic symptoms as recorded at 6 months of age. The main analyses were based on the first child where a mother had multiple births in the cohort. |
| Nicotine exposures | NRT use (patch, gum, inhaler) |
| Treatment groups and sizes | 63,128 children, with 207 mothers using NRT only, 15,016 smoking only, and 1,245 using NRT and smoking. |
| Relevant endpoints | Infantile colic in the first six months. |
| Confounding variables | Maternal age, parity, coffee consumption, alcohol consumption, binge drinking, and education/occupational status of couple. |
| Other relevant study details | Approximately 50% of the pregnant population was invited by their general practitioner to join the cohort at the first antenatal visit, and nearly 60% of those consented. |
| Relevant findings | \|  \| Exposed \|  \|  \| \| --- \| --- \| --- \| --- \| \| Exposure \| cases \|  \| Adjusted OR (95% CI) \| \| Unexposed \| 3397 \|  \| 1.0 \| \| NRT users \| 23 \|  \| 1.6 (1.0-2.5) \| \| Smokers \| 1417^a^ \|  \| 1.3 (1.2-1.4) \| \| Smoking and NRT \| 137 \|  \| 1.5 (1.3-1.8) \| \| NRT (in smokers) \|  \|  \| 1.15 (0.97-1.37)^b^ \| \| ^a^ Given incorrectly as 11417 in the source  ^b^ Estimated \| \| \| \| |
| Authors’ main relevant conclusions | “We corroborated the association between smoking and infantile colic after adjustment for several possible confounders in a large cohort study. Moreover, we found that infants exposed to NRT prenatally had an increased risk for infantile colic of the same magnitude as those exposed to tobacco smoke. Thus, nicotine may play a role in the pathogenesis of infantile colic.” |
| Strengths and weaknesses mentioned | Strengths : Large; population-based; prospective.  Weaknesses : Possible confounding; cannot distinguish effects of exposure in different trimesters or post-natally. |
| Study quality score | GOOD |
| Comments | Similar results if restricted to children born between 37 and 41 completed gestational weeks with a birthweight above 2500 g. Adjustment had no effect on ORs. |

| **Form No.** | **7** |
| --- | --- |
| Topic | Reproduction/Development - Epidemiology |
| Author(s) | J L Zhu et al. (2014) |
| Title | “Parental smoking during pregnancy and ADHD in children: the Danish National Birth Cohort” |
| Source | Pediatrics, 2014, 134, e382-e388 |
| Study type | Prospective |
| Study location | Denmark (National Birth Cohort) |
| Population studied and inclusion criteria | Singleton births in 1996-2003, with information on parental smoking reported in pregnancy. Exclusions included: unsuccessful pregnancies, pregnancies where mother emigrated or died, pregnancies with unknown birth outcomes, pregnancies resulting in twins or triplets, and births with missing birth dates or with missing information on maternal or paternal smoking. |
| Nicotine exposures | Mother used NRT (patch, gum, inhaler) in pregnancy. |
| Treatment groups and sizes | 84,803 singletons, of which, at around 16 weeks gestation, 13,547 of their mothers were current smokers (regardless of NRT), 814 used NRT (no current smoking), 7,366 had quit smoking, leaving 63,076 non-smokers. 50,870 of the children participated in the 7 year follow-up. |
| Relevant endpoints | Diagnosed or medicated attention-deficit/hyperactivity disorder (ADHD) from age 5 years to end of 2011, parent-rated hyperactivity/inattention score (HIS) from 7 year follow-up questionnaire, birthweight (g). |
| Confounding variables | Maternal age, parity, alcohol intake during pregnancy, parental socioeconomic status, parental psychopathology, and child’s gender. |
| Other relevant study details | It is clear from other publications in this cohort (e.g. see CAFs 3 and 5) that only about 30% of all Danish pregnant women participated. |
| Relevant findings   \|  \|  \|  \| Adjusted HR \| Regression \| Regression \| \| --- \| --- \| --- \| --- \| --- \| --- \| \| Exposure \|  \| Cases \| (95% CI) \| Coefficient (95% CI) \| Coefficient (95% CI) \| \| Mother \| Father \| ADHD \| ADHD \| HIS \| Birthweight \| \| Smoker \| Smoker \| 368 \| 1.83 (1.60-2.10) \| 0.54 (0.46 to 0.61) \| -248.3 (-262.0 to -234.5) \| \| Smoker \| Nonsmoker \| 164 \| 1.63 (1.36-1.94) \| 0.44 (0.35 to 0.53) \| -209.6 (-227.1 to -192.0) \| \| NRT \| Smoker \| 7 \| 1.28 (0.57-2.89) \| 0.39 (0.06 to 0.73) \| -18.7 (-95.1 to 57.7) \| \| NRT \| Nonsmoker \| 22 \| 2.28 (1.48-3.51) \| 0.45 (0.20 to 0.70) \| -15.8 (-63.8 to 32.2) \| \| Quit smoking \| Smoker \| 113 \| 1.70 (1.38-2.10) \| 0.30 (0.20 to 0.40) \| -0.4 (-21.0 to 20.3) \| \| Quit smoking \| Nonsmoker \| 83 \| 1.08 (0.85-1.36) \| 0.16 (0.07 to 0.25) \| 28.8 (11.3 to 46.2) \| \| Nonsmoker \| Smoker \| 360 \| 1.29 (1.14-1.47) \| 0.16 (0.11 to 0.21) \| -11.1 (-21.8 to -0.3) \| \| Nonsmoker \| Nonsmoker \| 892 \| 1.00 \| 0.00 \| 0.00 \| \| NRT v smoker \| (Estimated) \|  \| 1.11 (0.75-1.63) \| -0.08(-0.28 to 0.12) \|  \| \| Note: Numbers of cases HIS not given \| \| \| \| \| \| | |
| Authors’ main relevant conclusions | “We also saw a higher risk of ADHD in children of mothers who used nicotine replacement during pregnancy.”  “In conclusion, our findings suggest that exposure to prenatal tobacco smoke, possibly nicotine, may have a prenatal programming effect on the risk of ADHD in children. Alternatively, our findings may reflect confounding by family factors more linked to maternal than paternal smoking, which could be both genetic and postpartum caring factors.” |
| Strengths and weaknesses mentioned | Strengths: large number of children followed; several sources to define outcome; able to adjust for several confounders.  Weaknesses : Self-reported exposure data; few NRT users; moderate response at 7 years. |
| Study quality score | GOOD |
| Comments | None |

| **Form No.** | **8** |
| --- | --- |
| Topic | Reproduction/Development – Epidemiology |
| Author(s) | K H Gaither et al. (2009) |
| Title | “Does the use of nicotine replacement therapy during pregnancy affect pregnancy outcomes?” |
| Source | Journal of Maternal and Child Health, 2009, 13, 497-504 |
| Study type | Cross-sectional |
| Study location | USA; Colorado, Louisiana, Maine and Washington |
| Population studied and inclusion criteria | Women aged 18-45 years who in 2004 completed a questionnaire postnatally. Women were excluded who did not receive prenatal care or with missing data on marital status, education, parity, Medicaid or alcohol use. |
| Nicotine exposures | Prescription of nicotine spray, inhaler or pill, or recommended using a nicotine patch or gum during prenatal visits. |
| Treatment groups and sizes | 5,716 women, of which 225 were smokers prescribed or recommended NRT, 637 were other smokers, and 4,854 were nonsmokers |
| Relevant endpoints | Low birthweight (≤2500 g) or preterm birth (before 37 weeks gestation), assessed from birth certificates. |
| Confounding variables | Adjustment was made for age, marital status, education and race/ethnicity. Pre-pregnancy BMI, Medicaid, parity, alcohol use, and maternal weight gain during pregnancy found not to cause material confounding. |
| Other relevant study details | Data from the 2004 Phase V Pregnancy Risk Assessment Monitoring System (PRAMS). |
| Relevant findings   \|  \| Low birthweight \| \| \| Preterm birth \| \| \| \| --- \| --- \| --- \| --- \| --- \| --- \| --- \| \|  \| Cases \| Unadjusted OR (95%CI) \| Adjusted OR (95% CI) \| Cases \| Unadjusted OR (95%CI) \| Adjusted OR  (95% CI) \| \| NRT Recommended/Prescribed \| 84 \| 2.00 (1.13 - 3.45) \| 1.95 (1.10 - 3.46) \| 66 \| 2.05 (1.16 - 3.60) \| 2.05 (1.14 - 3.63) \| \| Smoker \| 205 \| 1.36 (0.98 - 1.88) \| 1.31 (0.92 - 1.87) \| 156 \| 1.09 (0.76 - 1.57) \| 1.09 (0.74 - 1.61) \| \| Nonsmoker \| 1303 \| 1.00 \| 1.00 \| 1165 \| 1.00 \| 1.00 \| \| NRT v smokers (estimated) \|  \| 1.47 (0.79 - 2.72) \| 1.49 (0.79 – 2.82) \|  \| 1.88 (0.99 – 3.58) \| 1.88 (0.97 – 3.56) \| | |
|  |  |
| Authors’ main relevant conclusions | “Risks of low birthweight and preterm birth were highest for women prescribed or recommended NRT. These findings may be related to frequency of maternal smoking. While heavier smokers may be more likely to be recommended NRT, they also may have the most difficulty with cessation. Greater efforts should be made to ensure that these women do successfully cease smoking.” |
| Strengths and weaknesses mentioned | Weaknesses included: lack of accurate information on amount smoked during the entire pregnancy; small sample size; smokers recommended/prescribed NRT may not have used NRT, and may be heavier smokers; reliance on birth certificate data, and possible residual confounding. |
| Study quality score | FAIR |
| Comments | ORs for NRT and for smoking do not actually differ significantly. |

| **Form No.** | **9** |
| --- | --- |
| Topic | Reproduction/Epidemiology – Epidemiology |
| Author(s) | N N Dhalwani et al. (2015) |
| Title | “Nicotine replacement therapy in pregnancy and major congenital anomalies in offspring” |
| Source | Pediatrics, 2015, 135, 859-867 |
| Study type | Prospective study |
| Study location | United Kingdom |
| Population studied and inclusion criteria | Children born in the United Kingdom between 2001 and 2012 to mothers aged 15-49 years with linked mother-child primary care records. Children with anomalies specifically attributed to known teratogens excluded. |
| Nicotine exposures | Prescription of NRT during first trimester of pregnancy or within 4 weeks before estimated conception date. |
| Treatment groups and sizes | 2677 women prescribed NRT, 9980 smokers in first trimester not prescribed NRT, 179,841 nonsmokers in first trimester. |
| Relevant endpoints | Major congenital abnormalities (MCAs) overall and by system-specific subgroups. |
| Confounding variables | Maternal age at conception, Townsend deprivation index score, maternal diabetes, asthma, mental illness, and multiple births. |
| Other relevant study details | Potential correlation between multiple births for same woman accounted for. |
| Relevant findings   \|  \| Prescribed NRT (N = 2677) \| \| Smokers (N = 9980) \| \| Nonsmokers  (N = 179841) \| \| --- \| --- \| --- \| --- \| --- \| --- \| \|  \| n \| HR (95% CI)^a^ \| n \| HR (95% CI)^a^ \| n \| \|  \|  \|  \|  \|  \|  \| \| All \| 90 \| 1.12 (0.90-1.39) \| 314 \| 1.05 (0.93-1.19) \| 5131 \| \| Heart \| 26 \| 1.01 (0.68-1.50) \| 104 \| 1.09 (0.88-1.35) \| 1652 \| \| Limb \| 15 \| 0.99 (0.59-1.66) \| 60 \| 1.04 (0.79-1.36) \| 996 \| \| Genital system \| 16 \| 1.14 (0.69-1.88) \| 51 \| 0.94 (0.70-1.27) \| 847 \| \| Urinary system \| 12 \| 1.82 (1.02-3.25) \| 20 \| 0.86 (0.54-1.36) \| 479 \| \| Chromosomal \| 4 \| 0.74 (0.27-2.05) \| 13 \| 0.71 (0.40-1.26) \| 383 \| \| Musculoskeletal \| 10 \| 1.79 (0.93-3.43) \| 28 \| 1.32 (0.87-2.01) \| 342 \| \| Orofacial cleft \| 3 \| 0.75 (0.24-2.34) \| 20 \| 1.40 (0.88-2.23) \| 252 \| \| Digestive system \| 6 \| 1.52 (0.66-3.48) \| 18 \| 1.30 (0.78-2.17) \| 261 \| \| Nervous system \| 4 \| 0.83 (0.30-2.27) \| 18 \| 1.01 (0.60-1.71) \| 273 \| \| Other malformations^b^ \| 5 \| 1.16 (0.47-2.84) \| 18 \| 1.08 (0.65-1.80) \| 257 \| \| Eye \| 3 \| 0.89 (0.28-2.85) \| 15 \| 1.19 (0.69-2.06) \| 208 \| \| Respiratory system \| 10 \| 4.65 (2.22-9.73) \| 10 \| 1.34 (0.67-2.67) \| 137 \| \| Genetic \| 0 \| - \| 9 \| 1.02 (0.47-2.21) \| 153 \| \| Abdominal wall \| 0 \| - \| 3 \| 0.77 (0.22-2.68) \| 29 \| \| Ear, face and neck \| 0 \| - \| 3 \| 1.51 (0.49-4.68) \| 35 \| \| ^a^ Adjusted for potential confounding factors noted above; 95% CI estimated from 99% CI reported \| \| \| \| \| \| \| ^b^ Including asplenia and conjoined twins \| \| \| \| \| \|   Comparing smokers prescribed and not prescribed NRT, HRs were 1.07 (0.84-1.36) for all MCAs and 3.49 (1.40-8.71) for respiratory system abnormalities. No significant (at p < 0.05) HRs were seen for any of the other MCAs listed above. | |
| Authors’ main relevant conclusions | “For most system-specific MCAs, we found no statistically significant increased risks associated with maternal NRT prescribed during pregnancy, except for respiratory anomalies” |
| Strengths and weaknesses mentioned | “Although this study is the largest published to date, NRT use in pregnancy remains rare; thus, the statistical power was limited. Higher morbidities in those women prescribed NRT may also be an explanatory factor. Nevertheless, absolute MCA risks were similar between women who smoked and those prescribed NRT during pregnancy.” |
| Study quality score | FAIR |
| Comments | Study of NRT prescription, not of NRT use. |

| **Form No.** | **10** |
| --- | --- |
| Topic | Reproduction/Development – Clinical trial |
| Author(s) | K Wisborg et al. (2000) |
| Title | “Nicotine patches for pregnant smokers: a randomized controlled study” |
| Source | Obstetrics and Gynecology, 2000, 96, 967-971 |
| Study type | Placebo-controlled double-blind RCT of smoking cessation. |
| Study location | Denmark, Aarhus |
| Population studied and inclusion criteria | Healthy pregnancy women who smoked 10+ cigs/day and <22 weeks pregnant and agreed to participate in a study involving smoking cessation counselling and patch use. 41% participation rate. |
| Nicotine exposures | Nicotine or placebo patches. Nicotine patches (16 hours/day) were 15 mg for 8 weeks and 10 mg for 3 weeks. |
| Treatment groups and sizes | 250 participants, 124 allocated to nicotine patch, 126 to placebo patch. |
| Relevant endpoints | Birthweight (g), low birthweight (<2500 g), and preterm delivery. No serious adverse events were reported. |
| Confounding variables | No adjustment for confounding variables in results presented. The two groups were found not to differ significantly on cigs/day, cotinine at first visit, previous quit attempts, Fagerström score, and maternal age, parity, marital status, years of schooling, occupational status, CAFfeine intake, and alcohol intake. |
| Other relevant study details | Power calculations were based on expected cessation rates. |
| Relevant findings   \|  \| Nicotine \| Placebo \| Difference (95% CI) \| \| --- \| --- \| --- \| --- \| \| Birthweight (g) \| 3457 \| 3271 \| 186 (35 to 336) \| \| % (n) low birthweight \| 3% (4) \| 9% (11) \| RR 0.4 (0.1 - 1.1) \| \| Birthweight (g) for born after 37 wks gestation \| 3539 \| 3381 \| 157 (25 to 291) \| \| % (n) preterm delivery \| 8% (10) \| 10% (13) \| RR 0.8 (0.4 - 1.7) \| \| Note: Numbers (n) are estimated from %s; Differences and RRs unadjusted it being stated that “Adjustment for preterm delivery, smoking habits and other factors yielded comparable results.” \| \| \| \| | |
| Authors’ main relevant conclusions | “Nicotine patches had no influence on smoking cessation during pregnancy, although they might increase birth weight in comparison with placebo.” |
| Strengths and weaknesses mentioned | Low treatment compliance was noted. |
| Risk of bias | Low |
| Comments | Low treatment compliance, the required number of patches used (out of 77 possible) being only 14 in the nicotine group, and 7 in the placebo group. Among women using all 77 patches, mean birthweight was 509 g (95% CI -149 to 1168 g) higher for those using nicotine patches. Belief that they were given nicotine patches was higher in those given nicotine patches (44%) than in those given placebo patches (11%). |

| **Form No.** | **11** |
| --- | --- |
| Topic | Reproduction/Development – Clinical trial |
| Author(s) | B Kapur et al. (2001) |
| Title | “Randomized, double-blind, placebo-controlled trial of nicotine replacement therapy in pregnancy” |
| Source | Current Therapeutic Research Clinical and Experimental, 2001, 62, 274-278 |
| Study type | Randomized double-blind placebo-controlled trial of smoking cessation. |
| Study location | Toronto, Ontario, Canada |
| Population studied and inclusion criteria | Pregnant women between 12 and 24 weeks’ gestation who smoked ≥15 cigarettes per day and who wanted to quit smoking but could not do so in their first trimester. |
| Nicotine exposures | A daily, 18-hour patch of nicotine, 15 mg for 8 weeks, 10 mg for the next 2 weeks, and 5 mg for the last 2 weeks, or an identical placebo patch. |
| Treatment groups and sizes | 30 women, with 17 allocated to nicotine patches and 13 to placebo. The intended enrolment was 20 patients per group. |
| Relevant endpoints | The study was discontinued because the fetus of the last patient exhibited rapid and forceful movements several hours after a night’s sleep. Obstetrical examination, fetal ultrasound, and a nonstress test were all normal. This is the only relevant endpoint. |
| Confounding variables | No adjustment for confounding variables. There were no significant differences between groups in age, gestational age, various smoking variables, gravidity, parity and bodyweight in pregnancy. |
| Other relevant study details | At baseline, the women attended a counselling session that included a video presentation on how to use the patch. Serum and salivary cotinine levels were measured at baseline and at 1, 4, and 8 weeks; at each of these sessions additional counselling was provided. |
| Relevant findings | \|  \| Subjects with event \| \|  \| \| --- \| --- \| --- \| --- \| \|  \| Nicotine patch \| Placebo patch \| RR \| \| Endpoint \| (N = 17) \| (N = 13) \| (95% CI)^a^ \| \| Rapid and forceful \|  \|  \|  \| \| fetal movements \| 0 \| 1 \| 0.24 (0.01-5.38) \| \|  \|  \|  \|  \| \| ^a^ Estimated \|  \|  \|  \| |
| Authors’ main relevant conclusions | “Although the single adverse case reported in this study does not necessarily prove causation, fetal withdrawal from nicotine and recovery after reintroducing nicotine is a plausible mechanism. This adverse event, and the therapeutic failure of placebo in the study subjects, suggests that future studies in this population should not include a placebo arm.” |
| Strengths and weaknesses mentioned | None |
| Risk of bias | Low |
| Comments | Study of little value. No power calculations. Low power. Conclusion regarding usefulness of placebo controls dubious. |
| **Form No.** | **12** |
| Topic | Reproduction/Development – Clinical trial |
| Author(s) | D R Schroeder et al. (2002) |
| Title | “Nicotine patch use in pregnant smokers: smoking abstinence and delivery outcomes” |
| Source | The Journal of Maternal-Fetal and Neonatal Medicine, 2002, 11, 100-107 |
| Study type | One-sample clinical trial of smoking cessation |
| Study location | Rochester, Minnesota, USA |
| Population studied and inclusion criteria | Women smoking ≥15 cigs/day during their third trimester of pregnancy, despite physician advice to stop smoking.  Inclusion criteria : age ≥18 years; general good health; low-risk pregnancy (outside the smoking risk); ability to participate fully in the study; ability to provide written informed consent.  Exclusion criteria : a recent history of clinically significant heart disease or other medical condition; active chemical dependence on any agent other than nicotine; current psychiatric disorder or use of major psychiatric drugs; history of serious skin allergies; evidence of severe chronic dermatosis; current use of nicotine products other than cigarettes; previous participation in a nicotine patch study; current use of clonidine, busporine, doxepin, or fluoxetine; use of any investigational drug within 30 days. |
| Nicotine exposures | Nicotine patch (22 mg/24 h) initiated during the first day of a 4-day in-hospital study and continued for a total of 8 weeks. |
| Treatment groups and sizes | 21 pregnant women |
| Relevant endpoints | Severe infant morbidity |
| Confounding variables | Not applicable as no comparison group |
| Other relevant study details | Only eight subjects completed all 8 weeks of patch therapy as planned. |
| Relevant findings | One case each of fetal asystole resulting in hypoxic encephalopathy; complete transposition of the great vessels; mild respiratory distress at delivery and what were thought to be neonatal seizures within a month of delivery. |
| Authors’ main relevant conclusions | “Three infants suffered severe neonatal morbidity; however, these problems were unrelated to nicotine patch therapy.” |
| Strengths and weaknesses mentioned | Small sample of pregnancies |
| Risk of bias | High |
| Comments | The lack of a control group renders the study of little value for determining the health effects of NRT. |

| **Form No.** | **13** |
| --- | --- |
| Topic | Reproduction/Development – Clinical trial |
| Author(s) | K I Pollak et al. (2007) |
| Title | “Nicotine replacement and behavioural therapy for smoking cessation in pregnancy” |
| Source | American Journal of Preventive Medicine, 2007, 33, 297-305 |
| Study type | Open-label RCT of smoking cessation |
| Study location | 14 clinical sites in North Carolina, USA |
| Population studied and inclusion criteria | 13 to 25 week pregnant women aged 18+ years who smoked 5+ cigs/day, had smoked 100+ cigs in life, planned to continue prenatal care in one of the clinics, spoke English, and were recruited in 2003 to 2005.  Exclusions : evidence of cognitive or mental health problems; evidence of possible drug or alcohol addiction; documented history of placental abruption, poorly controlled hypertension, cardiac arrhythmia, AMI within the past 6 months, previous pregnancy with congenital anomaly, or family history of congenital anomalies. |
| Nicotine exposures | Women were allocated to cognitive behavioural therapy (CBT) with or without NRT (choice of patch, gum or lozenge). |
| Treatment groups and sizes | 181 women, 59 allocated to CBT only and 122 to CBT + NRT, of which 72 selected patch, 32 gum, 12 lozenge, and 6 no NRT. |
| Relevant endpoints | Birthweight; gestational age; preterm birth; neonatal intensive care admissions; placental abruption; fetal loss; any serious adverse event. |
| Confounding variables | Some analyses were adjusted for a prior history of preterm birth, more common in the CBT + NRT group (32% vs 12%). No notable differences were seen in other baseline characteristics (age, partnered, education, race, employment, site, parity, length of pregnancy, smoking history, and other aspects of pregnancy history). |
| Other relevant study details | Power calculations were based on expected quit rates. An independent Data and Safety Monitoring Board (DSMB) reviewed all SAE reports. Prior to the trial beginning, the DSMB decided that a statistically significant twofold increase in adverse birth outcomes would result in suspension of study enrolment. At a scheduled interim analysis with approximately half of participants enrolled, the DSMB found a twofold difference in SAEs between arms. Because of an a priori stopping rule, they recommended stopping enrolment. The DSMB did state, however, that they did not believe the SAEs were related to NRT use. |
| Relevant findings   \| Endpoint \| NRT + CBT (N = 113) \| CBT only (N = 58) \| Difference (95% CI)^a^ \| \| --- \| --- \| --- \| --- \| \| Mean birthweight (g) \| 3061 (SD 661) \| 3132 (SD 668) \| -71 (-283 to 141) \| \| Mean gestational age \| 37.9 (SD 3.1) \| 38.6 (SD 2.7) \| -0.7 (-1.6 to 0.2) \| \|  \|  \|  \|  \| \| Serious adverse events \|  \|  \| RR (95% CI)^a^ \| \| 1) Unadjusted analyses \|  \|  \|  \| \| All \| 30.1% (34) \| 17.2% (10) \| 1.75 (0.93-3.28) \| \| Pre-eclampsia \| 2.7% (3) \| 3.4% (2) \| 0.77 (0.13-4.48) \| \| Placental abnormality \| 3.5% (4) \| 0.0% (0) \| 4.64 (0.25-84.7) \| \| Preterm birth \| 21.2% (24) \| 15.5% (9) \| 1.37 (0.68-2.75) \| \| Small for gestational age \| 3.5% (4) \| 0.0% (0) \| 4.64 (0.25-84.7) \| \| Neonatal intensive care admission \| 13.3% (15) \| 5.2% (3) \| 2.57 (0.77-8.51) \| \| Fetal loss \| 2.7% (3) \| 3.4% (2) \| 0.77(0.13-4.48) \| \|  \|  \|  \|  \| \| 2)Analysis adjusted for history of preterm birth \| \|  \|  \| \| All \| 27% \| 18% \| 1.50 (0.80-2.81) \| \|  \|  \|  \|  \| \| ^a^ Estimated, unadjusted \|  \|  \|  \| \|  \|  \|  \|  \| | |
| Authors’ main relevant conclusions | “Recruitment was suspended early by an independent Data and Safety Monitoring Board when an interim analysis found a greater rate of negative birth outcomes in the CBT+NRT arm than in the CBT arm. At the final analysis the difference between the arms in rate of negative birth outcomes was 0.09 (p=0.26), adjusted for prior history of preterm birth. More data are needed to determine the safety and to confirm the efficacy of NRT use during pregnancy.” |
| Strengths and weaknesses mentioned | Various limitations noted including : low power to detect differences in specific pregnancy outcomes; randomization should have been stratified on history of negative pregnancy outcomes; women lost to follow-up were non-random; high loss to follow-up rates. |
| Risk of bias | UNCLEAR |
| Comments | None |

| **Form No.** | **14** |
| --- | --- |
| Topic | Reproduction/Development – Clinical trial |
| Author(s) | C Oncken et al. (2008) |
| Title | “Nicotine gum for pregnant smokers: a randomized controlled trial” |
| Source | Obstetrics and Gynecology, 2008, 112, 859-867 |
| Study type | Placebo-controlled RCT of smoking cessation |
| Study location | Three sites in Connecticut and Maine, USA |
| Population studied and inclusion criteria | Pregnant women of age 16+, smoking 1 cig/day, of up to 26 weeks gestation, able to speak English or Spanish; intending to carry their pregnancy to term; and living in a stable residence.  Exclusion criteria : evidence of current drug or alcohol use disorder; twins or other multiple gestation; an unstable psychiatric medical problem; a medical problem that would interfere with study participation. |
| Nicotine exposures | Nicotine 2 mg or placebo gum. Asked to chew one piece of gum per cigarette usually smoked. |
| Treatment groups and sizes | 194 women, 100 allocated to gum and 94 to placebo. |
| Relevant endpoints | Birthweight; gestational age; infant length and head circumference; serious adverse events (including maternal hospitalization, low birthweight, very low birthweight, spontaneous abortion, fetal loss, NICU admission, new born death, any serious adverse event). |
| Confounding variables | No adjustment for confounding variables. The groups were comparable on age, race, education, insurance, methadone and antidepressant use, treatment history, smoking and pregnancy history. |
| Other relevant study details | Power calculations were based on expected cessation rates. However, enrolment stopped after 194 of planned 268 due to lack of efficacy in smoking cessation. The study population consisted primarily of socioeconomically disadvantaged women, including some who were being treated for mental health problems or who had a history of a substance use disorder. |
| Relevant findings   \|  \| NRT  Mean (SD, N) \| Placebo  Mean (SD, N) \| Difference  Mean (95% CI)^a^ \| \| --- \| --- \| --- \| --- \| \| Birthweight \| 3287 (566, 93) \| 2950 (653, 84) \| 337.0 (154.8 to 519.2) \| \| Gestational age \| 38.9 (1.7, 93) \| 38 (3.3, 84) \| 0.9 (0.1 to 1.7) \| \| Infant length \| 50 (2.7, 92) \| 49 (4.4, 80) \| 1.0 (-0.1 to 2.1) \| \| Head circumference \| 34 (1.7, 90) \| 33.5 (2, 72) \| 0.5 (-0.1 to 1.1) \| \|  \|  \|  \|  \| \|  \| Median (N) \| Median (N) \| p value \| \| Apgar Score 1 minute \| 8 (93) \| 8 (84) \| 0.62 \| \| Apgar Score 5 minutes \| 9 (93) \| 9 (84) \| 0.061 \| \|  \|  \|  \|  \| \|  \| % (n)^b^ \| % (n)^c^ \| RR (95% CI)^a^ \| \| Maternal hospitalization \| 9.3 (9) \| 9.2 (8) \| 1.01 (0.41 - 2.50) \| \| Low birthweight \| 2.1 (2) \| 18.4 (16) \| 0.11 (0.03 - 0.47) \| \| Very low birthweight \| 1.0 (1) \| 4.6 (4) \| 0.22 (0.03 - 1.97) \| \| Preterm delivery \| 7.2 (7) \| 18.4 (16) \| 0.39 (0.17 - 0.91) \| \| Spontaneous abortion \| 2.1 (2) \| 0.0 (0) \| 4.49 (0.22 - 92.19) \| \| Fetal loss \| 2.1 (2) \| 1.1 (1) \| 1.79 (0.17 - 19.44) \| \| NICU admission \| 7.2 (7) \| 12.6 (11) \| 0.57 (0.23 - 1.41) \| \| New born death \| 1.0 (1) \| 2.3 (2) \| 0.45 (0.04 - 4.86) \| \| Any serious adverse event \| 24.7 (24) \| 37.9 (33) \| 0.65 (0.42 - 1.01) \| \| ^a^ Estimated, unadjusted ^b^ N = 97 ^c^ N = 87 \| \| \|  \| | |
| Authors’ main relevant conclusions | “There were clinically important and statistically significant differences in favour of NRT in birth weight and gestational age. There were non-significant differences favouring NRT for infant length, head circumference, and Apgar score at 5 minutes.” |
| Strengths and weaknesses mentioned | None |
| Risk of bias | Low |
| Comments |  |

| **Form No.** | **15** |
| --- | --- |
| Topic | Reproduction/Development – Clinical trial |
| Author(s) | G K Swamy et al. (2009) |
| Title | “Predictors of adverse events among pregnant smokers exposed in a nicotine replacement therapy trial” |
| Source | American Journal of Obstetrics and Gynecology, 2009, 201, 1-11 |
| Study type | Open-label RCT of smoking cessation |
| Study location | 14 clinical sites in North Carolina, USA |
| Population studied and inclusion criteria | 13 to 25 week pregnant women aged 18+ years who smoked 5+ cigs/day, had smoked 100+ cigs in life, planned to continue prenatal care in one of the clinics, spoke English, and were recruited in 2003 to 2005.  Exclusions : evidence of cognitive or mental health problems; evidence of possible drug or alcohol addiction; documented history of placental abruption, poorly controlled hypertension, cardiac arrhythmia, AMI within the past 6 months, previous pregnancy with congenital anomaly, or family history of congenital anomalies. |
| Nicotine exposures | Women were allocated to cognitive behavioural therapy (CBT) with or without NRT (choice of patch, gum or lozenge). |
| Treatment groups and sizes | Originally (see CAF 12, p. A25) the trial involved 181 women, 59 allocated to CBT only and 122 to CBT + NRT, of which 72 selected patch, 32 gum, 12 lozenge, and 6 no NRT. The analyses described in this publication involved review of medical records from 157 women, 52 allocated to CBT only and 105 to CBT + NRT. |
| Relevant endpoints | Any serious adverse event; pre-eclampsia; low birthweight; placental abnormalities. |
| Confounding variables | The final analysis simultaneously related serious adverse events to 10 predictors : randomization arm; maternal age; education; race; baseline stress; baseline depression score; adequate prenatal care index; history of depression; analgesic medication use during pregnancy; and adverse pregnancy history. Other predictors considered, but not included in the trial model were : employment; partnered; obstetric history; history of anxiety disorder; history of drug or alcohol use; history of sexually transmitted infection; baseline cigs/day; baseline self-esteem; baseline coping. |
| Other relevant study details | This was an additional analysis of the data described in CAF 13 with the stated objective “To determine the contribution of randomization to nicotine replacement therapy (NRT), socio-demographic and psychosocial factors, and pregnancy and medical history to serious perinatal adverse events among pregnant smokers.” |
| Relevant findings   \|  \| NRT + CBT \| CBT \|  \| \| --- \| --- \| --- \| --- \| \| Endpoint \| % (n)^a^ \| % (n)^b^ \| RR (95% CI) \| \| All serious adverse events \| 31.4 (33) \| 17.3 (9) \| 1.82 (0.94-3.51)^c^ \| \| - adjusted \|  \|  \| 2.38 (0.91-6.25) \| \| Pre-eclampsia \| 2.9 (3) \| 3.8 (2) \| 0.74 (0.13-4.31)^c^ \| \| Low birthweight \| 3.8 (4) \| 0.0 (0) \| 4.48 (0.25-81.63)^c^ \| \| Placental abnormalities \| 3.8 (4) \| 0.0 (0) \| 4.48 (0.25-81.63)^c^ \| \|  \|  \|  \|  \| \| ^a^ N = 105 ^b^ N = 52 ^c^ Estimated \| \| \|  \| \|  \|  \|  \|  \| | |
| Authors’ main relevant conclusions | “While race, poor pregnancy history, and use of analgesics were associated with serious adverse events, randomization to NRT during pregnancy was not a significant factor.” |
| Strengths and weaknesses mentioned | Discussed in CAF 13. |
| Risk of bias | UNCLEAR |
| Comments | Only limited results given for specific serious adverse events. |

| **Form No.** | **16** |
| --- | --- |
| Topic | Reproduction/Development – Clinical trial |
| Author(s) | T Coleman et al. (2012) |
| Title | “A randomized trial of nicotine-replacement therapy patches in pregnancy” |
| Source | The New England Journal of Medicine, 2012, 366, 808-818 |
| Study type | Double-blind placebo-controlled RCT of smoking cessation |
| Study location | Seven hospitals in Midlands and North-West England |
| Population studied and inclusion criteria | Pregnant women were recruited between 2007 and 2010 who agreed to set a quit date, were 16 to 50 years of age, were at 12 to 24 weeks of gestation, smoked 10+ cigs daily before pregnancy, currently smoked 5+ cigs daily, and had an exhaled carbon monoxide concentration of at least 8 ppm.  Exclusion criteria : major fetal abnormalities; inability to provide informed consent; chemical or alcohol dependence; contraindications to nicotine-replacement therapy. |
| Nicotine exposures | Nicotine patches (15 mg per 16 hours) or placebo patches. |
| Treatment groups and sizes | 1050 women, 521 allocated to NRT and 529 to placebo. |
| Relevant endpoints | These include : miscarriage, stillbirth, neonatal death, postneonatal death, birthweight, gestational age, preterm birth, low birthweight, NICU admission, Apgar score, total congenital abnormalities, serious adverse events; pre-eclampsia or eclampsia, hospital admission for pregnancy complication. |
| Confounding variables | No adjustment for confounding variables (except recruitment centre). The groups were similar as regards age, race, gestational age, age leaving education, parity, height, weight, various aspects of smoking, previous preterm birth, previous NRT use in pregnancy and length of last behavioural support session. |
| Other relevant study details | Power calculations based on expected quit rates.  Compliance was low; only 7.2% of women assigned to nicotine-replacement therapy and 2.8% assigned to placebo used patches for more than 1 month. |
| Relevant findings   \|  \| NRT \| \| \|  \| \| Placebo \| \| \| \|  \| \| \| --- \| --- \| --- \| --- \| --- \| --- \| --- \| --- \| --- \| --- \| --- \| --- \| \| Endpoint \| N \| % (n) \| \|  \| \| N \| \| % (n) \| \| OR (95% CI)^a^ \| \| \| Miscarriage \| 515 \| 0.6 (3) \| \|  \| \| 521 \| \| 0.4 (2) \| \| 1.52 (0.25-9.13) \| \| \| Stillbirth \| 512 \| 1.0 (5) \| \|  \| \| 519 \| \| 0.4 (2) \| \| 2.59 (0.50-13.4) \| \| \| Neonatal death \| 507 \| 0.0 (0) \| \|  \| \| 517 \| \| 0.4 (2) \| \| 0.20 (0.01-4.24)^b^ \| \| \| Postneonatal death \| 507 \| 0.2 (1) \| \|  \| \| 517 \| \| 0.0 (0) \| \| 3.07 (0.12-75.4)^b^ \| \| \| Preterm birth \| 507 \| 7.9 (40) \| \|  \| \| 517 \| \| 8.7 (45) \| \| 0.90 (0.58-1.41) \| \| \| Low birthweight \| 507 \| 11.0 (56) \| \|  \| \| 517 \| \| 8.3 (43) \| \| 1.38 (0.90-2.09) \| \| \| NICU admission \| 507 \| 6.5 (33) \| \|  \| \| 517 \| \| 6.8 (35) \| \| 0.96 (0.58-1.57) \| \| \| Apgar score <7 at 5 mins \| 507 \| 3.2 (16) \| \|  \| \| 517 \| \| 3.5 (18) \| \| 0.91 (0.45-1.80) \| \| \| Total congenital abnormalities^b^ \| 507 \| 1.8 (9) \| \|  \| \| 517 \| \| 2.5 (13) \| \| 0.70 (0.30-1.66) \| \| \| Serious adverse events \| 521 \| 1.7 (9) \| \|  \| \| 529 \| \| 1.1 (6) \| \| 1.53 (0.54-4.34)^b^ \| \| \| Pre-eclampsia or eclampsia \| 521 \| 0.6 (3) \| \|  \| \| 529 \| \| 0.9 (5) \| \| 0.61 (0.14-2.55)^b^ \| \| \| Hospital admission for pregnancy complication \| 521 \| 8.4 (44) \| \|  \| \| 529 \| \| 7.8 (41) \| \| 1.10 (0.70-1.71)^b^ \| \| \|  \| N \| Mean (SD) \| \|  \| \| N \| \| Mean (SD) \| \| Mean difference (95% CI)^c^ \| \| \| Birthweight unadjusted \| 507 \| 3.18 (0.61) \| \|  \| \| 507 \| \| 3.20 (0.59) \| \| -0.02 (-0.10 to 0.05) \| \| \| Birthweight score \| 507 \| -0.36 (0.99) \| \|  \| \| 507 \| \| -0.31 (1.02) \| \| -0.05 (-0.17 to 0.08) \| \| \| Gestational age \| 507 \| 39.5 (2.1) \| \|  \| \| 507 \| \| 39.5 (2.1) \| \| 0.0 (-0.2 to 0.3) \| \| \|  \|  \|  \| \|  \| \|  \| \|  \| \|  \| \| \| ^a^ Adjusted for recruitment centre ^b^ No specific abnormality was seen in more than two infants in either group ^c^ Estimated (unadjusted) \| \| \| \| \| \| \| \| \| \| \| \| \|  \|  \| \|  \| \|  \| \|  \| \|  \| \|  \| | |
| Authors’ main relevant conclusions | “Adding a nicotine patch (15 mg per 16 hours) to behavioral cessation support for women who smoked during pregnancy did not significantly increase the rate of abstinence from smoking until delivery or the risk of adverse pregnancy or birth outcomes. However, low compliance rates substantially limited the assessment of safety.” |
| Strengths and weaknesses mentioned | “The trial was four times as large as the largest, previous similar study.”  “The low adherence rates for nicotine-replacement therapy and the fact that a much larger sample would be required to comprehensively assess the effect of this therapy on infrequent adverse birth outcomes.” |
| Risk of bias | LOW |
| Comments | None |

| **Form No.** | **17** |
| --- | --- |
| Topic | Reproduction/Development – Clinical trial |
| Author(s) | A A E El-Mohandes et al. (2013) |
| Title | “A randomized clinical trial of trans-dermal nicotine replacement in pregnant African-American smokers” |
| Source | Maternal Child Health, 2013, 17, 897-906 |
| Study type | Placebo-controlled RCT of smoking cessation |
| Study location | Washington DC, USA |
| Population studied and inclusion criteria | <30 week pregnant smokers aged 18+ with a desire to quit, who were English speaking, DC metropolitan area residents, and self-identified as an ethnic minority and recruited in 2006-2009. Women were eligible if their CO levels were ≥8 ppm, salivary cotinine levels were ≥20 ng/ml, or urinary cotinine levels were ≥100 ng/ml. Women under treatment for psychiatric illness, alcoholism or drug addiction were excluded. |
| Nicotine exposures | Women were allocated to cognitive behavioural therapy (CBT) with or without nicotine patches. Women randomized to the patch plus CBT were classified into two dosing groups based on baseline salivary cotinine levels (SCL). Women with a SCL ≥ 100 ng/ml received 21 mg patches for 2 weeks, 14 mg patches for 4 weeks, and 7 mg patches for 4 weeks. Women with a baseline SCL ≥ 20 and ≤100 ng/ml received 14 mg patches for 6 weeks and 7 mg patches for 4 weeks. |
| Treatment groups and sizes | 52 women, 26 allocated to each group, though one woman allocated to NRT + CBT did not actually receive NRT. |
| Relevant endpoints | Gestational age; birthweight; preterm birth. |
| Confounding variables | No adjustment for confounding variables. The groups did not differ significantly on maternal age, gestational age at baseline, number of pregnancies, number of live births, relationship status, educational level, employment status, Medicaid, alcohol use during pregnancy, depressive symptoms, marijuana during pregnancy, or various aspects of smoking. |
| Other relevant study details | Power calculations were based on expected quit rates. |
| Relevant findings | \|  \| NRT + CBT \| CBT only \|  \| \| --- \| --- \| --- \| --- \| \|  \| (mean) \| (mean) \| Difference (95% CI) \| \| Gestational age \| 39.4 \| 38.4 \| 1.0 (0.16 to 1.84)^a^ \| \| Birthweight (g) \| 3203 \| 2997 \| 206 (- 98 to 510)^a^ \| \|  \|  \|  \|  \| \|  \| % (n) \| % (n) \| RR (95% CI) \| \| Preterm birth \| 4% (1) \| 8% (2) \| 0.50 (0.05-5.18)^a^ \| \| Low birthweight \| 12% (3) \| 16% (4) \| 0.75 (0.19-3.03)^a^ \| \|  \|  \|  \|  \| \| ^a^ Estimated, unadjusted \| \|  \|  \| \|  \|  \|  \|  \| |
| Authors’ main relevant conclusions | “A significant increase in gestational age of 1 week was observed for the NRT plus CBT group.” |
| Strengths and weaknesses mentioned | Sample size too small to evaluate intervention impact. |
| Risk of bias | LOW |
| Comments | None |

| **Form No.** | **18** |
| --- | --- |
| Topic | Reproduction/Development – Clinical trial |
| Author(s) | I Berlin et al. (2014) |
| Title | “Nicotine patches in pregnant smokers: randomised, placebo controlled, multicentre trial of efficacy” |
| Source | British Medical Journal, 2014, 348, g1622 |
| Study type | Double-blind placebo-controlled randomized multicentre clinical trial of smoking cessation |
| Study location | 23 maternity wards throughout France |
| Population studied and inclusion criteria | Pregnant smokers aged 18+ with a gestational age of between nine and 20 weeks who smoked at least five cigarettes/day and scored at least 5 on motivational scale of quitting (range 0-10). Participants had to be affiliated with a health insurance system.  Exclusion criteria : refusal to use patches; use of neuroleptics, antidepressants, or anxiolytics for a chronic psychiatric disorder; a skin disorder contraindicating use of patches; use of tobacco product other than cigarettes; current and previous month’s use of any NRT; or use of bupropion or varenicline. |
| Nicotine exposures | 16 hour delivery nicotine patches (10 or 5 mg dependent on cotinine level) or placebo patches. |
| Treatment groups and sizes | 402 women, 203 allocated to nicotine and 199 to placebo patches. Data were available on 192 live births in each group. |
| Relevant endpoints | Birthweight, fetal growth restriction, low birthweight, length at birth, head circumference, Apgar score <10 at 5 mins, gestational age, preterm birth, transfer to neonatal intensive care unit, serious adverse event, stillbirth, total fetal death, congenital malformation, pre-eclampsia. |
| Confounding variables | No adjustment for confounding variables. No significant difference between groups in age, professional status, marital status, annual household income, ethnic origin, blood pressure, body mass index, gestational age, parity, number of previous pregnancies, history of premature delivery, history of small for gestational age at birth, maternal disorders before randomization, various aspects of smoking, drinking, exposure to secondhand smoke, cannabis use, prescribed drug use, first nicotine or placebo patch dose. |
| Other relevant study details | Power calculations were based on expected quit rates. |
| Relevant findings   \|  \| Nicotine patch \| Placebo patch \|  \| \| --- \| --- \| --- \| --- \| \|  \| (N = 192) \| (N = 192) \|  \| \| Endpoint \| Mean (SE) \| Mean (SE) \| Difference (95% CI)^a^ \| \| Birthweight (g) \| 3065 (44) \| 3015 (44) \| 50 (-71.1 to 172.3) \| \| Length at birth (cm) \| 48 3 (0.23) \| 48.0 (0.23) \| 0.34 (-0.31 to 0.98) \| \| Head circumference (cm) \| 33.7 (0.16) \| 33.9 (0.16) \| -0.2 (-0.63 to 0.24) \| \| Gestational age (weeks) \| 38.3 (3.1) \| 38.5 (2.99) \| -0.22 (-0.82 to 0.38) \| \|  \|  \|  \|  \| \|  \| % (n) \| % (n) \| RR (95% CI)^a,b^ \| \| Fetal growth restriction \| 17.7 (34) \| 24.0 (46) \| 0.74 (0.50-1.10) \| \| Low birthweight \| 14.1 (27) \| 17.2 (33) \| 0.82 (0.51-1.31) \| \| Apgar score <10 at 5 mins \| 6.8 (13) \| 5.7 (11) \| 1.18 (0.54-2.57) \| \| Preterm birth \| 13.5 (27) \| 13.1 (26) \| 1.03 (0.63-1.71) \| \| Transfer to NICU \| 6.0 (12) \| 7.0 (14) \| 0.85 (0.40-1.80) \| \| Serious adverse event – mother \| 11.8 (24) \| 8.0 (16) \| 1.47 (0.81-2.68) \| \| Serious adverse event – newborn/fetus \| 6.8 (13) \| 7.3 (14) \| 0.93 (0.45-1.92) \| \| Stillbirth \| 2.0 (4) \| 2.5 (5) \| 0.80 (0.22-2.94) \| \| Total fetal death \| 3.9 (8) \| 3.4 (7) \| 1.14 (0.42-3.09) \| \| Congenital malformation \| 2.0 (4) \| 3.0 (6) \| 0.67 (0.19-2.33) \| \| Pre-eclampsia \| 1.5 (3) \| 0.5 (1) \| 3.00 (0.31-28.60) \| \| ^a^  Unadjusted ^b^  Estimated from data provided \| \| \| \| | |
|  |  |
| Authors’ main relevant conclusions | “The frequency of serious adverse events was similar between the groups.”  “The nicotine patch did not increase either smoking cessation rates or birth weights despite adjustment of nicotine dose to match levels attained when smoking, and higher than usual doses.” |
| Strengths and weaknesses mentioned | Strengths : individualised adjustment of daily nicotine dose according to saliva cotinine levels while smoking, potentially resulting in a close to 100% nicotine substitution rate. Compared with previous studies the duration of treatment was longer and higher daily doses of nicotine were administered, leading to an overall higher exposure to nicotine. The self-reported compliance rate was higher than previously reported probably because of the relatively frequent face to face visits. The placebo patches were manufactured by the same company, with specific quality control guidelines to ensure double blinding.  Weaknesses : treatment started only after the end of the first trimester. The trial’s population was a highly tobacco dependent group. Generalisability of the results to a less dependent population of pregnant smokers should be done with caution. |
| Risk of bias | LOW |
| Comments | None |

| **Form No.** | **19** |
| --- | --- |
| Topic | Reproduction/Development – Clinical trial |
| Author(s) | S Cooper et al. (2014a) |
| Title | “The SNAP trial: a randomised placebo-controlled trial of nicotine replacement therapy in pregnancy – clinical effectiveness and safety until 2 years after delivery, with economic evaluation” |
| Source | Health Technology Assessment , 2014, 18, 1-128 |
| Study type | Double-blind placebo-controlled RCT of smoking cessation |
| Study location | Seven hospitals in the Midlands and north-west England |
| Population studied and inclusion criteria | Pregnant women were recruited between 2007 and 2010 who agreed to set a quit date, were 16 to 50 years of age, were at 12 to 24 weeks of gestation, smoked 10+ cigs daily before pregnancy, currently smoked 5+ cigs daily, and had an exhaled carbon monoxide concentration of at least 8 ppm.  Exclusion criteria : major fetal abnormalities; inability to provide informed consent; chemical or alcohol dependence; contraindications to nicotine-replacement therapy. |
| Nicotine exposures | Nicotine patches (15 mg per 16 hours) or placebo patches. |
| Treatment groups and sizes | 1050 women, 521 allocated to NRT and 529 to placebo. |
| Relevant endpoints | Results cited are all from CAF 16 or CAF 20 and are not repeated here. |
| Confounding variables | No adjustment for confounding variables (except recruitment centre). The groups were similar as regards age, race, gestational age, age leaving education, parity, height, weight, various aspects of smoking, previous preterm birth, previous NRT use in pregnancy and length of last behavioural support session. |
| Other relevant study details | Power calculations based on expected quit rates.  Compliance was low; only 7.2% of women assigned to nicotine-replacement therapy and 2.8% assigned to placebo used patches for more than 1 month.  This publication is a very detailed report on the SNAP trial, but gives no new relevant results other than those cited in CAF 16 and CAF 20. |
| Relevant findings | See CAF 16 and CAF 20. |
| Authors’ main relevant conclusions | “There was no evidence for NRT having either a beneficial or a harmful effect on birth outcomes, apart from slightly higher caesarean rates in the NRT group. However, as adherence was poor, birth outcome findings are difficult to interpret and could have been different had greater adherence with trial treatments occurred.  At 2 years, infants born to participants randomized to NRT were more likely to have survived without any impairment, but there were no significant differences in infants’ respiratory problems. The most likely reason for better NRT group infant outcomes are the lower, albeit largely non-significant, smoking rates in NRT group mothers.” |
| Strengths and weaknesses mentioned | Strengths : Large sample size; first trial to test effect of smoking cessation intervention in pregnancy on infant outcomes; long (2 year) follow-up after birth; double-blind design.  Weaknesses : Incomplete smoking data post randomization; no questions on perception of allocation. |
| Risk of bias | LOW |
| Comments | None |

| **Form No.** | **20** |
| --- | --- |
| Topic | Reproduction/Development – Clinical trial |
| Author(s) | S Cooper et al. (2014b) |
| Title | “Effect of nicotine patches in pregnancy on infant and maternal outcomes at 2 years: follow-up from the randomised, double-blind, placebo-controlled SNAP trial” |
| Source | Lancet Respiratory Medicine, 2014, 2, 728-737 |
| Study type | Double-blind placebo-controlled RCT of smoking cessation |
| Study location | Seven hospitals in Midlands and North-West England |
| Population studied and inclusion criteria | Pregnant women were recruited between 2007 and 2010 who agreed to set a quit date, were 16 to 50 years of age, were at 12 to 24 weeks of gestation, smoked 10+ cigs daily before pregnancy, currently smoked 5+ cigs daily, and had an exhaled carbon monoxide concentration of at least 8 ppm.  Exclusion criteria : major fetal abnormalities; inability to provide informed consent; chemical or alcohol dependence; contraindications to nicotine-replacement therapy. |
| Nicotine exposures | Nicotine patches (15 mg per 16 hours) or placebo patches. |
| Treatment groups and sizes | Initially, 521 women were allocated to NRT and 529 to placebo. Of the 1010 live singleton births, 503 were allocated to NRT and 507 to placebo. Developmental outcome data were available for 445 infants in the NRT group and 443 in the placebo group. |
| Relevant endpoints | Infant deaths in first two years after birth; definite or suspected developmental impairment by two years after birth; documented respiratory outcomes by two years after birth. |
| Confounding variables | No adjustment for confounding variables (except recruitment centre). The groups were similar as regards age, race, gestational age, age leaving education, parity, height, weight, various aspects of smoking, previous preterm birth, previous NRT use in pregnancy and length of last behavioural support session. |
| Other relevant study details | Power calculations based on expected quit rates.  Compliance was low; only 7.2% of women assigned to nicotine-replacement therapy and 2.8% assigned to placebo used patches for more than 1 month. |
| Relevant findings   \|  \| NRT \| \| Placebo \| \|  \| \| --- \| --- \| --- \| --- \| --- \| --- \| \| Endpoint \| N \| % (n) \| N \| % (n) \| OR (95% CI)^a^ \| \| Infant death \| 445 \| 0.4 (2) \| 443 \| 0.4 (2) \| 1.00 (0.14-7.10)^b^ \| \| Definite developmental impairment^c^ \| 445 \| 10.8 (48) \| 443 \| 12.2 (64) \| 0.71 (0.48-1.06) \| \| Survival with no impairment^d^ \| 445 \| 72.6 (323) \| 443 \| 65.5 (290) \| 1.41 (1.05-1.87) \| \| Respiratory problems \| 444 \| 19.7 (132) \| 444 \| 25.0 (111) \| 1.28 (0.95-1.73) \| \|  \|  \|  \|  \|  \|  \| \| ^a^ Adjusted for recruitment centre ^b^ Estimated (unadjusted) ^c^ Excluding deaths ^d^ Definite or suspected \| \| \| \| \| \| \|  \|  \|  \|  \|  \|  \| | |
| Authors’ main relevant conclusions | “Infants born to women who used NRT for smoking cessation in pregnancy were more likely to have unimpaired development.” |
| Strengths and weaknesses mentioned | Strengths : High outcome ascertainment rates; outcomes checked by medically qualified researcher.  Weaknesses : No good data on smoking or ETS exposure after randomization. |
| Risk of bias | LOW |
| Comments | Similar ORs estimated using complete case analysis (adjusting for clustering by twin pregnancies) and using multiple computation intention-to-treat analyses. |

| **Form No.** | **21** |
| --- | --- |
| Topic | CVD – Epidemiology |
| Author(s) | S E Kimmel et al. (2001) |
| Title | “Risk of acute first myocardial infarction and use of nicotine patches in a general population” |
| Source | Journal of the American College of Cardiology, 2001, 37, 1297-1302 |
| Study type | Case-control study |
| Study location | 68 acute-care hospitals in the Philadelphia metropolitan area, Pennsylvania, USA |
| Population studied and inclusion criteria | Cases were smokers (current or within the prior year) admitted with a first MI which was not a complication of a hospitalization for a different condition, and hospitalized in September 1995 to December 31 1997. Controls were smokers (similarly defined) without prior MI, selected by random-digit dialling. All participants had to have a telephone, speak English, not be pregnant or nursing, and live in one of the eight counties where the hospitals were situated. |
| Nicotine exposures | The primary definition used was any nicotine patch use within one week of the index date (date of MI for cases or date of telephone interview for controls). Additional analyses related to one day before the index date, and to exposure to any NRT. |
| Treatment groups and sizes | 653 cases of first MI and 2990 controls. |
| Relevant endpoints | All AMI, AMI confirmed by chart review. |
| Confounding variables | Age (≥50 yrs), BMI ≥25.2 kg/m^2^), cigarettes (≥19 pack-years), diabetes, family history of CHD, sex (male), history of angina or coronary disease, history of high cholesterol and hypertension, all factors being significantly more frequent in cases, and also family income (<$30,000/yr), private health insurance and race. |
| Other relevant study details | The study was designed to have an 80% power to detect an OR of 2.5 (equal to that of smoking itself) as significant at p < 0.05. Participation rates were 68% for cases and 51% for controls. |
| Relevant findings |  |
| \|  \|  \|  \| Cases \| \|  \| Controls \| \|  \|  \| \| --- \| --- \| --- \| --- \| --- \| --- \| --- \| --- \| --- \| --- \| \| Endpoint \| Exposure \|  \| Exp. \| Unexp. \|  \| Exp. \| Unexp. \|  \| OR (95% CI) \| \|  \|  \|  \|  \|  \|  \|  \|  \|  \|  \| \| Any MI \| Patch in last week \|  \| 3 \| 650 \|  \| 30 \| 2960 \|  \| 0.46 (0.09-1.47) \| \| (Among smokers on day used patch) \|  \|  \|  \|  \|  \|  \|  \|  \| 0.83 (0.09-3.81) \| \| (Among smokers smoking in index week) \|  \|  \|  \|  \|  \|  \|  \|  \| 0.38 (0.04-1.54) \| \| (Among nonsmokers in index week) \|  \|  \|  \|  \|  \|  \|  \|  \| 0.25 (0.01-1.67) \| \| Confirmed MI \| Patch in last week \|  \| 3 \| 502 \|  \| 30 \| 2960 \|  \| 0.59 (0.11-1.91) \| \| Any AMI \| Any NRT last week \|  \|  \|  \|  \|  \|  \|  \| 0.47 (0.15-1.19) \| \| Any AMI \| Patch in last day \|  \|  \|  \|  \|  \|  \|  \| 0.59 (0.11-1.98) \| \|  \|  \|  \|  \|  \|  \|  \|  \|  \|  \| \| Analyses were also presented adjusted for each of the individual potential confounders with the OR varying from 0.32 (adjusted for family income) to 0.5 (adjusted for family history of coronary disease) \| \| \| \| \| \| \| \| \| \| | |
| Authors’ main relevant conclusions | “Nicotine patches, as used in actual practice, do not appear to be associated with an increased risk of MI.”  “It is riskier to continue to smoke than to use the nicotine patch.”  “These results add further support to the safety of nicotine patches when users follow the recommended guidelines and abstain from cigarette smoking during patch use.” |
| Strengths and weaknesses mentioned | Strengths : 1. Population-based. 2. Study reflects real-life use of the patch. 3. Study large enough to evaluate a risk from patches equivalent to that from smoking.  Weaknesses : 1. Few exposed cases rendering full confounder adjustment impossible. 2. Non-participation bias. 3. Recall bias. 4. Exclusion of persons dying of cardiac death. |
| Study quality score | FAIR |
| Comments | While the study is large and well conducted, the small number of cases using patches does not rule out a possible modest adverse effect of NRT. However, if nicotine were the major cause of the observed increased risk of AMI in smokers one would expect to have seen a positive association with NRT. |

| **Form No.** | **22A** |
| --- | --- |
| Topic | CVD – Epidemiology |
| Author(s) | R Hubbard et al. (2005) |
| Title | “Use of nicotine replacement therapy and the risk of acute myocardial infarction, stroke, and death” |
| Source | Tobacco Control, 2005, 14, 416-421 |
| Study type | Case series analysis based on a national database |
| Study location | UK |
| Population studied and inclusion criteria | Patients with at least one prescription for NRT between June 1985 and November 2003. |
| Nicotine exposures | 33247 individuals were prescribed NRT. Comparisons are made of disease incidence in the 56 days before prescription and in the 56 days after. Analyses are presented for all NRT users and separately for patch users, gum/lozenge/microtablet users and inhalers/nasal spray users. |
| Treatment groups and sizes | Of the 33247 studied, 25596 (77%) were prescribed patch, 5421 (16%) gum, lozenge or microtablets, 1614 (5%) inhalers, and 616 (2%) nasal spray. Of the patch users, 18065 (71%) were prescribed a high dose (≥15mg) patch, 6025 (24%) a medium dose (10-14 mg) patch, and 1506 (6%) a low dose (<10 mg) patch. Of the gum users 995 (27%) were given 4 mg gum and 2648 (73%) 2 mg gum. |
| Relevant endpoints | AMI, death (note that death rates after prescription were compared to the expected level). |
| Confounding variables | Age at time of MI. |
| Other relevant study details | The authors “estimated that with 350 cases of myocardial infarction exposed to NRT, we would have more than 90% power to detect a rate ratio of 1.5 or greater”. Incidence for the four 14 day periods before prescription and the four after prescription were compared relative to that in the remaining, baseline, time periods, using conditional Poisson regression. |
| Relevant findings | 861 NRT users had at least one diagnosis of MI, of whom 146 had a second and 27 a third. Of the 861 MI diagnoses, 752 occurred outside the period considered for analysis, with 431 being more than 56 days before NRT, 11 on the day of the prescription and 310 more than 56 days after NRT. Relative incidence compared to baseline levels was 5.55 (95% CI 4.42-6.98) in the 56 days before NRT, based on 88 cases, and 1.27 (0.82-1.97) in the 56 days after, based on 21 cases. Incidence ratios “increased progressively in the four 14-day periods before prescription (3.29, 6.02, 4.48, 8.51) and “fell progressively” after (2.39, 0.97, 1.47, 0.24). There was no evidence of effect modification by age or sex, but the incidence ratio before NRT was higher in those with no previous history of angina. There was no evidence of an increased risk after prescription for any particular NRT formulation. Results were similar for second MI.  In the 56 days after NRT there were 33 deaths, somewhat less than expected (0.86 95% CI 0.60-1.23). |
| Authors’ main relevant conclusions | “The use of NRT is not associated with any increase in the risk of myocardial infarction, stroke, or death.”  The increased incidence of MI before the first NRT prescription suggests “that NRT is currently being prescribed shortly after myocardial infarctions and strokes.” “Theoretical concerns over the safety of NRT in relation to these adverse outcomes can therefore probably be discounted, particularly when the alternative for most smokers is to continue to smoke.” |
| Strengths and weaknesses mentioned | “The main strengths of our study are its large size, the long duration of patient follow-up, and the detailed information available on prescriptions. The potential weaknesses of the study are the validity of the outcome data, the extent to which people prescribed NRT actually used the treatment, and the use of NRT purchased without a prescription over the counter.”  We “have excluded the effects of the major likely confounders by using the self controlled case series method.” |
| Study quality score | FAIR |
| Comments | The study reports no data on smoking, presumably because this was not collected systematically. While the analysis is based on within-person data, the baseline risk is derived from a period (more than 56 days pre-prescription) when smokers are likely still to be smoking, and a period (more than 56 days post-prescription) when a proportion of the smokers may have quit. This makes the results difficult to interpret. |

| **Form No.** | **23** |
| --- | --- |
| Topic | CVD - Epidemiology |
| Author(s) | T J Meine et al. (2005) |
| Title | “Safety and effectiveness of transdermal nicotine patch in smokers admitted with acute coronary syndromes” |
| Source | American Journal of Cardiology, 2005, 95, 976-978 |
| Study type | Prospective study |
| Study location | Duke University, Durham, North Carolina, USA |
| Population studied and inclusion criteria | All smokers who underwent cardiac catheterization after admission for unstable angina pectoris or non-ST-segment AMI from January 2000 to September 2003. |
| Nicotine exposures | Patients prescribed transdermal nicotine patches during their hospital stay were compared with those who were not. |
| Treatment groups and sizes | Unadjusted analyses compared 194 patients prescribed patches with 9797 who were not. Additional analyses compared 187 patients prescribed patches with 187 who were not, matched using propensity scores on various potential confounding variables. |
| Relevant endpoints | 7-day, 30-day and 1-year mortality, coronary bypass, coronary angioplasty |
| Confounding variables | Propensity matched analyses compared groups which did not differ significantly on sex, race, hypertension, hyperlipidemia, previous diabetes mellitus, previous heart failure, previous AMI, ejection fraction (mean ± SD) (%), and AMI. |
| Other relevant study details | The “study had 80% power to detect a 2% mortality increase of 7 days, and a 4% mortality increase at 1 year, with α = 0.05”. |
| Relevant findings |  |
| \|  \|  \| Unadjusted rates (cases) \| \| \| \| \|  \| Matched analysis rates (cases) \| \| \| \| \| \| --- \| --- \| --- \| --- \| --- \| --- \| --- \| --- \| --- \| --- \| --- \| --- \| --- \| \| Endpoint \|  \| No patch (n=9797) \| \| Patch (n=194) \| \| p \|  \| No patch (n=187) \| \| Patch (n=187) \| \| p \| \|  \|  \|  \| \|  \| \|  \|  \|  \| \|  \| \|  \| \| 7-day mortality \|  \| 1.2% \| (120) \| 0.5% \| (1) \| 0.37 \|  \| 0.5% \| (1) \| 0% \| (0) \| 0.3 \| \| 30-day mortality \|  \| 2.4% \| (230) \| 1.6% \| (3) \| 0.5 \|  \| 1.6% \| (3) \| 1.1% \| (2) \| 0.7 \| \| 1-year mortality \|  \| 6.9% \| (673) \| 5.7% \| (11) \| 0.5 \|  \| 5.4% \| (10) \| 4.8% \| (9) \| 0.8 \| \| Coronary bypass \|  \| 15.3% \| (1,500) \| 19.6% \| (38) \| 0.1 \|  \| 13.9% \| (26) \| 19.8% \| (37) \| 0.1 \| \| Coronary angioplasty \|  \| 37.2% \| (3,644) \| 50% \| (97) \| 0.003 \|  \| 42.3% \| (79) \| 50.3% \| (94) \| 0.1 \| | |
| Authors’ main relevant conclusions | “Transdermal nicotine therapy appears safe and does not have an effect on the mortality of patients with acute coronary syndromes.”  “Our finding that nicotine patch therapy did not increase mortality in inpatients admitted with acute coronary syndromes suggests the likelihood of safety in this population and highlights the need for a randomized trial to confirm these findings and explore the possibility of potential benefit.” |
| Strengths and weaknesses mentioned | None |
| Study quality score | FAIR |
| Comments | It is not clear why the authors chose not to use standard methods using data from all the patients to make adjusted comparisons of those prescribed or not prescribed patches. |

| **Form No.** | **24** |
| --- | --- |
| Topic | CVD – Epidemiology |
| Author(s) | L Elzi et al. (2006) |
| Title | “A smoking cessation programme in HIV-infected individuals: a pilot study” |
| Source | Antiviral Therapy, 2006, 11, 787-795 |
| Study type | Prospective |
| Study location | Basel, Switzerland |
| Population studied and inclusion criteria | HIV-infected individuals aged 16 years or older who between April 2000 and March 2002 were assessed for current smoking status and had at least two visits to the clinic, of which a sample were placed on a smoking cessation programme (SCP). |
| Nicotine exposures | Nicotine substitution (patch, tablets, inhalers, gum, spray) offered to participants in program based on individual needs. |
| Treatment groups and sizes | 34 smokers in SCP, 383 control smokers not in SCP, and 263 nonsmokers. |
| Relevant endpoints | CVD-related morbidity and lung cancer during follow-up at about 1 year. |
| Confounding variables | No adjustments made despite many significant differences noted at baseline between smokers in SCP and control smokers, including a history of CHD (15% in SCP, 0.8% in control smokers, p < 0.01). |
| Other relevant study details | None |
| Relevant findings | An AMI during follow-up was seen in 2/34 (5.9%) in the SCP group, in 4/383 (1.0%) in the control smokers and in 5/263 (1.9%) in the nonsmokers. The unadjusted RRs (95% CI) for the SCP group are 5.63 (1.07-29.64) vs the control smokers, and 3.09 (0.62-15.33) vs nonsmokers. These estimated RRs, which are not statistically significant on an exact test, are unadjusted for any factor including past history of CHD. Also noted were percutaneous transluminal coronary angioplasty evident in two individuals in the SCP group and three in the control group, giving an unadjusted RR of 7.51 (1.30-42.41). One member of the control group died of CVD. In the SCP group one patient died of lung cancer, while in the control group one patient died of lung cancer and three developed it during follow-up. |
| Authors’ main relevant conclusions | “A smoking cessation programme is feasible and should be encouraged. The long-term impact of smoking cessation on CVD morbidity and mortality should be evaluated in comparative trials.” |
| Strengths and weaknesses mentioned | Limitations include small number of participants, non-randomized enrolment in intervention programme, participants in program representing a selection of patients with a high cardiovascular risk. |
| Study quality score | POOR |
| Comments | No proper statistical analysis of morbidity or mortality; Users and non-users of NRT are not separated. |

| **Form No.** | **25** |
| --- | --- |
| Topic | CVD - Epidemiology |
| Author(s) | C A Paciullo et al. (2009) |
| Title | “Impact of nicotine replacement therapy on postoperative mortality following coronary artery bypass graft surgery” |
| Source | The Annals of Pharmacotherapy, 2009, 43, 1197-1202 |
| Study type | Prospective study |
| Study location | A 468-bed tertiary medical centre with a 22-bed cardiothoracic/vascular intensive care unit (ICU), presumably (from authors’ addresses) in Lexington, Kentucky, USA |
| Population studied and inclusion criteria | Patients were screened for inclusion who had undergone coronary artery bypass graft (CABG) surgery between August 2004 and August 2007 and received at least one nicotine patch (7, 14 or 21 mg) postoperatively while in the ICU. Patients were excluded if they had any other surgical procedure at the same time as the CABG, were 18 years or less, had an incomplete medical record, or received any other form of NRT. In phase I these patients were compared with non-NRT controls on pack-years smoked and APACHE II (Acute Physiology and Chronic Health Evaluation II) score. In phase II they were compared with unmatched non-NRT controls and with nonsmokers. |
| Nicotine exposures | Patch users are compared with non-patch users |
| Treatment groups and sizes | Phase I : 67 prescribed NRT, 67 current smokers not prescribed NRT  Phase II : 90 prescribed NRT, 489 current smokers not prescribed NRT, 1478 nonsmokers |
| Relevant endpoints | Mortality |
| Confounding variables | In Phase I patients in the two groups were stated to have no significant difference on any of the baseline characteristics studied (age, smoking, APACHE II score, hypertension, hyperlipidemia, heart failure, chronic renal failure, atrial fibrillation, diabetes, cardiopulmonary bypass and COPD). Phase II comparisons were controlled for age and atrial fibrillation status. |
| Other relevant study details | None |
| Relevant findings | In Phase I, non-significant increases in mortality were noted, with 3 deaths (4.5%) in the NRT group, compared with 0 in the non-NRT group (p = 0.08). One of the deaths was due to aspiration pneumonia, two from cardiac arrest.  In Phase II the main relevant results (based on 3 deaths for NRT, 1 for non-NRT, and 2 for nonsmokers) were as follows.   \|  \| Unadjusted OR  (95% CI) \|  \| Adjusted OR  (95% CI) \| \| --- \| --- \| --- \| --- \| \|  \|  \|  \|  \| \| NRT vs non-NRT \| 2.47 (0.74-8.32) \|  \| 6.06 (1.65-22.21) \| \| NRT vs nonsmokers \| 2.28 (0.67-7.77) \|  \| 6.17 (1.62-23.59) \| \| Non-NRT vs nonsmokers \| 0.68 (0.25-1.81) \|  \| 1.00 (0.37-2.71) \|   For the NRT vs non-NRT comparison, adjusted ORs were similar after on-pump CABG [5.22 (0.57-47.65)] and after off-pump CABG [6.49 (1.29-32.56)] though only the latter was statistically significant. |
| Authors’ main relevant conclusions | “The use of NRT in a postoperative CABG surgery population resulted in a significant increase in mortality when adjusted for baseline characteristics. Patients receiving NRT after off-pump cardiac surgery may be particularly susceptible. Additional evaluation in large patients cohorts with prospective controls is warranted.” |
| Strengths and weaknesses mentioned | Low power, incomplete control and confounding variables, and no data on quitting or adherence to NRT. |
| Study quality score | POOR |
| Comments | It was not stated what the period of follow-up was or how this information was obtained.  The conclusions re off-pump cardiac surgery are not statistically justified, as the OR estimates do not vary significantly between off- and on-pump.  The very small number of deaths severely limits the ability to draw inferences. |

| **Form No.** | **26** |
| --- | --- |
| Topic | CVD - Epidemiology |
| Author(s) | K J Woolf et al. (2012) |
| Title | “Effect of nicotine replacement therapy on cardiovascular outcomes after acute coronary syndromes” |
| Source | American Journal of Cardiology, 2012, 110, 968-970 |
| Study type | Prospective study |
| Study location | University of Rochester Medical Centre, New York, USA |
| Population studied and inclusion criteria | Current tobacco users who presented with an acute coronary syndrome (ACS) (including angina pectoris and MI) and underwent cardiac catheterization from January 2006 to June 2010. Patients who died during the index hospitalization were excluded, as were current NRT users at admission. |
| Nicotine exposures | Patients were subdivided according to whether they were prescribed NRT. |
| Treatment groups and sizes | 184 patients prescribed NRT and 479 controls not prescribed NRT. |
| Relevant endpoints | These “included all-cause death, MI, repeat revascularization, or rehospitalization for angina, arrhythmia, or congestive heart failure at 1 year. The prespecified primary end point was a composite of these outcomes, and the individual components were analysed separately as secondary end points”. |
| Confounding variables | Adjustment was made for dialysis status, gender, prior CABG and race. |
| Other relevant study details | A sample size of 918 patients was estimated “to ensure 90% power to detect a statistically significant benefit with NRT use”. |
| Relevant findings | \| Outcome \| NRT \| \| Control \| \| OR (95% C I) \| \| --- \| --- \| --- \| --- \| --- \| --- \| \|  \|  \| \|  \| \|  \| \| Composite^a^ \| 53 \| (29%) \| 149 \| (31%) \| 0.89 (0.61-1.30) \| \| Death \| 7 \| (4%) \| 24 \| (5%) \| 0.80 (0.33-1.91) \| \| AMI \| 8 \| (4%) \| 23 \| (5%) \| 0.90 (0.40-2.06) \| \| Repeat revascularization \| 18 \| (10%) \| 58 \| (12%) \| 0.77 (0.44-1.36) \| \| Hospitalization^b^ \| 41 \| (22%) \| 104 \| (22%) \| 1.01 (0.66-1.53) \| \| ^a^ Death, AMI, repeat revascularization, or hospitalization for angina, congestive heart failure, or arrhythmia.  ^b^ For angina, congestive heart failure, or arrhythmia. \| \| \| \| \| \| |
| Authors’ main relevant conclusions | “NRT use was not associated with an increased risk of adverse cardiovascular events in the first year after ACS.” |
| Strengths and weaknesses mentioned | Unmeasured confounding; assessment of endpoints at the 1-year point, so an early risk of NRT balanced by a later benefit cannot be ruled out; compliance with NRT not verified; tobacco use not accurately assessed; number of patients (663) less than planned (918). |
| Study quality score | FAIR |
| Comments | The study was designed to show a benefit of NRT, but did not do so. Given the size of the ORs observed, significance would not have been demonstrated with the composite endpoint even had the planned sample size been achieved. |

| **Form No.** | **27** |
| --- | --- |
| Topic | CVD – Clinical trial |
| Author(s) | S Rennard et al. (1994) |
| Title | “Nicotine replacement therapy for patients with coronary artery disease” |
| Source | Archives of Internal Medicine, 1994, 154, 989-995 |
| Study type | Double-blind placebo-controlled multicentre RCT |
| Study location | Four medical centres in the USA |
| Population studied and inclusion criteria | Smokers with stable coronary heart disease who wished to stop smoking recruited from general medical and cardiology clinics and through advertising (in unstated time period). Patients had to be aged 21 to 70 years, smoke at least one pack of cigarettes daily, and have a Fagerström score of 7+. Coronary artery disease was documented by at least one of: (1) coronary angiography showing a 60% or greater obstruction of at least one major coronary artery or one of its primary branches; (2) documented AMI; (3) a clinical history typical of angina pectoris together with an exercise treadmill test result or nuclear scan consistent with myocardial ischemia; or (4) prior coronary artery bypass surgery or coronary angioplasty.  Patients were excluded for the following reasons: desire to become pregnant, AMI within 3 months of study entry, unstable angina, vasospastic conditions, symptomatic valvular heart disease, uncontrolled congestive heart failure, serious ventricular arrhythmias, second-degree or higher atrioventricular block, insulin-dependent diabetes mellitus, active peptic ulcer disease, or any condition that would preclude use of transdermal systems or participation in an experimental study. |
| Nicotine exposures | Patients were randomized to receive nicotine patches (14 mg/day) or an identical appearing placebo which delivered <1 mg/day nicotine. After 1 week, patients who had smoked more than 7 cigarettes had their blinded dose increased to 21 mg/day nicotine, or corresponding placebo. They then received their chosen medication for a further four weeks. 40 placebo vs 19 active patients elected upward titration of nicotine dose. |
| Treatment groups and sizes | 156 patients, with 77 allocated to active patches and 79 to placebo patches. |
| Relevant endpoints | Subjects were monitored for the five weeks of the study for various endpoints including all episodes of angina, palpitations, and other cardiac symptoms, as well as adverse events and ECG changes from pre-randomization. |
| Confounding variables | No adjustment for confounding variables. The two groups were found not to differ significantly on sex, age, baseline CO level, various aspects of smoking, Fagerström score, cardiovascular history, and New York Heart Association class (undefined). |
| Other relevant study details | The sample size was derived from a power calculation based on expected differences in smoking cessation rates. |
| Relevant findings | \|  \| Subjects with event \| \|  \| \| --- \| --- \| --- \| --- \| \| Endpoint \| Nicotine  patch  (N=77) \| Placebo patch  (N=79) \| RR  (95% CI)^a^ \| \|  \|  \|  \|  \| \| Premature termination due to adverse events^b^ \| 3 \| 8 \| 0.38 (0.11-1.40) \| \| Hospitalization^c^ \| 0 \| 2 \| 0.21 (0.01-4.20) \| \| Angina at week 1 \| 8 \| 13 \| 0.63 (0.28-1.44) \| \| Angina at week 5^d^ \| 5 \| 7 \| 0.73 (0.24-2.21) \| \| Clinically important ECG changes^e^ \| 22 \| 26 \| 0.87 (0.54-1.39) \| \|  \|  \|  \|  \|   ^a^ Estimated.  ^b^ Of various types, mainly CV related, none significantly increased in the active group.  ^c^ One for chest pain resulting in bypass surgery, one for non-CVD responses attributed to nicotine withdrawal.  ^d^ Data at weeks 2, 3 and 4 also showed no difference.  ^e^ Of various types, none significantly increased in the active group. |
| Authors’ main relevant conclusions | “In summary, although longer-duration studies are indicated, these preliminary results support the safety and efficacy of transdermal nicotine in patients with stable coronary artery disease.” |
| Strengths and weaknesses mentioned | 1. Relatively small number of individuals who underwent testing  2. The brief duration of the study  3. Confounding by possible adverse events due to the stress of quitting |
| Risk of bias | LOW |
| Comments | None |

| **Form No.** | **28A** |
| --- | --- |
| Topic | CVD – Clinical trial |
| Author(s) | A M Joseph et al. (1996) |
| Title | “The safety of transdermal nicotine as an aid to smoking cessation in patients with cardiac disease” |
| Source | The New England Journal of Medicine, 1996, 335, 1792-1798 |
| Study type | Double-blind placebo-controlled RCT |
| Study location | 10 Veterans Affairs medical centres in the USA |
| Population studied and inclusion criteria | Outpatients enrolled between November 28 1994 and June 30 1995 who were smokers, aged 45+, smoked 15+ cigs/day and for 5+ years, had made 2+ quit attempts, and had an expired air CO of 8 ppm. Subjects had to have one or more of the following: history of MI, history of CABG surgery or angioplasty, stenosis of 50+% in at least one coronary artery, or a clinical history of angina, congestive heart failure, cor pulmonale, arrhythmia, peripheral vascular disease or cerebrovascular disease. Exclusion criteria were any of the following in the two weeks pre-randomization: unstable angina, MI, CABG surgery, angioplasty, or hospitalization for cardiac arrhythmia. Also a history of patch use for >48 hours, current use (and unwillingness to stop using) other tobacco products or nicotine gum, an unstable psychiatric illness or disorder involving alcohol or drugs, severe dementia, and pregnancy. |
| Nicotine exposures | Subjects were randomized to nicotine patches (21 mg for 6 weeks, 14 mg for 2 weeks, 7 mg for 2 weeks) or placebo patches of identical size, appearance and odour. |
| Treatment groups and sizes | 584 outpatients (576 men), with 294 allocated to patches and 290 to placebo. |
| Relevant endpoints | Subjects were monitored for 14 weeks for the primary endpoints: - death, MI, cardiac arrest, admission to hospital for increased angina severity, arrhythmia, or congestive heart failure, and outpatient visit for increased severity of atherosclerotic CVD. |
| Confounding variables | No adjustment for confounding variables. The two groups were found not to differ significantly on age, the inclusion criteria, baseline severity of cardiac disease, weight, blood pressure, varying aspects of smoking, Fagerström score, and expired CO. Exceptionally a greater frequency of stenosis was seen in the placebo group (p < 0.01). |
| Other relevant study details | The study was planned to have a 90% power to detect an increase of 10% in the rate of adverse events due to treatment, with an alpha of 0.05 in a one-sided analysis. |
| Relevant findings | \|  \| Subjects with event \| \|  \| \| --- \| --- \| --- \| --- \| \| Endpoint \| Nicotine  patch  (N=294) \| Placebo  (N=290) \| RR  (95% CI)^a^ \| \|  \|  \|  \|  \| \| Death \| 1 \| 6 \| 0.16 (0.02-1.36) \| \| MI or cardiac arrest \| 1 \| 2 \| 0.49 (0.04-5.41) \| \| Admission for increased severity of angina \| 7 \| 10 \| 0.69 (0.27-1.79) \| \| Admission for arrhythmia \| 5 \| 3 \| 1.64 (0.40-6.82) \| \| Admission for congestive heart failure \| 2 \| 2 \| 0.99 (0.14-6.96) \| \| Outpatient visit for increased severity of atherosclerotic CVD \| 12 \| 7 \| 1.69 (0.68-4.23) \| \| Total \| 28 \| 30 \| 0.92 (0.56-1.50) \|   ^a^ Estimated  Similarly non-significant results were reported based on total numbers of events, rather than numbers of subjects with events, and when subjects were separated by smoking status at the time of the event. |
| Authors’ main relevant conclusions | “Transdermal nicotine does not cause a significant increase in cardiovascular events in high-risk outpatients with cardiac disease” |
| Strengths and weaknesses mentioned | 1. Almost complete restriction to male veterans  2. Not possible to verify smoking status at the time of occurrence of the adverse event  3. Limited sample size |
| Risk of bias | LOW |
| Comments | None |

| **Form No.** | **29** |
| --- | --- |
| Topic | CVD – Clinical trial |
| Author(s) | D Tzivoni et al. (1998) |
| Title | “Cardiovascular safety of transdermal nicotine patches in patients with coronary artery disease who try to quit smoking” |
| Source | Cardiovascular Drugs and Therapy, 1998, 12, 239-244 |
| Study type | Double-blind placebo-controlled RCT |
| Study location | Jerusalem, Israel |
| Population studied and inclusion criteria | Coronary patients who wished to stop smoking were recruited from a hospital. Patients had to smoke 15+ cigarettes for 5+ years and have a Fagerström score of 5+. Inclusion criteria included presence of CAD, based on either angiographic evidence of significant coronary disease (>70% narrowing in at least one major coronary artery); stable angina pectoris with a positive exercise test, or a documented previous MI. Exclusion criteria included hypersensitivity to any adhesive cutaneous application; MI, coronary bypass surgery, coronary angioplasty, or stroke within the 3 months prior to screening; >12 ischemic episodes during the 48-hour AEM; diastolic blood pressure >110 mmHg; systolic blood pressure >200 mmHg; reduced left ventricular function; and clinical signs of congestive heart failure (New York Heart Association, class 3 or 4), complex ventricular arrhythmias, or episodes of supraventricular tachycardia of .60 seconds duration. |
| Nicotine exposures | Patients who smoked 20+ cigs/day received patches delivering either 21 mg nicotine (active) or 3 mg (placebo). Patients smoking <20 cigs/day received 14 or 2 mg patches. |
| Treatment groups and sizes | 106 patients, with 52 allocated to active patches and 54 to placebo patches. |
| Relevant endpoints | Subjects were monitored for the two weeks of the study for various endpoints including serious adverse experiences and ischaemic episodes. |
| Confounding variables | No adjustment for confounding variables. There were no notable differences between groups on sex, age, various aspects of smoking and Fagerström score |
| Other relevant study details | Eligible patients attended a smoking cessation programme pre-randomization. Repeated ambulatory ECG monitoring and exercise testing was also carried out. |
| Relevant findings | \|  \| Subjects with event \| \|  \| \| --- \| --- \| --- \| --- \| \| Endpoint \| Nicotine  patch  (N=52) \| Placebo patch  (N=54) \| RR  (95% CI)^a^ \| \|  \|  \|  \|  \| \| Serious adverse experiences^b^ \| 2 \| 1 \| 2.08 (0.19-22.22) \| \| Bypass surgery \| 0 \| 1 \| 0.35 (0.01-8.31) \| \| Ischaemic episode^c^ \| 17 \| 25 \| 0.71 (0.44-1.15) \| \|  \|  \|  \|  \|   ^a^ Estimated.  ^b^ In the nicotine patch group, one patient complained of angina at rest and one developed unstable angina with documented ischaemia. In the placebo patch group, one patient complained of worsening of angina leading to bypass surgery.  ^c^ No significant treatment difference was seen in relation to timing or total number of episodes. |
| Authors’ main relevant conclusions | “The use of nicotine patches did not cause aggravation of myocardial ischemia or arrhythmia in coronary patients and therefore can be used as a method to promote smoking cessation in this high-risk group.” |
| Strengths and weaknesses mentioned | None |
| Risk of bias | LOW |
| Comments | Very short follow-up. No power calculations. |
| **Form No.** | **30A** |
| Topic | CVD – Clinical Trial |
| Author(s) | S M Mohiuddin et al. (2007) |
| Title | “Intensive smoking cessation intervention reduces mortality in high-risk smokers with cardiovascular disease” |
| Source | Chest, 2007, 131, 446-452 |
| Study type | RCT |
| Study location | Creighton University Cardiac Centre, Omaha, Nebraska, USA |
| Population studied and inclusion criteria | Patients aged 30 to 75 years admitted from January 2001 to December 2002 with a diagnosis of ACS or decompensated heart failure who had smoked for 5+ years and had a Fagerström score of >7. Exclusion criteria were failure to speak, read English and current alcohol or illicit substance addiction. |
| Nicotine exposures | Patients were randomized to intensive intervention for smoking cessation or usual care. Intensive intervention included providing, at no cost, individualized adjuvant pharmacotherapy including NRT and/or bupriopion. |
| Treatment groups and sizes | 109 smokers received intensive intervention and 100 usual care. |
| Relevant endpoints | Mortality and hospitalizations over the following two year period. |
| Confounding variables | The two groups were compared on age, sex, ethnicity, education, hospital admission diagnosis, medical history, hospital discharge, medications and smoking. Significant differences noted were more whites in the usual care group (p = 0.03) and more cigs/day in the usual care group (p = 0.03). |
| Other relevant study details | Mortality and hospitalization was compared using the Kaplan-Meier method and log-rank tests respectively. |
| Relevant findings | \|  \| Number of cases (%) \| \|  \|  \| \| --- \| --- \| --- \| --- \| --- \| \|  \| Intervention \| Usual care \|  \|  \| \| Endpoint \| (N=109) \| (N=100) \| RR (95% CI) \| P \| \|  \|  \|  \|  \|  \| \| All cause mortality \| 3 (2.8%) \| 12 (12%) \| 0.23 (0.07-0.73) \| 0.026 \| \| Cardiovascular mortality \| 3 (2.8%) \| 9 (9%) \| 0.31 (0.09-1.10)^a^ \| - \| \| Admissions \| 25 (23%) \| 41 (41%) \| 0.56 (0.37-0.84) \| 0.01 \| \| - CVD \| 20 (18.3%) \| 37 (37%) \| - \| - \| \| - MI \| 9 (8.3%) \| 17 (17%) \| 0.49 (0.23-1.04)^a^ \| - \| \| - Unstable angina \| 8 (7.3%) \| 14 (14%) \| - \| - \| \| - Cardiac arrhythmia \| 1 (0.9%) \| 2 (2%) \| - \| - \| \| - Decompensated heart failure \| 2 (1.8%) \| 4 (4%) \| - \| - \| \| ^a^  Estimated \|  \|  \|  \|  \| |
| Authors’ main relevant conclusions | “The results of our study demonstrate that an intensive smoking cessation intervention in high-risk smokers with cardiovascular disease is not only effective in achieving smoking cessation, but also reduces hospitalizations and total mortality.” |
| Strengths and weaknesses mentioned | “The major limitation of our trial is its relatively small sample size, which limited our ability to perform multivariate analyses to adjust for the impact of other factors on the study outcome. In addition, the provision of adjuvant pharmacotherapy at no cost has not been previously evaluated. It is unlikely that this practice, outside of research environments, can be readily adopted. Whether we could have achieved the same outcomes if smokers had to purchase their smoking cessation medications is unknown.” |
| Risk of bias | HIGH |
| Comments | The study does not specifically relate to NRT, and the results are not given separately for those in the intervention group receiving NRT. There are no power calculations. Survival adjusted RRs or p values were not given by the authors, but the difference for total CVD admissions is almost certainly significant at p < 0.05. |

| **Form No.** | **22B** |
| --- | --- |
| Topic | Stroke - Epidemiology |
| Author(s) | R Hubbard et al. (2005) |
| Title | “Use of nicotine replacement therapy and the risk of acute myocardial infarction, stroke, and death” |
| Source | Tobacco Control, 2005, 14, 416-421 |
| Study type | Case series analysis based on a national database |
| Study location | UK |
| Population studied and inclusion criteria | Patients with at least one prescription for NRT between June 1985 and November 2003. |
| Nicotine exposures | 33247 individuals were prescribed NRT. Comparisons are made of disease incidence in the 56 days before prescription and in the 56 days after. Analyses are presented for all NRT users and separately for patch users, gum/lozenge/microtablet users and inhalers/nasal spray users. |
| Treatment groups and sizes | Of the 33247 studied, 25596 (77%) were prescribed patch, 5421 (16%) gum, lozenge or microtablets, 1614 (5%) inhalers, and 616 (2%) nasal spray. Of the patch users, 18065 (71%) were prescribed a high dose (≥15mg) patch, 6025 (24%) a medium dose (10-14 mg) patch, and 1506 (6%) a low dose (<10 mg) patch. Of the gum users 995 (27%) were given 4 mg gum and 2648 (73%) 2 mg gum. |
| Relevant endpoints | Stroke |
| Confounding variables | Age at time of stroke. |
| Other relevant study details | Power calculations were based on AMI, not stroke (see CAF 22A). Incidence for the four 14 day periods before prescription and the four after prescription were compared relative to that in the remaining, baseline, time periods, using conditional Poisson regression. |
| Relevant findings | 506 NRT users had at least one diagnosis of stroke, of whom 103 had a second and 30 a third. Of the 506 stroke diagnoses, 452 occurred outside the period for analysis, with 243 being more than 56 days before NRT, 8 on the day of the prescription and 201 more than 56 days after NRT. Relative incidence compared to baseline levels was 3.59 (2.56-5.03) in the 56 days before NRT, based on 39 cases, and 1.30 (0.77-2.19) in the 56 days after, based on 15 cases. “The incidence of stroke was … increased in the period leading up to the first NRT prescription.” “In the 56 days after starting treatment there was no significant increase in risk …, although there was an isolated increase in risk during the final quarter of this period.” There was no evidence of effect modification by age or sex, but the incidence ratio before NRT was higher in those with no previous history of angina. There was no evidence of an increased risk after prescription for any particular NRT formulation. Results were similar for second stroke. |
| Authors’ main relevant conclusions | “The use of NRT is not associated with any increase in the risk of myocardial infarction, stroke, or death.”  The increased incidence of MI before the first NRT prescription suggests “that NRT is currently being prescribed shortly after myocardial infarctions and strokes.” “Theoretical concerns over the safety of NRT in relation to these adverse outcomes can therefore probably be discounted, particularly when the alternative for most smokers is to continue to smoke.” |
| Strengths and weaknesses mentioned | “The main strengths of our study are its large size, the long duration of patient follow-up, and the detailed information available on prescriptions. The potential weaknesses of the study are the validity of the outcome data, the extent to which people prescribed NRT actually used the treatment, and the use of NRT purchased without a prescription over the counter.”  We “have excluded the effects of the major likely confounders by using the self-controlled case series method.” |
| Study quality score | FAIR |
| Comments | The study reports no data on smoking, presumably because this was not collected systematically. While the analysis is based on within-person data, the baseline risk is derived from a period (more than 56 days pre-prescription) when smokers are likely still to be smoking, and a period (more than 56 days post-prescription) when a proportion of the smokers may have quit. This makes the results difficult to interpret. |

| **Form No.** | **28B** |
| --- | --- |
| Topic | Stroke – Clinical trial |
| Author(s) | A M Joseph et al. (1996) |
| Title | “The safety of transdermal nicotine as an aid to smoking cessation in patients with cardiac disease” |
| Source | The New England Journal of Medicine, 1996, 335, 1792-1798 |
| Study type | Double-blind placebo-controlled RCT |
| Study location | 10 Veterans Affairs medical centres in the USA |
| Population studied and inclusion criteria | Outpatients enrolled between November 28 1994 and June 30 1995 who were smokers, aged 45+, smoked 15+ cigs/day and for 5+ years, had made 2+ quit attempts, and had an expired air CO of 8 ppm. Subjects had to have one or more of the following: history of MI, history of CABG surgery or angioplasty, stenosis of 50+% in at least one coronary artery, or a clinical history of angina, congestive heart failure, cor pulmonale, arrhythmia, peripheral vascular disease or cerebrovascular disease. Exclusion criteria were any of the following in the two weeks pre-randomization: unstable angina, MI, CABG surgery, angioplasty, or hospitalization for cardiac arrhythmia. Also a history of patch use for >48 hours, current use (and unwillingness to stop using) other tobacco products or nicotine gum, an unstable psychiatric illness or disorder involving alcohol or drugs, severe dementia, and pregnancy. |
| Nicotine exposures | Subjects were randomized to nicotine patches (21 mg for 6 weeks, 14 mg for 2 weeks, 7 mg for 2 weeks) or placebo patches of identical size, appearance and odour. |
| Treatment groups and sizes | 584 outpatients (576 men), with 294 allocated to patches and 290 to placebo. |
| Relevant endpoints | Subjects monitored for 14 weeks for various secondary endpoints including admission to hospital for cerebrovascular disease. |
| Confounding variables | No adjustment for confounding variables. The two groups were found not to differ significantly on age, the inclusion criteria, baseline severity of cardiac disease, weight, blood pressure, varying aspects of smoking, Fagerström score, and expired CO. Exceptionally a greater frequency of stenosis was seen in the placebo group (p < 0.01). |
| Other relevant study details | The study was planned to have a 90% power to detect an increase of 10% in the rate of adverse events due to treatment, with an alpha of 0.05 in a one-sided analysis. |
| Relevant findings | \|  \| Subjects with event \| \|  \| \| --- \| --- \| --- \| --- \| \| Endpoint \| Nicotine  patch  (N=294) \| Placebo  (N=290) \| RR  (95% CI)^a^ \| \|  \|  \|  \|  \| \| Admission for cerebrovascular disease \| 4 \| 3 \| 1.32 (0.30-5.82) \| \| ^a^ Estimated \| \| \| \|   Similarly non-significant results were reported based on total numbers of events, rather than numbers of subjects with events, and when subjects were separated by smoking status at the time of the event. |
| Authors’ main relevant conclusions | “There were no significant differences between groups in the occurrence of secondary endpoints.” |
| Strengths and weaknesses mentioned | 1. Almost complete restriction to male veterans  2. Not possible to verify smoking status at the time of occurrence of the adverse event  3. Limited sample size |
| Risk of bias | LOW |
| Comments | None |

| **Form No.** | **31** |
| --- | --- |
| Topic | Stroke-Epidemiology |
| Author(s) | N G Panos et al. (2010) |
| Title | “Outcomes associated with transdermal nicotine replacement therapy in a neurosurgery intensive care unit” |
| Source | American Journal of Health-System Pharmacy, 2010, 67, 1357-1361 |
| Study type | Prospective study based on review of records |
| Study location | Chicago, USA |
| Population studied and inclusion criteria | Patients older than 18 years admitted to the University of Illinois Medical Center neurosurgery intensive care unit for neurological insults. Data were obtained from pharmacy records, admission logs and computerized patient charts. Patients excluded if they had incomplete data in their medical record, died within three days of admission, or received the first dose of transdermal NRT more than seven days after admission. |
| Nicotine exposures | Transdermal NRT |
| Treatment groups and sizes | 114 smokers received transdermal NRT, 113 smokers did not receive transdermal NRT, and 113 nonsmokers. |
| Relevant endpoints | The primary endpoint was hospital discharge disposition, classified as unfavourable (discharge to rehabilitation facility, nursing home or hospice, or death) or favourable (discharge to home). Secondary endpoints included mortality; lengths of hospital and neurosurgery ICU stays; and rates of subarachnoid haemorrhage rebleeding, ischemic stroke, any intracerebral haemorrhage not related to the admitting diagnosis, and angiographically documented vasospasm after the primary clinical event. |
| Confounding variables | In general, no adjustment for confounding variables, although the NRT group had higher admission rates of subarachnoid haemorrhage, had smoked more cigarettes and for a longer duration, and had longer stays in the intensive care unit and hospital. |
| Other relevant study details | No power calculations |
| Relevant findings | \|  \| Subjects with event \| \|  \| \| --- \| --- \| --- \| --- \| \|  \| NRT \| No NRT \| RR \| \|  \| (N=114) \| (N=113) \| (95% CI)^a^ \| \|  \|  \|  \|  \| \| Unfavourable discharge \| 48 \| 37 \| 1.29 (0.91-1.81) \| \| Death^b^ \| 5 \| 6 \| 0.83 (0.26-2.63) \| \| Subarachnoid haemorrhage rebleeding \| 0 \| 1 \| 0.33 (0.01-8.03) \| \| Any intracerebral haemorrhage during treatment \| 5 \| 1 \| 4.96 (0.59-41.76) \| \| Angiographic vasospasm \| 23 \| 12 \| 1.90 (0.99-3.63) \| \| Ischemic stroke \| 3 \| 6 \| 0.50 (0.13-1.93) \|   a Unadjusted, estimated from data provided  b Cause not stated, but most likely related to the neurologic insult.  Note that the authors only presented numbers, %s and p values for the overall comparison between three groups including also 118 nonsmokers. For angiographically documented vasospasm, an unadjusted p value of 0.016 was noted, based also on 9 cases in nonsmokers, but this became non-significant after adjustment for the presence of subarachnoid haemorrhage at baseline, noted to be more common in smokers who received NRT (56 cases) than in other smokers (32 cases). Doubtless such adjustment would have reduced the unadjusted RR shown above. |
| Authors’ main relevant conclusions | There was no significant difference in unfavourable discharge disposition among neurosurgery ICU patients who were smokers treated with NRT, smokers not treated with NRT, and nonsmokers not treated with NRT. There were no differences in other secondary outcomes among the groups. |
| Strengths and weaknesses mentioned | The definition of discharge disposition may differ from that used by other institutions. The unbalanced baseline characteristics between the three groups |
| Study quality score | FAIR |
| Comments | Only adjusted for potential confounding variables where unadjusted analysis showed a significant difference. |

| **Form No.** | **32** |
| --- | --- |
| Topic | Other serious adverse health effects in patients – Epidemiology |
| Author(s) | R A Carandang et al. (2011) |
| Title | “Nicotine replacement therapy after subarachnoid haemorrhage is not associated with increased vasospasm” |
| Source | Stroke , 2011, 42, 3080-3086 |
| Study type | Prospective study |
| Study location | Massachusetts, USA |
| Population studied and inclusion criteria | All patients with subarachnoid haemorrhage hospitalized from January 1994 to December 2008. Inclusion criteria were “(1) age >18 years; (2) survival through the first 72 hours of hospitalization; (3) documented aneurysm as the cause of SAH; (4) aneurysm repair by either endovascular coiling or surgical clipping within 72 hours of ictus; and (5) active smoking at the time of admission to the hospital.” |
| Nicotine exposures | Patients treated with transdermal nicotine patches were compared with those who were not. Doses of patches ranged from 7 to 21mg and were continued until discharge. |
| Treatment groups and sizes | 87 patients treated with patches were compared with 131 control patients matched on age, sex, Fisher score, aneurysm size and number, hypertension, current medications, and epoch of treatment (±5 years). |
| Relevant endpoints | Length of stay, angiographic and clinical vasospasm, mortality, and “good outcome” - defined as a Glasgow outcome score >4. |
| Confounding variables | Analyses of clinical vasospasm adjusted for Fisher grades. Analyses of good outcome adjusted for severity of clinical disease at baseline (Glasgow Outcome Scale and World Federation of Neurological Surgery scores). The NRT and control subjects were similar at baseline in age, sex, hypertension, aneurysm size and number, and Fisher grades. The NRT group had less severe clinical diseases, particularly as measured by the Hunt-Hess score (p < 0.0001). |
| Other relevant study details | None. |
| Relevant findings | \| Outcome \| Patch  (n=87) \| No Patch  (n=171) \| Unadjusted  p \| Adjusted OR (95% CI) \| \| --- \| --- \| --- \| --- \| --- \| \|  \|  \|  \|  \|  \| \| Total length of stay \| 17.4 (SD 9.5) \| 21.5 (SD 12.4) \| 0.0168 \| - \| \| Death \| 2.3% (2) \| 7.0% (12) \| 0.0709^a^ \| 0.36 (0.07-1.43)^a,b^ \| \| Angiographic vasospasm \| 44.8% (39) \| 52.6% (90) \| 0.24 \| 0.85 (0.65-1.12)^a,b^ \| \| Clinical vasospasm \| 19.5% (17) \| 32.8% (56) \| 0.026 \| 0.45 (0.23-0.88) \| \| Good outcome^c^ \| 81.6% (71) \| 62.6% (107) \| 0.0052 \| 2.17 (1.19-3.97) \|   ^a^ Estimated  ^b^ Unadjusted RR  ^c^ Glasgow Outcome Score >4 |
| Authors’ main relevant conclusions | “Nicotine replacement therapy was not associated with increased angiographic vasospasm and was associated with less clinical vasospasm and better Glasgow Outcome Score scores on discharge.” |
| Strengths and weaknesses mentioned | Weaknesses included no quantification of cigarette consumption, or of medical complications that may have influenced outcomes as well as the dosage of patches, limited follow-up not allowing better functional outcome methods. Incomplete follow-up neuroimaging, and selection bias. |
| Study quality score | FAIR |
| Comments | While NRT patches were not randomly allocated, and prescription was clearly related to disease severity, the results certainly do not indicate any adverse effects of the patches. |

| **Form No.** | **28C** |
| --- | --- |
| Topic | Other serious adverse health effects – Clinical trial in patients |
| Author(s) | A M Joseph et al. (1996) |
| Title | “The safety of transdermal nicotine as an aid to smoking cessation in patients with cardiac disease” |
| Source | The New England Journal of Medicine, 1996, 335, 1792-1798 |
| Study type | Double-blind placebo-controlled RCT |
| Study location | 10 Veterans Affairs medical centres in the USA |
| Population studied and inclusion criteria | Outpatients enrolled between November 28 1994 and June 30 1995 who were smokers, aged 45+, smoked 15+ cigs/day and for 5+ years, had made 2+ quit attempts, and had an expired air CO of 8 ppm. Subjects had to have one or more of the following: history of MI, history of CABG surgery or angioplasty, stenosis of 50+% in at least one coronary artery, or a clinical history of angina, congestive heart failure, cor pulmonale, arrhythmia, peripheral vascular disease or cerebrovascular disease. Exclusion criteria were any of the following in the two weeks pre-randomization: unstable angina, MI, CABG surgery, angioplasty, or hospitalization for cardiac arrhythmia. Also a history of patch use for >48 hours, current use (and unwillingness to stop using) other tobacco products or nicotine gum, an unstable psychiatric illness or disorder involving alcohol or drugs, severe dementia, and pregnancy. |
| Nicotine exposures | Subjects were randomized to nicotine patches (21 mg for 6 weeks, 14 mg for 2 weeks, 7 mg for 2 weeks) or placebo patches of identical size, appearance and odour. |
| Treatment groups and sizes | 584 outpatients (576 men), with 294 allocated to patches and 290 to placebo. |
| Relevant endpoints | Subjects monitored for 14 weeks for various secondary endpoints including admission to hospital for peripheral vascular disease, and for other reasons not related to CVD or stroke. |
| Confounding variables | No adjustment for confounding variables. The two groups were found not to differ significantly on age, the inclusion criteria, baseline severity of cardiac disease, weight, blood pressure, varying aspects of smoking, Fagerström score, and expired CO. Exceptionally a greater frequency of stenosis was seen in the placebo group (p < 0.01). |
| Other relevant study details | The study was planned to have a 90% power to detect an increase of 10% in the rate of adverse events due to treatment, with an alpha of 0.05 in a one-sided analysis. |
| Relevant findings | \|  \| Subjects with event \| \|  \| \| --- \| --- \| --- \| --- \| \| Endpoint \| Nicotine  patch  (N=294) \| Placebo  (N=290) \| RR  (95% CI)^a^ \| \|  \|  \|  \|  \| \| Admission for peripheral vascular disease \| 3 \| 5 \| 0.59 (0.14-2.45) \| \| Admission for other reasons^b^ \| 16 \| 13 \| 1.21 (0.59-2.48) \| \| Total \| 19 \| 18 \| 1.04 (0.56-1.94) \| \| ^a^ Estimated.  ^b^ “Other reasons” includes admissions for chronic obstructive pulmonary disease, upper gastrointestinal bleeding, hemoptysis, pneumonia, deep venous thrombosis, hyperglycemia, otitis, pulmonary nodule, hernia repair, renal stone, pulmonary embolus, or colon cancer. \| \| \| \|   Similarly non-significant results were reported based on total numbers of events, rather than numbers of subjects with events, and when subjects were separated by smoking status at the time of the event. |
| Authors’ main relevant conclusions | “There was no significant difference between groups in the occurrence of secondary endpoints.” |
| Strengths and weaknesses mentioned | 1. Almost complete restriction to male veterans  2. Not possible to verify smoking status at the time of occurrence of the adverse event  3. Limited sample size |
| Risk of bias | LOW |
| Comments | None |

| **Form No.** | **30B** |
| --- | --- |
| Topic | Other serious adverse health effects – Clinical trial in patients |
| Author(s) | S M Mohiuddin et al. (2007) |
| Title | “Intensive smoking cessation intervention reduces mortality in high-risk smokers with cardiovascular disease” |
| Source | Chest, 2007, 131, 446-452 |
| Study type | RCT |
| Study location | Creighton University Cardiac Centre, Omaha, Nebraska, USA |
| Population studied and inclusion criteria | Patients aged 30 to 75 years admitted from January 2001 to December 2002 with a diagnosis of ACS or decompensated heart failure who had smoked for 5+ years and had a Fagerström score of >7. Exclusion criteria were failure to speak, read English and current alcohol or illicit substance addiction. |
| Nicotine exposures | Patients were randomized to intensive intervention for smoking cessation or usual care. Intensive intervention included providing, at no cost, individualized adjuvant pharmacotherapy including NRT and/or bupriopion. |
| Treatment groups and sizes | 109 smokers received intensive intervention and 100 usual care. |
| Relevant endpoints | Mortality and hospitalization from non-cardiac causes over the following two year period |
| Confounding variables | The two groups were compared on age, sex, ethnicity, education, hospital admission diagnosis, medical history, hospital discharge, medications and smoking. Significant differences noted were more whites in the usual care group (p = 0.03) and more cigs/day in the usual care group (p = 0.03). |
| Other relevant study details | None. |
| Relevant findings | \|  \| Number of cases (%) \| \| \| --- \| --- \| --- \| \|  \| Intervention \| Usual care \| \| Endpoint \| (N-109) \| (N=100) \| \|  \|  \|  \| \| Non-CVD mortality \| 0 (0.0%) \| 3 (3%) \| \| - respiratory failure \| 0 \| 2 \| \| - cancer \| 0 \| 1 \| \|  \|  \|  \| \| Non-cardiac hospitalizations \| 5 (4.6%) \| 4 (4%) \| \| - diabetic foot infection \| 0 \| 1 \| \| - exacerbation of COPD \| 3 \| 2 \| \| - cancer treatment \| 1 \| 1 \| \| - pancreatitis \| 1 \| 0 \|   No differences were statistically significant |
| Authors’ main relevant conclusions | “The results of our study demonstrate that an intensive smoking cessation intervention in high-risk smokers with cardiovascular disease is not only effective in achieving smoking cessation, but also reduces hospitalizations and total mortality.” |
| Strengths and weaknesses mentioned | “The major limitation of our trial is its relatively small sample size, which limited our ability to perform multivariate analyses to adjust for the impact of other factors on the study outcome. In addition, the provision of adjuvant pharmacotherapy at no cost has not been previously evaluated. It is unlikely that this practice, outside of research environments, can be readily adopted. Whether we could have achieved the same outcomes if smokers had to purchase their smoking cessation medications is unknown.” |
| Risk of bias | HIGH |
| Comments | The study does not specifically relate to NRT, and the results are not given separately for those in the intervention group receiving NRT. There are no power calculations. |

| **Form No.** | **33** |
| --- | --- |
| Topic | Other serious adverse health effects – Clinical trial in patients |
| Author(s) | S M Lee et al. (2013) |
| Title | “The effectiveness of a perioperative smoking cessation program: a randomized clinical trial” |
| Source | Anesthesia & Analgesia, 2013, 117, 605-613 |
| Study type | RCT |
| Study location | London, Ontario, Canada |
| Population studied and inclusion criteria | Subjects scheduled for surgery who were enrolled between October 2010 and May 2012 who were current daily smokers (>2 cigs/day, smoked in last 7 days), aged >18 years, and presented to the preadmission clinic at least 3 weeks preoperatively. Exclusion criteria included pregnancy, breastfeeding, poor proficiency in the English language, active participation in another smoking cessation trial, and inability to consent due to severe mental illness or dementia. In addition, patients with allergy to nicotine or Nicoderm®, and those with unstable angina or unstable arrhythmia. |
| Nicotine exposures | Subjects were randomly allocated to usual care, which involved no specific smoking cessation intervention, or to an intervention which included smoking cessation and a free 6-week supply of transdermal NRT. For smokers of 10 cigarettes per day or more, a 4-week supply of 21 mg/d, 1-week supply of 14 mg/d, and 1-week supply of 7 mg/d patches were provided. Smokers of <10 cigarettes per day were supplied with 4 weeks of 14 mg/d patches and 2 weeks of 7 mg/d patches. |
| Treatment groups and sizes | 84 patients were allocated to the usual care group and 84 to the intervention group. Due to missing data, analyses are based on slightly reduced numbers. |
| Relevant endpoints | Intraoperative and immediate postoperative complications occurring in the post-anaesthetic care unit (PACU); unanticipated hospital admissions; length of hospital say; duration of care in the PACU; complications in the 30-day post operative period; and unscheduled visits in the 30-day post operative period. |
| Confounding variables | No adjustment for confounding variables. The two groups were noted to be balanced as regards age, sex, BMI, types of surgery, and smoking variables, but the interaction group was noted to have slightly more cases of diabetes, hypertension and heart disease. |
| Other relevant study details | Power calculations were based on anticipated smoking cessation rates |
| Relevant findings | \| Outcome \| Intervention \| Usual care \| RR (95% CI) \| p \| \| --- \| --- \| --- \| --- \| --- \| \|  \|  \|  \|  \|  \| \| Complications \|  \|  \|  \|  \| \| - Intraoperative \| 6.3% (5/80) \| 7.5% (6/80) \| 0.83 (0.27-2.6) \| 1.0 \| \| - Post operative (immediate) \| 2.5% (2/80) \| 6.4% (5/78) \| 0.39 (0.08-2.0) \| 0.27 \| \| - Any time^a^ \| 13.1% (11/84) \| 18.7% (14/84) \| 0.79 (0.38-1.63) \| 0.67 \| \| - in 30 day follow-up period \| 16.2% (12/74) \| 11.6% (8/69) \| 1.40 (0.61-3.2) \| 0.48 \| \|  \|  \|  \|  \|  \| \| Unanticipated hospital admission \| 2.5% (2/80) \| 2.6% (2/78) \| 0.98 (0.14-6.8) \| 1.0 \| \| Unscheduled visit during postoperative period \| 21.6% (16/74) \| 13.0% (9/69) \| 1.66 (0.78-3.6) \| 0.19 \| \|  \|  \|  \| Difference (95% CI) \|  \| \|  \|  \|  \|  \|  \| \| Hospital length of stay \| 1.75 (1.1 to 3.1) \| 2.1 (1.4 to 3.2) \| −0.35(−1.4 to 0.9) \| 0.36 \|   ^a^ It is not made clear what period this related to.  It is also noted that “the time until PACU discharge readiness was significantly lower in the intervention group, as illustrated in the Kaplan-Meier plot.” |
| Authors’ main relevant conclusions | “The smoking cessation intervention did not contribute to any increase in cardiopulmonary complications.” |
| Strengths and weaknesses mentioned | “This study did not significantly investigate overall postoperative complications.” “This study was underpowered to see differences in complications between groups.” “The external validity of this clinical trial may be limited by the large number of patients who refused to participate in the study.” |
| Risk of bias | HIGH |
| Comments | The study is not specifically of NRT, but of an intervention which includes NRT. |

| **Form No.** | **34** |
| --- | --- |
| Topic | Other serious adverse health effects – Clinical trial in patients |
| Author(s) | T Thomsen et al. (2010) |
| Title | “Brief smoking cessation intervention in relation to breast cancer surgery: a randomized trial” |
| Source | Nicotine & Tobacco Research, 2010, 12, 1118-1124 |
| Study type | RCT |
| Study location | Three hospitals in Denmark |
| Population studied and inclusion criteria | Patients scheduled for breast cancer surgery, who were women, daily smokers, aged 18+ years, and have sufficient language proficiency. Exclusion criteria included weekly alcohol >35 units, substance abuse, diagnosed psychiatric disease, dementia, American Society of Anesthesiologists’ (ASA) physical status classification-class>3, penetrating cancer, pregnancy and lactation, and preoperative neoadjuvant chemotherapy. |
| Nicotine exposures | Subjects were randomly allocated to a control group, which involved inconsistent or no advice about smoking, or to an intervention group, which included smoking cessation advice and a free supply of NRT, given in accordance with patient preferences and dependency. |
| Treatment groups and sizes | 62 patients were allocated to the control group, and 58 to the intervention group. |
| Relevant endpoints | Any wound complication, any complication, secondary surgery. (There were no deaths or readmissions.) |
| Confounding variables | No adjustment for confounding variables. “Patients were comparable in the intervention and control groups with regard to baseline characteristics except for a longer duration of surgery in intervention group patients.” |
| Other relevant study details | The surgery was planned to include 130 patients scheduled for breast cancer surgery. This was based on a power calculation with 80% power at the 5% significance level and an anticipated 66% reduction in wound complications, including seroma requiring aspiration (from 30% to 10%). |
| Relevant findings | \|  \| Intervention \| Control \|  \|  \| \| --- \| --- \| --- \| --- \| --- \| \| Outcome \| (n = 57) \| (n = 62) \| RR (95% CI) \| p \| \|  \|  \|  \|  \|  \| \| Any wound complication \| 44% (25) \| 45% (28) \| 0.97 (0.65-1.45) \| 1.00 \| \| Any complication^a^ \| 61% (38) \| 61% (35) \| 1.00 (0.75-1.33) \| 1.00 \| \| Secondary surgery \| 2% (1) \| 0% (0) \|  \|  \|   ^a^ Also includes respiratory complications (17 cases vs 21), cardiovascular complications (2 vs 1), nausea and vomiting (4 vs 1), and other complications (2 vs 1). |
| Authors’ main relevant conclusions | “Brief smoking intervention administered shortly before breast cancer surgery modestly increased self-reported perioperative smoking cessation without having any clinical impact on postoperative complications.” |
| Strengths and weaknesses mentioned | Strengths included secure allocation assessment, blinded outcome assessment and rigorous follow-up. Weaknesses included small sample size, high refusal rate to enter the study (50%) and patients revealing their group allocation to the blinded assessors. |
| Risk of bias | HIGH |
| Comments | The study is not specifically of NRT, but of an intervention which includes NRT. |

| **Form No.** | **35** |
| --- | --- |
| Topic | Other serious adverse health effects – meta-analysis of smoking cessation trials in healthy individuals |
| Author(s) | S Greenland et al. (1998) |
| Title | “A meta-analysis to assess the incidence of adverse effects associated with the transdermal nicotine patch” |
| Source | Drug Safety, 1998, 18, 297-308 |
| Meta-analysis type | Meta-analysis based on data from 47 reports of 35 RCTs. |
| Location of studies | No restriction; no details of locations given. |
| Populations studied and inclusion criteria | RCTs with at least 20 subjects in each treatment arm, with adverse effect data presented. |
| Searches | Articles for the analysis were identified by Ciba Geigy and by a MEDLINE search (up to December 1, 1996) based on the terms transdermal, nicotine and human. |
| Nicotine exposures | RCTs of nicotine patch use, two trials also involved nicotine and placebo patch recipients. One trial involved only different doses of nicotine patches. Most patch groups used patches containing 17 to 25 mg nicotine, but 4 used ≥28 mg, 10 used 14 or 15 mg, and 2 used 7 or 8 mg. 9 “placebo” patches contained small doses of nicotine.  Dosage of nicotine for a study was estimated as the average initial dose assigned, though it varied by bodyweight or smoking status in some studies. |
| Numbers of subjects considered | 41 groups of nicotine patch recipients totalling 5501 subjects, and 33 groups of placebo recipients totalling 3752 subjects. |
| Relevant endpoints | The main endpoint of most studies was smoking cessation, but two studies concerned ulcerative colitis. Results for a wide variety of health outcomes are presented; the relevant ones are shown under “relevant findings” below. |
| Relevant findings:   \| Endpoint \| Nicotine patch  events/patients \| Other treatment  events/patients \| RR (95% CI)^a^ \| RR per 21 mg  nicotine (95% CI)^b^ \| \| --- \| --- \| --- \| --- \| --- \| \|  \|  \|  \|  \|  \| \| AMI \| 3/36^c^ \| 3/362 \| 1.00 (0.17-5.83) \| - \| \| Stroke \| 1/354 \| 2/357 \| 0.54 (0.02-6.73) \| - \| \| Tachycardia \| 2/239 \| 0/238 \| - \| - \| \| Palpitations \| 2/446 \| 8/451 \| 0.26 (0.04-1.10) \| - \| \| Angina \| 1/239 \| 1/238 \| 1.00 (0.025-39) \| - \| \| Arrhythmia \| 11/406 \| 9/411 \| 1.26 (0.56-2.87) \| 1.43 (0.48-4.24) \| \| Hypertension \| 8/354 \| 5/357 \| 1.60 (0.52-5.48) \| 1.79 (0.50-6.45) \| \| Asthma \| 0/115 \| 2/119 \| - \| - \| \| Bronchitis \| 9/115 \| 5/119 \| 1.91 (0.63-6.54) \| 2.12 (0.62-7.27) \| \| Urogenital symptoms \| 0/115 \| 1/119 \| - \| - \| \| Neurological symptoms \| 4/115 \| 1/159 \| 3.80 (0.51-10.6) \| - \| \| ^a^ RR not estimated if zero cases in one group  ^b^ RR not estimated if <5 cases in total  ^c^ 36 is as given, but probably a typographical error for a 3 digit number starting with 36 \| \| \| \| \| | |
| Authors’ main relevant conclusions | “Despite the large number of patients in the analysis, few adverse cardiovascular outcomes (myocardial infarction, stroke, tachycardia, arrhythmia, angina) were reported, and no excess of these outcomes was detected among patients assigned to nicotine-patch use.” |
| Study quality assessment | No assessment was made of the quality of individual studies. |
| Strengths and weaknesses mentioned | “The present data also leave open other important questions. It appears that the acute effects of the nicotine patch on serious outcomes (such as myocardial infarction and stroke) cannot be determined reliably from the randomised placebo-controlled trials performed to date, because the risk of these outcomes was simply too low in the studies to have yielded enough events for an informative analysis; this is so despite the fact that the trials involved over 9000 participants and is no doubt in part because of the cardiovascular exclusions used in most of the trials. Our analysis also could not address the effect of nicotine patch use in adolescents.” |
| Comments | While the small number of events limits precision of the estimates, the meta-analysis does demonstrate that there is clearly no common serious health effect associated with nicotine patches. |

| **Form No.** | **36** |
| --- | --- |
| Topic | Other serious adverse health effects – meta-analysis of smoking cessation trials in healthy individuals |
| Author(s) | D Moore et al. (2009) |
| Title | “Effectiveness and safety of nicotine replacement therapy assisted reduction to stop smoking: systematic review and meta-analysis” |
| Source | British Medical Journal, 2009, 338, 867-871 |
| Meta-analysis type | Systematic review and meta-analysis based on 7 placebo-controlled RCTs |
| Location of studies | No restriction; 5 studies were conducted in Europe, 1 in the USA and 1 in Australia. |
| Populations studied and inclusion criteria | “Eligible studies were published or unpublished randomised controlled trials that enrolled smokers who declared no intention to quit smoking in the short term, and compared nicotine replacement therapy (with or without motivational support) with placebo, no treatment, other pharmacological therapy, or motivational support, and reported quit rates.” |
| Searches | Data sources were “Cochrane Library, Medline, Embase, CINAHL, PsychINFO, Science Citation Index, registries of ongoing trials, reference lists, the drug company that sponsored most of the trials, and clinical experts.” |
| Nicotine exposures | 2 studies used nicotine gum (4 mg), 2 nicotine gum (2 or 4 mg),2 an inhaler (10 mg), and 1 a choice of gum (4 mg), inhaler (10 mg) or patch (25 mg). The period of intervention varied between 6 and 18 months. |
| Numbers of subjects considered | 1384 subjects allocated to NRT, 1383 to placebo |
| Relevant endpoints | The main endpoint was abstinence from smoking in all studies. Results reported for three relevant endpoints: death, serious adverse event, discontinued because of adverse event |
| Relevant findings | \| Endpoint \| NRT  Events/patients \| Placebo  Events/patients \| RR (95% CI) \| \| --- \| --- \| --- \| --- \| \|  \|  \|  \|  \| \| Death \| 4/1384 \| 4/1383 \| 1.00 (0.25-4.02) \| \| Serious adverse event^a^ \| 86/1119 \| 79/1114 \| 1.09 (0.79-1.50) \| \| Discontinued because of adverse event \| 19/1119 \| 15/1114 \| 1.27 (0.64-2.51) \|   ^a^ None judged likely to have been due to treatment; no individual study reported a significant increase |
| Authors’ main relevant conclusions | “There was no evidence of an increase in life threatening problems, and nicotine replacement therapy was well tolerated, with almost no difference in discontinuation because of side effects in those receiving nicotine replacement therapy compared with those receiving placebo.” |
| Study quality assessment | All seven studies classified as “high quality” by the authors |
| Strengths and weaknesses mentioned | None mentioned |
| Comments | The systematic nature of the review, which appears to have been well conducted, is a strength. The small number of serious adverse health effects is a limitation, though it does demonstrate the lack of common serious health effects associated with NRT. |

| **Form No.** | **37** |
| --- | --- |
| Topic | Other serious adverse health effects – meta-analysis of smoking cessation trials in healthy individuals |
| Author(s) | E J Mills et al. (2010) |
| Title | “Adverse events associated with nicotine replacement therapy (NRT) for smoking cessation. A systematic review and meta-analysis of one hundred and twenty studies involving 177,390 individuals.” |
| Source | Tobacco Induced Diseases, 2010, 8, 1-15. |
| Meta-analysis type | Systematic review and meta-analysis of 92 RCTs and 28 observational studies |
| Location of studies | No restriction; The RCT were most often conducted in the USA (40), Sweden (13), England (11), Denmark (4), Australia (3) and Switzerland (3). 19 observational studies were conducted in the USA, with no more than 2 in any other country. |
| Populations studied and inclusion criteria | RCTs had a “duration beyond 4 weeks” and compared “NRT with an inert control (e.g. placebo or standard of care)”. 74 were in healthy adults, 6 in populations with medical or psychiatric co-morbidities, 4 in pregnant women, 3 in hospitalized patients, 3 in adolescents, 1 in postmenopausal women, and 1 in surgical patients.  Observational studies had to examine the proportion of adverse events occurring. 16 observational studies were in healthy adults, 7 in mixed healthy and unhealthy adult populations, 2 in populations with medical co-morbidities, 2 in adolescents and 1 in an older population. |
| Searches | Searches used “10 databases (from inception to November 20, 2009): MEDLINE, EMBASE, Cochrane CENTRAL, AMED, CINAHL, TOXNET, Development and Reproductive Toxicology, Hazardous Substances Databank, Psych-info and Web of Science”, and the terms “nicotine” AND “smoking” AND gum” OR “Patch” OR “spray” OR “inhalers” OR “Tablet” OR “lozenge” AND “clinical trial.” |
| Nicotine exposures | Any form of NRT delivery was considered. 42 RCTs evaluated patch, 26 gum, 6 nasal spray, 6 inhaler, 4 tablet, 1 lozenge, and 35 combinations. Duration varied from 1 to 24 months, with varying levels of dosage.  17 observational studies evaluated patch, 1 gum, 2 nasal spray, and 8 combinations. Duration varied from 4 to 26 months, with varying levels of dosage. |
| Numbers of subjects considered | The 92 RCTs involved 32,185 subjects and the 28 observational studies involved 145,205 subjects. |
| Relevant endpoints | Analyses were conducted of heart palpitations and chest pains, mortality, and serious adverse events. These were characterized as “life-threatening events occurring during the trial period” and included “all-cause mortality, myocardial infarction, all-cause strokes, incidence of all-cancers, all-hospitalizations, suicidal ideation, depression, and incidence of diabetes.” |
| Relevant findings | RCTs. Heart palpitations and chest pains reported in 12 RCTs with incidence higher (p < 0.001) for NRT (189/6249 = 3.0%) than controls (64/2985 = 1.6%), with the OR 2.06 (95% CI 1.51-2.82) and no heterogeneity. No increase for mortality (11/1387 = 0.8%) vs 16/1378 = 1.2%, OR 0.74 (0.33-1.67), based on 8 studies. The authors noted that “25 RCTs reported serious adverse events occurring, but none were statistically significant (data not shown).”  Observational studies. Prevalences were noted in NRT groups, but not compared with non-treated groups. The prevalence of heart palpitations and chest pain was 3.6% (95% CI 1.4%-9.6%) in the seven studies reporting this. Serious adverse events were single cases of transient visual field impairment, right-hemisphere stroke, MI and an urticarial reaction from a patch. Finally, in one large study of self-harm (n = 63,265), 141 cases of fatal and nonfatal self-harm cases were identified (0.2%), as well as 30 cases of suicidal ideation. |
| Authors’ main relevant conclusions | “The use of NRT is associated with a variety of side effects. In addition to counseling and medical monitoring, clinicians should inform patients of potential side effects which are associated with the use of NRT for the treatment of tobacco dependence.” |
| Study quality assessment | “Study quality evaluation included general methodological reporting features including allocation concealment, sequence generation, blinding status, intention-to-treat, and appropriate descriptions of loss to follow-up. In rating quality, failure to report a quality component of study design (e.g. blinding) was treated the same as not employing it.” |
| Strengths and weaknesses mentioned | “Our review has several limitations. These include limitations of the primary studies themselves as well as those associated with combining results across potentially heterogeneous studies or populations. The main limitation of the primary studies is the mechanism by which adverse events are recorded. In the majority of instances this would be through passive reporting and therefore be susceptible to the underreporting associated with such techniques.” |
| Comments | This is the most comprehensive information source on serious health effects in healthy populations, though actually some studies considered were of those with co-morbidities or surgical patients. Results are, unfortunately, not available specifically for healthy populations. |

| **Form No.** | **38** |
| --- | --- |
| Topic | Other serious adverse health effects – meta-analysis of smoking cessation trials in healthy individuals |
| Author(s) | L F Stead et al. (2012) |
| Title | “Nicotine replacement therapy for smoking cessation (Review)” |
| Source | The Cochrane Collaboration (2012), 267pp |
| Meta-analysis type | Systematic review and meta-analysis based on 150 trials |
| Location of studies | No restriction. 77 studies in North America, 60 in Europe, 5 in Australasia, and 8 in other or multiple locations. |
| Populations studied and inclusion criteria | RCTs and trials where allocation to treatment was by a “quasi-randomized” method were also included. Male or female smokers were motivated to quit irrespective of recruitment setting or level of nicotine dependence. Studies randomizing therapists, rather than smokers, to offer NRT or a control, were also included, but not trials randomizing therapists to receive an educational intervention, including encouraging their patients to receive NRT. Included were trials comparing NRT versus placebo or no NRT control, different doses of NRT, different types of NRT, NRT with bupropion and combinations of the two, and NRT use pre- and post-quitting. |
| Searches | The specialized register of the Cochrane Tobacco Addiction Group was searched “in July 2012 for any reports of trials making reference to the use of nicotine replacement therapy of any type, by searching for ’NRT’, or ’nicotine’ near to terms for nicotine replacement products in the title, abstract or keywords.” |
| Nicotine exposures | NRT exposures included chewing gum, transdermal patches, nasal and oral spray, inhalers and tablets or lozenges. |
| Numbers of subjects considered | 117 trials with over 50,000 participants contributed to the primary comparison between any type of NRT and a placebo or non-NRT control. |
| Relevant endpoints | Data were collected on adverse events in both included and excluded studies, where they were reported. No attempt was made to pool these findings, apart from one meta-analysis of reports of palpitation, tachycardia or chest pain. |
| Relevant findings | No systematic attempt was made “in this review to synthesize quantitatively the incidence of the various side effects reported with the different NRT preparations. This was because of the extensive variation in reporting the nature, timing and duration of symptoms.” However, following the meta-analysis by Mills et al. (2010) (see CAF 35) they attempted to replicate their findings by presenting a combined analysis of data for palpitations/chest pains. Based on data from 15 studies, an overall OR of 1.88 (95% CI 1.37-2.57) was estimated, with a heterogeneity p value of 0.35. This was based on 165 cases out of 6673 subjects given NRT and 62 cases out of 4401 subjects given placebo. |
| Authors’ main relevant conclusions | “There is no evidence that NRT increases the risk of heart attacks.” Commenting on the results for chest pains and heart palpitations, the authors stated “This is potentially the only clinically significant serious adverse event to emerge from the trials, and constitutes an extremely rare event, occurring at a rate of 2.5% in the NRT groups compared with 1.4% in the control groups in the 15 trials in which it was reported at all.” |
| Study quality assessment | Studies were individually assessed as being of high, moderate, low or very low quality based on “risks of selection bias (methods of sequence generation, and allocation concealment), performance and detection bias (the presence or absence of blinding), attrition bias (levels and reporting of loss to follow-up), and any other threats to study quality.” |
| Strengths and weaknesses mentioned | The studies were individually classified as being of low, high or unclear risk of bias. “The majority of studies either did not report how randomization was performed and allocation concealed, or reported them in insufficient detail to determine whether a satisfactory attempt to control selection bias had been made (rated as being at unclear risk).” … “The main findings were not sensitive to the exclusion from the meta-analysis of trials at unclear risk, or of trials at unclear and at high risk of bias.” |
| Comments | This extensive review is predominantly concerned with smoking cessation and provides very little information on serious health effects. Where results are given it is not clear what the endpoint is as the methods (on p6) refer to “a meta-analysis of reports of palpitations, tachycardia or chest pains”, the results section (on p18) only to “chest pains and heart palpitations” and the analysis table itself (on p237) to “both” palpitations/chest pains” and to “palpitations” only, while giving only one set of results. |

##### SUPPLEMENTARY FILE 2

##### Study quality and risk of bias

Methods

For epidemiological cohort and cross-sectional studies, the relevant quality assessment tool of the NIH (National Heart Lung and Blood Institute 2014b) was used. This tool defines 14 full criteria, as listed below, with entries as Yes, No, or Other (CD = cannot determine, NA = not applicable, NR = not reported). The shortened forms are used in the tables of results.

| Full criteria | | Short form |
| --- | --- | --- |
|  |  |  |
| 1. | Was the research question or objective in this paper clearly stated? | Research question stated |
| 2. | Was the study population clearly specified and defined? | Population defined |
| 3. | Was the participation rate of eligible persons at least 50%? | Participation rate 50+% |
| 4. | Were all the subjects selected or recruited from the same or similar populations (including the same time period)? Were inclusion and exclusion criteria for being in the study prespecified and applied uniformly to all participants? | Uniformity of subjects |
| 5. | Was a sample size justification, power description, or variance and effect estimates provided? | Sample size justification |
| 6. | For the analyses in this paper, were the exposure(s) of interest measured prior to the outcome(s) being measured? | Exposure before outcome |
| 7. | Was the timeframe sufficient so that one could reasonably expect to see an association between exposure and outcome if it existed? | Timeframe long enough |
| 8. | For exposures that can vary in amount or level, did the study examine different levels of the exposure as related to the outcome (e.g. categories of exposure, or exposure measured as continuous variable)? | Dose-response analyses |
| 9. | Were the exposure measures (independent variables) clearly defined, valid, reliable, and implemented consistently across all study participants? | Exposures defined and valid |
| 10. | Was the exposure(s) assessed more than once over time? | Multiple exposure assessment |
| 11. | Were the outcome measures (dependent variables) clearly defined, valid, reliable, and implemented consistently across all study participants? | Outcome defined and valid |
| 12. | Were the outcome assessors blinded to the exposure status of participants? | Blind outcome assessment |
| 13. | Was the loss to follow-up after baseline 20% or less? | Loss to follow-up <20% |
| 14. | Were key potential confounding variables measured and adjusted statistically for their impact on the relationship between exposure(s) and outcome(s)? | Adequate confounder control |

Based on general guidance described in the tool, an overall assessment is then made as Good, Fair of Poor. Finally, any additional comments can be made. In particular, a reason for an assessment of Poor should be given.

For epidemiological case-control studies, the relevant quality assessment tool of the NIH is again used (National Heart Lung and Blood Institute 2014b). This tool defines 12 full criteria, as listed below, with entries as Yes, No, or Other (CD = cannot determine, NA = not applicable, NR = not reported). The shortened forms are used in the tables of results.

| Full criteria | | Short form |
| --- | --- | --- |
|  |  |  |
| 1. | Was the research question or objective in this paper clearly stated and appropriate? | Research question stated |
| 2. | Was the study population clearly specified and defined? | Population defined |
| 3. | Did the authors include a sample size justification? | Sample size justification |
| 4. | Were controls selected or recruited from the same or similar population that gave rise to the cases (including the same timeframe?) | Uniformity of subjects |
| 5. | Were the definitions, inclusion and exclusion criteria, algorithms or processes used to identify or select cases and controls valid, reliable, and implemented consistently across all study participants? | Consistency of all processes |
| 6. | Were the cases clearly defined and differentiated from controls? | Cases clearly defined |
| 7. | If less than 100 percent of eligible cases and/or controls were selected for the study, were the cases and/or controls randomly selected from those eligible? | Random selection of <100% |
| 8. | Was there use of concurrent controls? | Concurrent controls |
| 9. | Were the investigators able to confirm that the exposure/risk occurred prior to the development of the condition or event that defined a participant as a case? | Exposures before outcome |
| 10. | Were the measures of exposure/risk clearly defined, valid, reliable, and implemented consistently (including the same time period) across all study participants? | Exposures defined and valid |
| 11. | Were the assessors of exposure/risk blinded to the case or control status of participants? | Blind exposure assessment |
| 12. | Were key potential confounding variables measured and adjusted statistically in the analyses? If matching was used, did the investigators account for matching during study analysis? | Adequate confounder control |

Again, an overall assessment is made as Good, Fair or Poor, with any additional comments, in particular for Poor studies.

For clinical trials, risk of bias was assessed using the Cochrane Collaboration’s Tool (Higgins et al. 2011). There are seven sources of bias, as listed below, with risk of bias for each to be defined as low, unclear or high. The shortened forms are used in the tables of results.

| Full criteria | | Short form |
| --- | --- | --- |
|  |  |  |
| 1. | Random sequence generation (selection bias) | Random sequence generation |
| 2. | Allocation concealment (selection bias) | Allocation concealment |
| 3. | Blinding of participants and personnel (performance bias) | Blinding of subjects/personnel |
| 4. | Blinding of outcome assessment (detection bias) | Blinding of subjects outcome |
| 5. | Incomplete outcome data (attrition bias) | Incomplete outcome |
| 6. | Selective reporting (reporting bias) | Selective reporting |
| 7. | Anything else, ideally prespecified (other bias) | Other |

An overall assessment of risk of bias as low, unclear or high is then made, with any overall comments as necessary.

Note that, although the source (Higgins et al. 2011) suggests that for items 3, 4 and 5 assessments should be made for each main outcome or class of outcomes, in practice a risk assessment was made for all relevant outcomes from the study.

Results

The three tables below, 2/1 (cohort and cross-sectional), 2/2 (case-control) and 2/3 (clinical trials) summarize the findings.

##### Study quality – cohort and cross-sectional studies^a^

|  | | CAF Number | | | | | | | |
| --- | --- | --- | --- | --- | --- | --- | --- | --- | --- |
| Criteria | | 1 | 2 | 3 | 4 | 5 | 6 | 7 | 8 |
|  | |  |  |  |  |  |  |  |  |
| 1 | Research question stated | Yes | Yes | Yes | Yes | Yes | Yes | Yes | Yes |
| 2 | Population defined | Yes | Yes | Yes | Yes | Yes | Yes | Yes | Yes |
| 3 | Participation rate 50+% | NA | No^b^ | No | No^b^ | No | No | No | Yes |
| 4 | Uniformity of subjects | Yes | Yes | Yes | Yes | Yes | Yes | Yes | Yes |
| 5 | Sample size justification | NA | No | No | No | No | No | No | No |
| 6 | Exposure before outcome | Yes | Yes | Yes | Yes | Yes | Yes | Yes | Yes |
| 7 | Timeframe long enough | No | Yes | Yes | Yes | Yes | Yes | Yes | Yes |
| 8 | Dose-response analyses | Yes | No | No | No | No | No | No | No |
| 9 | Exposures defined and valid | Yes | Yes | Yes | Yes | Yes | Yes | Yes | Yes |
| 10 | Multiple exposure assessment | Yes | No | No | Yes | Yes | Yes | No | No |
| 11 | Outcome defined and valid | Yes | Yes | Yes | Yes | Yes | Yes | Yes | Yes |
| 12 | Blind outcome assessment | NR | NR | NR | NR | NR | NR | NR | NR |
| 13 | Loss to follow-up <20% | Yes | Yes | Yes | Yes | Yes | Yes | No | Yes |
| 14 | Adequate confounder control | Yes | CD | Yes | Yes | Yes | CD | CD | CD |
| Overall study quality | | GOOD | GOOD | GOOD | GOOD | GOOD | GOOD | GOOD | FAIR |
| Comments: | |  |  |  |  |  |  |  |  |
| Adjustment for smoking post-NRT | | Yes | No | No | Yes | No | No | No | No |

##### Study quality – cohort and cross-sectional studies^a^ (continued)

|  | | CAF No. | | | | | | | |
| --- | --- | --- | --- | --- | --- | --- | --- | --- | --- |
| Criteria | | 9 | 22 | 23 | 24 | 25 | 26 | 31 | 32 |
|  | |  |  |  |  |  |  |  |  |
| 1 | Research question stated | Yes | Yes | Yes | Yes | Yes | Yes | Yes | Yes |
| 2 | Population defined | Yes | Yes | Yes | Yes | Yes | Yes | Yes | Yes |
| 3 | Participation rate 50+% | NA | NA | NA | Yes | NA | NA | NA | NA |
| 4 | Uniformity of subjects | Yes | Yes | Yes | Yes | Yes | Yes | Yes | Yes |
| 5 | Sample size justification | No | Yes | Yes | No | No | Yes | Yes | No |
| 6 | Exposure before outcome | Yes | Yes | Yes | Yes | Yes | Yes | Yes | Yes |
| 7 | Timeframe long enough | Yes | CD | CD | CD | CD | CD | CD | CD |
| 8 | Dose-response analyses | No | No | No | No | No | No | No | No |
| 9 | Exposures defined and valid | No | Yes | Yes | No | Yes | Yes | Yes | Yes |
| 10 | Multiple exposure assessment | Yes | Yes | No | No | No | No | No | No |
| 11 | Outcome defined and valid | Yes | Yes | Yes | Yes | Yes | Yes | Yes | Yes |
| 12 | Blind outcome assessment | No | No | No | No | NR | No | No | No |
| 13 | Loss to follow-up <20% | NR | NA | NR | Yes | NR | NR | No | NR |
| 14 | Adequate confounder control | Yes | No | Yes | No | No | No | No | Yes |
| Overall study quality | | FAIR | FAIR | FAIR | POOR^d^ | POOR^e^ | FAIR | FAIR | FAIR |
| Comments: | |  |  |  |  |  |  |  |  |
| Adjustment for smoking post-NRT | | No | No | No | No | No | No | No | No |
| ^a^ Note that SAFs 2 to 7 all describe results from the Spanish National Birth Cohort  ^b^ Not stated, but inferred from other publications on Danish National Birth Cohort (e.g. CAFs 3, 5)  ^c^ As regards hyperactivity/inattention score  ^d^ Study of only 34 patients enrolled in smoking cessation program, with NRT made available but not necessarily used  ^e^ Very small number of deaths; period of follow-up not stated | | | | | | | | | |

Abbreviations used: CD = cannot determine; NA = not applicable; NR = not reported

##### Study quality – case-control studies

|  | | CAF No. |
| --- | --- | --- |
| Criteria | | 21 |
|  | |  |
| 1 | Research question stated | Yes |
| 2 | Population defined | Yes |
| 3 | Sample size justification | Yes |
| 4 | Uniformity of subjects | Yes |
| 5 | Consistency of all processes | Yes |
| 6 | Cases clearly defined | Yes |
| 7 | Timeframe long enough | Yes |
| 8 | Dose-response analyses | No |
| 9 | Exposures defined and valid | Yes |
| 10 | Multiple exposure assessment | Yes |
| 11 | Blind exposure assessment | NR |
| 12 | Adequate confounder control | Yes |
| Overall study quality | | FAIR |
| Comments: | |  |
| Adjustment for smoking post-NRT | | No |

Abbreviations used: CD = cannot determine; NA = not applicable; NR = not reported

##### Risk of bias – clinical trials

|  | | CAF Number | | | | | | |
| --- | --- | --- | --- | --- | --- | --- | --- | --- |
|  | | 10 | 11 | 12 | 13^a^ | 14 | 16^b^ | 17 |
| Criteria | |  |  |  |  |  |  |  |
| 1 | Random sequence generation | Low | Low | NA | Low | Low | Low | Low |
| 2 | Allocation concealment | Low | Low | NA | Unclear | Low | Low | Low |
| 3 | Blinding of subjects/personnel | Low | Low | NA | Unclear | Low | Low | Low |
| 4 | Blinding of outcome | Low | Low | NA | Unclear | Low | Low | Low |
| 5 | Incomplete outcome | Low | Low | Low | Unclear | Low | Low | Low |
| 6 | Selective reporting | Low | Low | Low | Low | Low | Low | Low |
| 7 | Other | Low | Low | Low | Low | Low | Low | Low |
|  |  |  |  |  |  |  |  |  |
| Overall risk of bias | | Low | Low | High | Unclear | Low | Low | Low |
| Comments: | |  |  |  |  |  |  |  |
| Randomized to intensive intervention, not NRT | | No | No | NA | No | No | No | No |
| Adjustment for post-NRT smoking changes | | No | No | NA | No | No | No | No |

##### Risk of bias – clinical trials (continued)

|  | | CAF Number | | | | | | |
| --- | --- | --- | --- | --- | --- | --- | --- | --- |
|  | | 18 | 27 | 28 | 29 | 30 | 33 | 34 |
| Criteria | |  |  |  |  |  |  |  |
| 1 | Random sequence generation | Low | Low | Low | Low | Low | Low | Low |
| 2 | Allocation concealment | Low | Low | Low | Low | High | High | High |
| 3 | Blinding of subjects/personnel | Low | Low | Low | Low | High | High | High |
| 4 | Blinding of outcome | Low | Low | Low | Low | Low | Low | Low |
| 5 | Incomplete outcome | Low | Low | Low | Low | Low | Low | Low |
| 6 | Selective reporting | Low | Low | Low | Low | Low | Low | Low |
| 7 | Other | Low | Low | Low | Low | High | High | High |
|  |  |  |  |  |  |  |  |  |
| Overall risk of bias | | Low | Low | Low | Low | High | High | High |
| Comments: | |  |  |  |  |  |  |  |
| Randomized to intensive intervention, not NRT | | No | No | No | Yes | Yes | Yes | Yes |
| Adjustment for post-NRT smoking changes | | No | No | No | Yes | No | No | No |

^a^ Note that risk of bias details are not shown for CAF 15 as they relate to the same study as for CAF 13

^b^ Note that risk of bias details are now shown for CAF 19 and 20 as they related to the same study as for CAF 16.

Abbreviations used: CD = cannot determine; NA = not applicable; NR = not reported

##### SUPPLEMENTARY FILE 3

##### Studies on NRT and reproduction/development

| CAF  No. | Reference | | Brief study description |
| --- | --- | --- | --- |
|  |  | |  |
| **Epidemiological studies** | | |  |
|  | |  |  |
| 2 | | Morales-Suarez-Varela et al. (2006) | Prospective study based on Danish National Birth Cohort, involving 76,768 pregnant women of which 250 used NRT and did not smoke. Compared rates of congenital malformations in offspring by maternal smoking and NRT use in pregnancy.  STUDY QUALITY = GOOD |
|  | |  |  |
| 3 | | Strandberg-Larsen et al. (2008) | Prospective study based on Danish National Birth Cohort, involving 87,032 pregnant women of which 3,118 used NRT. Compared rates of stillbirth by maternal smoking and NRT use in pregnancy.  STUDY QUALITY = GOOD |
|  | |  |  |
| 4 | | Lassen et al. (2010) | Prospective study based on Danish National Birth Cohort, involving 68,156 women of which 1,825 used NRT. Related birthweight to maternal use of NRT in pregnancy.  STUDY QUALITY = GOOD |
|  | |  |  |
| 5 | | Torp-Pedersen et al. (2010) | Prospective study based on Danish National Birth Cohort, involving 96,842 children, with an estimated 2,800 women using NRT. Compared rates of strabismus by maternal NRT use in pregnancy.  STUDY QUALITY = GOOD |
|  | |  |  |
| 6 | | Milidou et al. (2012) | Prospective study based on Danish National Birth Cohort, involving 63,128 children, with 1,452 mothers using NRT. Related odds of infantile colic in the first six months to maternal smoking and NRT use in pregnancy.  STUDY QUALITY = GOOD |
|  | |  |  |
| 7 | | Zhu et al. (2014) | Prospective study based on Danish National Birth Cohort, involving 84,803 children, with 814 mothers using NRT and not smoking. Compared rates of attention-deficit/hyperactivity disorder, parent-rated hyperactivity/inattention score and birthweight by maternal smoking and NRT use in pregnancy.  STUDY QUALITY = GOOD |
|  | |  |  |
| 8 | | Gaither et al. (2009) | Cross-sectional study in four US states in 5,716 women assessed postnatally, of which 225 had been recommended or prescribed NRT in pregnancy. Compared rates of low birthweight and preterm birth by smoking and NRT use in pregnancy.  STUDY QUALITY = FAIR |
|  | |  |  |
| 9 | | Dhalwani et al. (2015) | Prospective study using another mother-child primary care records for 192,498 children born in the UK. Compared rates of major congenital abnormalities in 2,677 smokers prescribed NRT, 9,980 smokers not prescribed NRT and 179,841 nonsmokers.  STUDY QUALITY = FAIR |
|  | |  |  |
| **Clinical trials** | | |  |
|  | |  |  |
| 10 | | Wisborg et al. (2000) | Double-blind placebo-controlled RCT in Aarhus, Denmark. Compared birthweight, low birthweight and preterm delivery in 124 pregnancy smokers allocated to nicotine patches and 126 allocated to placebo patches.  RISK OF BIAS = LOW |
|  | |  |  |
| 11 | | Kapur et al. (2001) | Double-blind placebo-controlled RCT in Ontario, Canada. 17 pregnant smokers were allocated to nicotine patches and 13 to placebo patches, but study terminated prematurely due to an adverse event.  RISK OF BIAS = LOW |
|  | |  |  |
| 12 | | Schroeder et al. (2002) | One-sample clinical trial in Minnesota, USA. 21 pregnant smokers initiated nicotine patches, with cases of severe infant morbidity noted.  RISK OF BIAS = HIGH |
|  | |  |  |
| 13 | | Pollak et al. (2007) | Open-label multicentre RCT in North Carolina, USA. A range of endpoints were compared in 59 pregnant smokers allocated to cognitive behavioural therapy only, and 122 allocated to cognitive behavioural therapy in conjunction with NRT.  RISK OF BIAS = UNCLEAR |
|  | |  |  |
| 14 | | Oncken et al. (2008) | Placebo-controlled RCT in Connecticut and Maine, USA. A range of endpoints were compared in 100 pregnant smokers allocated to nicotine gum and 94 allocated to placebo gum.  RISK OF BIAS = LOW |
|  | |  |  |
| 15 | | Swamy et al. (2009) | Same study as considered in CAF 13. The analyses relate to serious adverse events following a review of medical records of 52 of the pregnant smokers allocated to cognitive behavioural therapy only, and 105 allocated to cognitive behavioural therapy in conjunction with NRT. |
|  | |  |  |
| 16 | | Coleman et al. (2012) | Double-blind placebo-controlled multicentre RCT in the Midlands and North-West England. A range of endpoints recorded at or before birth were compared in 521 pregnant smokers allocated to nicotine patches and 529 allocated to placebo patches.  RISK OF BIAS = LOW |
|  | |  |  |
| 17 | | El-Mohandes et al. (2013) | Placebo-controlled RCT in Washington D.C., USA. Gestational age and birthweight were compared in 26 pregnant women allocated to cognitive behavioural therapy only and 26 allocated to cognitive behavioural therapy in conjunction with nicotine patches.  RISK OF BIAS = LOW |
|  | |  |  |
| 18 | | Berlin et al. (2014) | Double-blind placebo-controlled multicentre RCT in France. A range of endpoints were compared in 203 pregnant women allocated nicotine patches and 199 allocated placebo patches.  RISK OF BIAS = LOW |
|  | |  |  |
| 19 | | Cooper et al. (2014a) | Same study as considered in CAFs 16 and 20. Provides fuller details of the study, but no additional relevant results. |
|  | |  |  |
| 20 | | Cooper et al. (2014b) | Same study as considered in CAFs 16 AND 19. Provides results relating to infant and maternal outcomes at 2 years. |

##### SUPPLEMENTARY FILE 4

##### Studies on NRT and CVD

| CAF  No. | Reference | | Brief study description |
| --- | --- | --- | --- |
|  | | |  |
| **Epidemiological studies** | | |  |
|  | | |  |
| 21 | | Kimmel et al. (2001) | Case-control study of smokers in Pennsylvania, USA. 653 cases of first MI, 2990 controls. Primary exposure measure is ever use of patches within one week of MI (cases) or interview (controls).  STUDY QUALITY = FAIR |
|  | |  |  |
| 22A | | Hubbard et al. (2005) | Case series analysis from UK national database. 33247 individuals prescribed NRT. Compared AMI incidence in 56 days pre- and post-prescription.  STUDY QUALITY = FAIR |
|  | |  |  |
| 23 | | Meine et al. (2005) | Prospective study in North Carolina, USA of smokers undergoing cardiac catheterization. Compared mortality over a year and further coronary surgery in 194 patients prescribed patches and 9797 not prescribed patches.  STUDY QUALITY = FAIR |
|  | |  |  |
| 24 | | Elzi et al. (2006) | Prospective study in Switzerland of HIV-infected individuals. Compared morbidity and mortality over about a year in 34 taking part in a smoking cessation program including offering NRT, 383 other smokers and 263 nonsmokers.  STUDY QUALITY = POOR |
|  | |  |  |
| 25 | | Paciullo et al. (2009) | Prospective study in Kentucky, USA of patients undergoing coronary bypass surgery. Compared mortality over an unstated period in 90 patients prescribed patches, 489 current smokers not prescribed patches and 1478 nonsmokers.  STUDY QUALITY = POOR |
|  | |  |  |
| 26 | | Woolf et al. (2012) | Prospective study in New York, USA of patients undergoing cardiac catheterization. Compared mortality over a year and cardiac readmission in 184 patients prescribed any form of NRT and 479 not prescribed NRT.  STUDY QUALITY = FAIR |
|  | | |  |
| **Clinical trials** | | |  |
|  | | |  |
| 27 | | Rennard et al. (1994) | Double-blind placebo-controlled RCT in 4 US medical centres of smokers with stable coronary disease. Compared episodes of cardiac symptoms, adverse events and ECG changes over 5 weeks in 77 patients allocated to nicotine patches and 79 allocated to placebo patches.  RISK OF BIAS = LOW |
|  | |  |  |
| 28A | | Joseph et al. (1996) | Double-blind placebo-controlled RCT in 10 US medical centres of smokers with cardiac disease. Compared mortality, MI, cardiac arrest and readmissions over 14 weeks in 294 patients allocated to nicotine patches and 290 allocated to placebo patches.  RISK OF BIAS = LOW |
|  | |  |  |
| 29 | | Tzivoni et al. (1998) | Double-blind placebo-controlled RCT in Jerusalem, Israel of coronary patients who smoked. Compared serious adverse experiences and ischaemic episodes over a 2 week period in 52 patients allocated to nicotine patches and 54 allocated to placebo patches.  RISK OF BIAS = LOW |
|  | |  |  |
| 30A | | Mohiuddin et al. (2007) | RCT in Nebraska, USA of high-risk smokers with CVD. Compared mortality and hospitalizations over a 2 year period in 109 smokers receiving intensive intervention (sometimes including provision of NRT) and 100 receiving usual care.  RISK OF BIAS = HIGH |

##### SUPPLEMENTARY FILE 5

##### Studies in patients of NRT and other serious adverse health effects

| CAF  No. | Reference | | Brief study description |
| --- | --- | --- | --- |
|  |  | |  |
| **Epidemiological studies** | | |  |
|  | | |  |
| 32 | | Carandang et al. (2011) | Prospective study in Massachusetts, USA of smokers hospitalized with subarachnoid haemorrhage. Compared length of stay, angiographic and clinical vasospasm, mortality and outcome in 87 patients treated with patches and 171 matched untreated patients.  STUDY QUALITY = FAIR |
|  | |  |  |
| **Clinical trials** | | |  |
|  | | |  |
| 28C | | Joseph et al. (1996) | Double-blind placebo-controlled RCT in 10 US medical centres of patients with cardiac disease. Compared admissions to hospital for peripheral vascular disease, cerebrovascular disease, and for other reasons not related to CVD or stroke over 14 weeks in 294 patients allocated to patches and 290 allocated to placebo patches.  RISK OF BIAS = LOW |
|  | |  |  |
| 30B | | Mohiuddin et al. (2007) | RCT in Nebraska, USA of high-risk smokers with CVD. Compared mortality and hospitalizations for non-cardiac causes over a 2 year period in 109 smokers receiving intensive intervention (sometimes including provision of NRT) and 100 receiving usual care.  RISK OF BIAS = HIGH |
|  | |  |  |
| 33 | | Lee et al. (2013) | RCT in Ontario, Canada of smokers scheduled for surgery. Compared incidence of complications, hospital admissions, length of hospital stay and duration of care in 84 patients allocated to an intervention including a free supply of patches and 84 allocated to usual care.  RISK OF BIAS = HIGH |
|  | |  |  |
| 34 | | Thomsen et al. (2010) | RCT in Denmark of smokers scheduled for breast cancer surgery. Compared incidence of complications and secondary surgery in 58 patients allocated to an intervention including a free supply of NRT and 62 allocated to a control group.  RISK OF BIAS = HIGH |

##### SUPPLEMENTARY FILE 6

##### Meta-analyses of NRT and other serious adverse health effects

| CAF  No. | Reference | Brief description of meta-analysis |
| --- | --- | --- |
|  |  |  |
| 35 | Greenland et al. (1998) | RCTs of nicotine patches with at least 20 subjects in each treatment arm, with adverse effect data presented. Most studies were smoking cessation trials; two concerned ulcerative colitis. Results compared 41 nicotine patch groups (5501 subjects), and 33 placebo groups (3752 subjects), some studies not having placebo groups. |
| 36 | Moore et al. (2009) | Placebo-controlled RCTs of various forms of NRT in smokers declaring no intention to quit in the short-term. All studies were of smoking reduction. Results, based on seven studies, compared 1384 subjects allocated to NRT, and 1383 allocated to placebo. |
| 37 | Mills et al. (2010) | RCTs (of duration >4 wks) and observational smoking cessation trials of any form of NRT, with data on adverse events. Of 92 RCTs, 74 were in healthy adults, while of 28 observational studies 16 were. The 92 RCTs involved 32185 subjects and the 28 observational studies involved 145205 subjects. |
| 38 | Stead et al. (2012) | Smoking cessation trials of any form of NRT. 117 trials with over 50,000 participants contributed to the primary comparison between any type of NRT and a placebo or non-NRT control. |

##### References

Berlin I, Grange G, Jacob N, Tanguy M-L (2014) Nicotine patches in pregnant smokers: randomised, placebo controlled, multicentre trial of efficacy. BMJ 348:g1622 doi:10.1136/bmj.g1622.

Carandang RA, Barton B, Rordorf GA, Ogilvy CS, Sims JR (2011) Nicotine replacement therapy after subarachnoid hemorrhage is not associated with increased vasospasm. Stroke 42(11):3080-3086 doi:10.1161/STROKEAHA.111.620955.

Coleman T, Cooper S, Thornton JG, Grainge MJ, Watts K, Britton J, Lewis S (2012) A randomized trial of nicotine-replacement therapy patches in pregnancy. N Engl J Med 366(9):808-818 doi:10.1056/NEJMoa1109582.

Cooper S et al. (2014a) The SNAP trial: a randomised placebo-controlled trial of nicotine replacement therapy in pregnancy--clinical effectiveness and safety until 2 years after delivery, with economic evaluation. Health Technol Assess 18(54):1-128 doi:10.3310/hta18540.

Cooper S, Taggar J, Lewis S, Marlow N, Dickinson A, Whitemore R, Coleman T (2014b) Effect of nicotine patches in pregnancy on infant and maternal outcomes at 2 years: follow-up from the randomised, double-blind, placebo-controlled SNAP trial. Lancet Respir Med 2(9):728-737 doi:10.1016/S2213-2600(14)70157-2.

Dhalwani NN, Szatkowski L, Coleman T, Fiaschi L, Tata LJ (2015) Nicotine replacement therapy in pregnancy and major congenital anomalies in offspring. Pediatrics 135(5):859-867 doi:10.1542/peds.2014-2560.

El-Mohandes AAE, Windsor R, Tan S, Perry DC, Gantz MG, Kiely M (2013) A randomized clinical trial of trans-derman nicotine replacement in pregnant African-American smokers. Matern Child Health J 17(5):897-906.

Elzi L et al. (2006) A smoking cessation programme in HIV-infected individuals: a pilot study. Antivir Ther 11(6):787-795.

Gaither KH, Brunner Huber LR, Thompson ME, Huet-Hudson YM (2009) Does the use of nicotine replacement therapy during pregnancy affect pregnancy outcomes? Matern Child Health J 13(4):497-504 doi:10.1007/s10995-008-0361-1.

Greenland S, Satterfield MH, Lanes SF (1998) A meta-analysis to assess the incidence of adverse effects associated with the transdermal nicotine patch. Drug Saf 18(4):297-308.

Higgins JPT et al. (2011) The Cochrane Collaboration's tool for assessing risk of bias in randomised trials. BMJ 343:889-893.

Hubbard R, Lewis S, Smith C, Godfrey C, Smeeth L, Farrington P, Britton J (2005) Use of nicotine replacement therapy and the risk of acute myocardial infarction, stroke, and death. Tob Control 14:416-421.

Joseph AM et al. (1996) The safety of transdermal nicotine as an aid to smoking cessation in patients with cardiac disease. N Engl J Med 335:1792-1798.

Kapur B, Hackman R, Selby P, Klein J, Koren G (2001) Randomized, double-blind, placebo-controlled trial of nicotine replacement therapy in pregnancy. Curr Ther Res Clin Exp 62:274-278 doi:10.1016/S0011-393X(01)80011-4.

Kimmel SE, Berlin JA, Miles C, Jaskowiak J, Carson JL, Strom BL (2001) Risk of acute first myocardial infarction and use of nicotine patches in a general population. J Am Coll Cardiol 37:1297-1302.

Lassen TH, Madsen M, Skovgaard LT, Strandberg-Larsen K, Olsen J, Andersen AM (2010) Maternal use of nicotine replacement therapy during pregnancy and offspring birthweight: a study within the Danish National Birth Cohort. Paediatr Perinat Epidemiol 24(3):272-281 doi:10.1111/j.1365-3016.2010.01104.x.

Lee SM, Landry J, Jones PM, Burhrmann O, Morley-Forster P (2013) The effectiveness of a perioperative smoking cessation program: a randomized clinical trial. Anesth Analg 117(3):605-613 doi:10.1213/ANE.0b013e318298a6b0.

Meine TJ, Patel MR, Washam JB, Pappas PA, Jollis JG (2005) Safety and effectiveness of transdermal nicotine patch in smokers admitted with acute coronary syndromes. Am J Cardiol 95(8):976-978.

Milidou I, Henriksen TB, Jensen MS, Olsen J, Søndergaard C (2012) Nicotine replacement therapy during pregnancy and infantile colic in the offspring. Pediatrics 129(3):e652-e658 doi:10.1542/peds.2011-2281.

Mills EJ, Wu P, Lockhart I, Wilson K, Ebbert JO (2010) Adverse events associated with nicotine replacement therapy (NRT) for smoking cessation. A systematic review and meta-analysis of one hundred and twenty studies involving 177,390 individuals. Tob Induc Dis 8:8 doi:10.1186/1617-9625-8-8.

Mohiuddin SM, Mooss AN, Hunter CB, Grollmes TL, Cloutier DA, Hilleman DE (2007) Intensive smoking cessation intervention reduces mortality in high-risk smokers with cardiovascular disease. Chest 131(2):446-452.

Moore D, Aveyard P, Connock M, Wang D, Fry-Smith A, Barton P (2009) Effectiveness and safety of nicotine replacement therapy assisted reduction to stop smoking: systematic review and meta-analysis. BMJ 338:867-871.

Morales-Suarez-Varela MM, Bille C, Christensen K, Olsen J (2006) Smoking habits, nicotine use, and congenital malformations. Obstet Gynecol 107(1):51-57.

Murray RP, Connett JE, Zapawa LM (2009) Does nicotine replacement therapy cause cancer? Evidence from the Lung Health Study. Nicotine Tob Res 11(9):1076-1082 doi:10.1093/ntr/ntp104.

National Heart Lung and Blood Institute (2014a, (Accessed Oct 2014)) Quality assessment of case-control studies - Last updated March 2014. National Heart, Lung and Blood Institute, National Institutes of Health, US Department of Health and Human Services, Bethesda. <https://www.nhlbi.nih.gov/health-pro/guidelines/in-develop/cardiovascular-risk-reduction/tools/case-control.htm>.

National Heart Lung and Blood Institute (2014b, (Accessed Oct 2014)) Quality assessment tool for observational cohort and cross-sectional studies - Last updated March 2014. National Heart, Lung and Blood Institute, National Institutes of Health, US Department of Health and Human Services, Bethesda. <http://www.nhlbi.nih.gov/health-pro/guidelines/in-develop/cardiovascular-risk-reduction/tools/cohort.htm>.

Oncken C, Dornelas E, Greene J, Sankey H, Glasmann A, Feinn R, Kranzler HR (2008) Nicotine gum for pregnant smokers: a randomized controlled trial. Obstet Gynecol 112(4):859-867 doi:10.1097/AOG.0b013e318187e1ec.

Paciullo CA, Short MR, Steinke DT, Jennings HR (2009) Impact of nicotine replacement therapy on postoperative mortality following coronary artery bypass graft surgery. Ann Pharmacother 43(7):1197-1202 doi:10.1345/aph.1L423.

Panos NG, Tesoro EP, Kim KS, Mucksavage JJ (2010) Outcomes associated with transdermal nicotine replacement therapy in a neurosurgery intensive care unit. Am J Health Syst Pharm 67(16):1357-1361 doi:10.2146/ajhp090402.

Pollak KI et al. (2007) Nicotine replacement and behavioral therapy for smoking cessation in pregnancy. Am J Prev Med 33(4):297-305.

Rennard S et al. (1994) Nicotine replacement therapy for patients with coronary artery disease. Working Group for the Study of Transdermal Nicotine in Patients with Coronary Artery Disease. Arch Intern Med 154(9):989-995.

Schroeder DR, Ogburn PLJ, Hurt RD, Croghan IT, Ramin KD, Offord KP, Moyer TP (2002) Nicotine patch use in pregnant smokers: smoking abstinence and delivery outcomes. J Matern Fetal Neonatal Med 11:100-107.

Stead LF, Perera R, Bullen C, Mant D, Hartmann-Boyce K, Cahil K, Lancaster T (2012) Nicotine replacement therapy for smoking cessation (review). The Cochrane Collaboration. John Wiley & Sons Limited. <http://onlinelibrary.wiley.com/doi/10.1002/14651858.CD000146.pub4/abstract>.

Strandberg-Larsen K, Tinggaard M, Nybo Andersen AM, Olsen J, Grønbaek M (2008) Use of nicotine replacement therapy during pregnancy and stillbirth: a cohort study. BJOG 115(11):1405-1410 doi:10.1111/j.1471-0528.2008.01867.x.

Swamy GK et al. (2009) Predictors of adverse events among pregnant smokers exposed in a nicotine replacement therapy trial. Am J Obstet Gynecol 201(4):354-357 doi:10.1016/j.ajog.2009.06.006.

Thomsen T, Tønnesen H, Okholm M, Kroman N, Maibom A, Sauerberg M-L, Møller AM (2010) Brief smoking cessation intervention in relating to breast cancer surgery: a randomized controlled trial. Nicotine Tob Res 12(11):1118-1124 doi:10.1093/ntr/ntq158.

Torp-Pedersen T, Boyd HA, Poulsen G, Haargaard B, Wohlfahrt J, Holmes JM, Melbye M (2010) In-utero exposure to smoking, alcohol, coffee, and tea and risk of strabismus. Am J Epidemiol 171(8):868-875.

Tzivoni D, Keren A, Meyler S, Khoury Z, Lerer T, Brunel P (1998) Cardiovascular safety of transdermal nicotine patches in patients with coronary artery disease who try to quit smoking. Cardiovasc Drugs Ther 12:239-244.

Wisborg K, Henriksen TB, Jespersen LB, Secher NJ (2000) Nicotine patches for pregnant smokers: a randomized controlled study. Obstet Gynecol 96:967-971.

Woolf KJ, Zabad MN, Post JM, McNitt S, Williams GC, Bisognano JD (2012) Effect of nicotine replacement therapy on cardiovascular outcomes after acute coronary syndromes. Am J Cardiol 110(7):968-970 doi:10.1016/j.amjcard.2012.05.028.

Zhu JL, Olsen J, Liew Z, Li J, Niclasen J, Obel C (2014) Parental smoking during pregnancy and ADHD in children: the Danish national birth cohort. Pediatrics 134(2):e382-e388 doi:10.1542/peds.2014-0213.
